# Mechanochemical generation of aryne†

**DOI:** 10.1039/d4sc03968h

**Published:** 2024-08-08

**Authors:** Qianqian Cheng, Guillaume De Bo

**Affiliations:** a Department of Chemistry, University of Manchester Manchester M13 9PL UK guillaume.debo@manchester.ac.uk

## Abstract

Mechanical force is unique in promoting unusual reaction pathways and especially for the generation of reactive intermediates sometimes inaccessible to other forms of activation. The mechanochemical generation of reactive species could find application in synthetic and materials chemistry alike. However, the nature of these reactive intermediates has been mostly limited to radicals or carbenes. Here, we present a new mechanophore that generates a reactive aryne intermediate upon dissociation of a benzocyclobutene (BCB) core *via* a force-promoted retro [2 + 2] cycloaddition.

## Abstract 2

An aryne intermediate is generated upon mechanical dissociation of a benzocyclobutene mechanophore. Such force-generated reactive species could be used in materials and synthesis.

## Graphical abstract

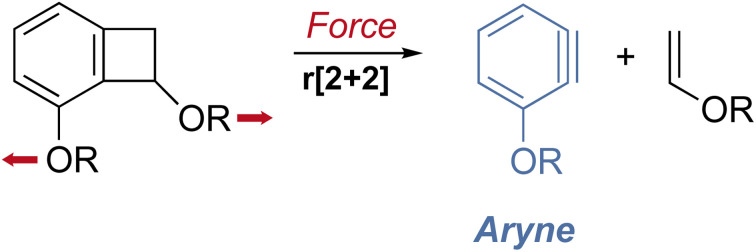

## Introduction

**Fig. 1:**
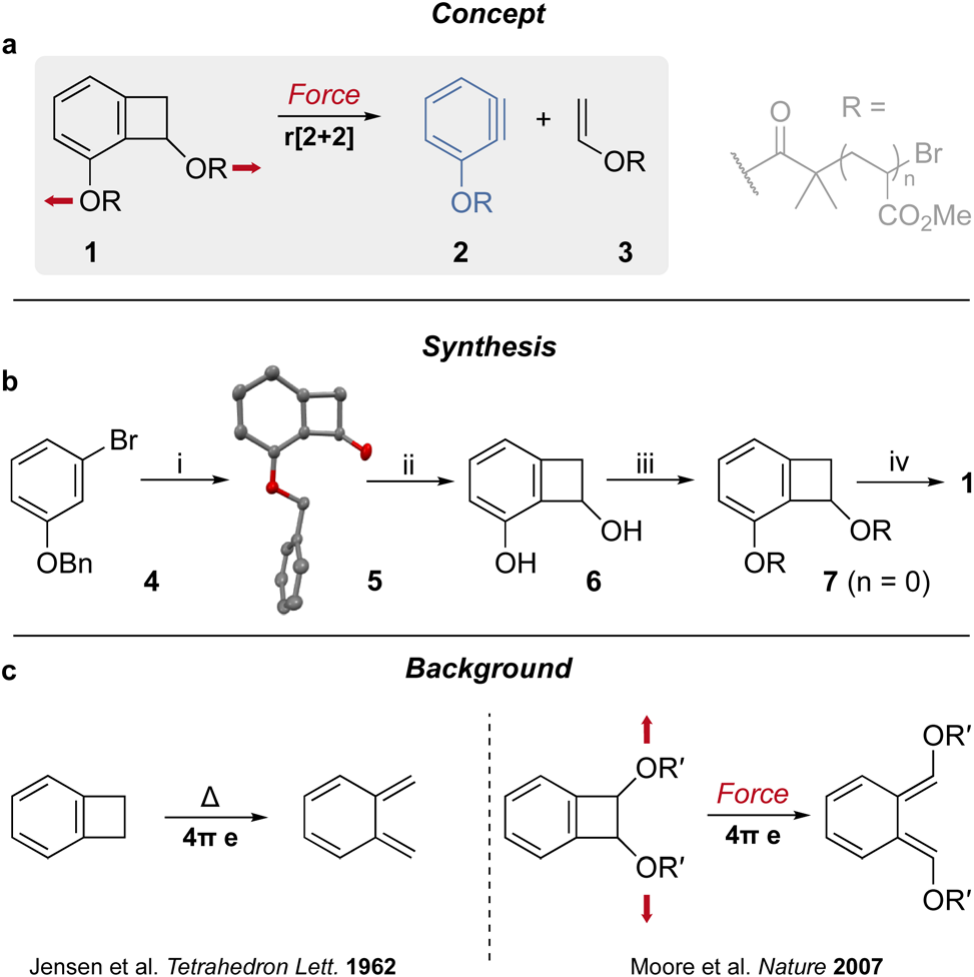
Mechanochemical generation of aryne. (a) Mechanical activation of benzocyclobutene mechanophore 1 leads to the generation of aryne 2. Red arrows indicate the direction of the force. (b) Synthesis of chain-centered mechanophore 1. Conditions: (i) LiTMP, THF, −78 °C, 1 h, 70%. (ii) H_2_, Pd/C, MeOH/AcOEt, r.t., 20 h, 60%. (iii) BiBB, Et_3_N, THF, r.t., 5 h, 58%. (iv) Methyl acrylate, CuBr_2_/Cu(0), Me_6_TREN, DMSO, r.t., 20 min. Solid-state structure (XRD) of intermediate 5 shown (hydrogen atoms omitted for clarity). (c) Previously reported thermal and mechanical electrocyclic ring opening of the BCB core.

Since their existence was demonstrated by Roberts' 1953 classic labeling experiment,^1^ arynes have proved to be very useful building blocks in organic synthesis as they are susceptible to nucleophilic additions, cycloadditions, and σ-bond insertion^2^ due to their strained triple bond.^3^ Consequently, several methods have been developed to generate this reactive species (usually requiring harsh conditions and/or an activated precursor), including by the action of UV light or of a strong base and, perhaps the most popular technique, by fluoride-promoted elimination of a triflate-silane precursor.^4^ Mechanical force can be used to stretch, and ultimately break, polymer chains. This usually proceeds *via* the homolytic scission of a covalent bond unless a mechanophore (force-sensitive molecule) is incorporated in the main chain.^5^ Such mechanophores have been designed to generate reactive species^6^ such as: radicals,^7–10^ carbenes,^11–15^ ylides,^16^ phenyl cations,^17^ carbanions,^13,14,18–20^ cumulated dienes,^15,21–23^ or unsaturated metal complexes.^11,12,18–20,24^ Benzocyclobutene (BCB) mechanophores have been instrumental in the early investigation of the chemistry of molecules under tension.^25^ Notably, they have been shown to experience anti-Woodward–Hoffmann ring opening upon stretching from the two non-aromatic carbons of the cyclobutene unit, which results in the formation of a transient *ortho*-quinodimethide intermediate (Fig. 1c).^26–31^ The same intermediate is formed during the thermal electrocyclic ring opening of BCB adducts (Fig. 1c).^32^ Here we propose to elicit a new mechanochemical behavior from the BCB core by placing the anchor points on each side of the ring junction (Fig. 1a). Pulling from these points result in the scission of the 4-membered ring, *via* a formal retro-[2 + 2] cycloaddition, to generate an enol ester and an aryne. We show that the activation of mechanophore 1 by ultrasonication (Fig. 1a) leads to the formation of aryne 2, *via* a formal retro-[2 + 2] cycloaddition of a benzocyclobutene unit, in otherwise mild and neutral conditions (MeCN, 5–10 °C). This new mechanochemical reactivity provides an easy access to a versatile reactive species that should find application in organic synthesis and materials chemistry.

## Results and discussion

The BCB core 5 was easily obtained as a single regioisomer by reacting bromobenzene derivative 4 with lithium 2,2,6,6-tetramethylpiperidide (LiTMP) and lithium ethanoate (generated *in situ*) following a known procedure (Fig. 1b).^33^ Interestingly, when the reaction is not caried out with fresh TMP, a dibenzocyclooctane dimer^34^ of 5 is obtained (S1, see ESI for details†). Hydrogenation of 5 delivers diol 6, which is further reacted with α-bromoisobutyryl bromide (BiBB) to afford bis-initiator 7. Chain-centered BCB 1 was obtained by single electron transfer living radical polymerization (SET-LRP)^35^ of methyl acrylate.

**Fig. 2:**
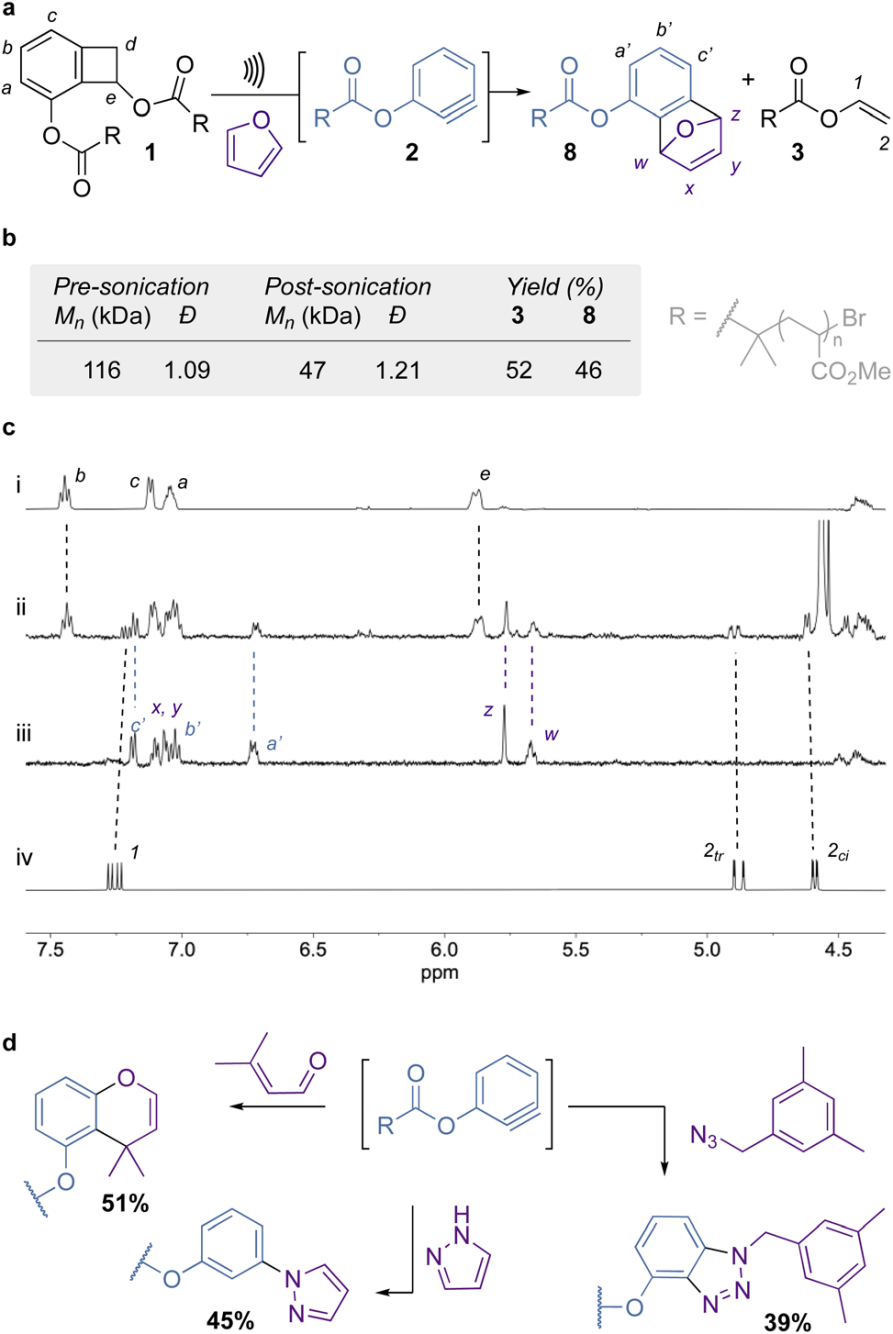
(a) Mechanical activation of chain-centered BCB mechanophore 1. Conditions: US (20 kHz, 13.0 W cm^−2^, 1 s ON/1 s OFF), furan (2000 eq.), CH_3_CN, 5–10 °C, 120 min. (b) Structural and activation parameters of the sonicated polymer. Yields determined by integrating protons 2_*cis*_ and *w* against proton *e* of the intact mechanophore for 3 and 8 respectively. (c) Partial ^1^H NMR (500 MHz, acetone-*d*_6_, 298 K, 1024 scans) spectra of 1 before (i) and after (ii) sonication along with reference compounds 8_ref_ (iii), an independently synthesised reference of compound 8, and vinyl pivalate (iv). (d) Scope of trapping agents. Percentages indicate the yield of trapped products generated from 1.

Mechanical activation of 1 was performed in acetonitrile at 5–10 °C, using high-intensity ultrasound at 13.0 W cm^−2^ (corresponding to an amplitude of 25%). A large excess of furan (2000 eq.) was added to trap the aryne as it formed (Fig. 2a–c). This trapping agent was chosen as it reacts readily with arynes, and the excess can be easily removed by evaporation at the end of the reaction. ^1^H NMR analysis of the sonicated sample confirms the scission of the 4-membered ring and the trapping of the aryne unit. This is evidenced by the shifting of the aromatic peaks (*a*–*a*′, *b*–*b*′, *c*–*c*′, Fig. 2c(i–iii)) and the emergence of the diagnostic bridging protons (*w*, *z*, Fig. 2c(i–iii)) upon formation of adduct 8, as well as the appearance of the olefinic protons of enol ester 3 (Fig. 2c(ii and iv)) in the post-sonication spectrum (Fig. 2c(ii)). The mechanophore activation proceeds with an efficiency of 52% (determined from the formation of enol ester 3), while the rest of the chains break in the PMA backbone (Fig. 2b). The generated aryne is efficiently trapped by furan with 46% of mechanophore 1 being converted into adduct 8 (Fig. 2a and b). A control polymer, where the mechanophore is placed near the chain end, showed no activation under the same conditions, further proving mechanical activation of the mechanophore (see ESI Section 5.6†). We then looked at alternative trapping agents that are representative of the scope of aryne reactivity: 3-methylbut-2-enal (heterodiene), pyrazole (nucleophile), and 3,5-dimethylbenzyl azide (1,3-dipole) to deliver the corresponding chromene, phenyl pyrazole, and triazole respectively (Fig. 2d). They present similar activation/trapping efficiencies, which illustrate the versatility of mechanically generated arynes.

**Fig. 3:**
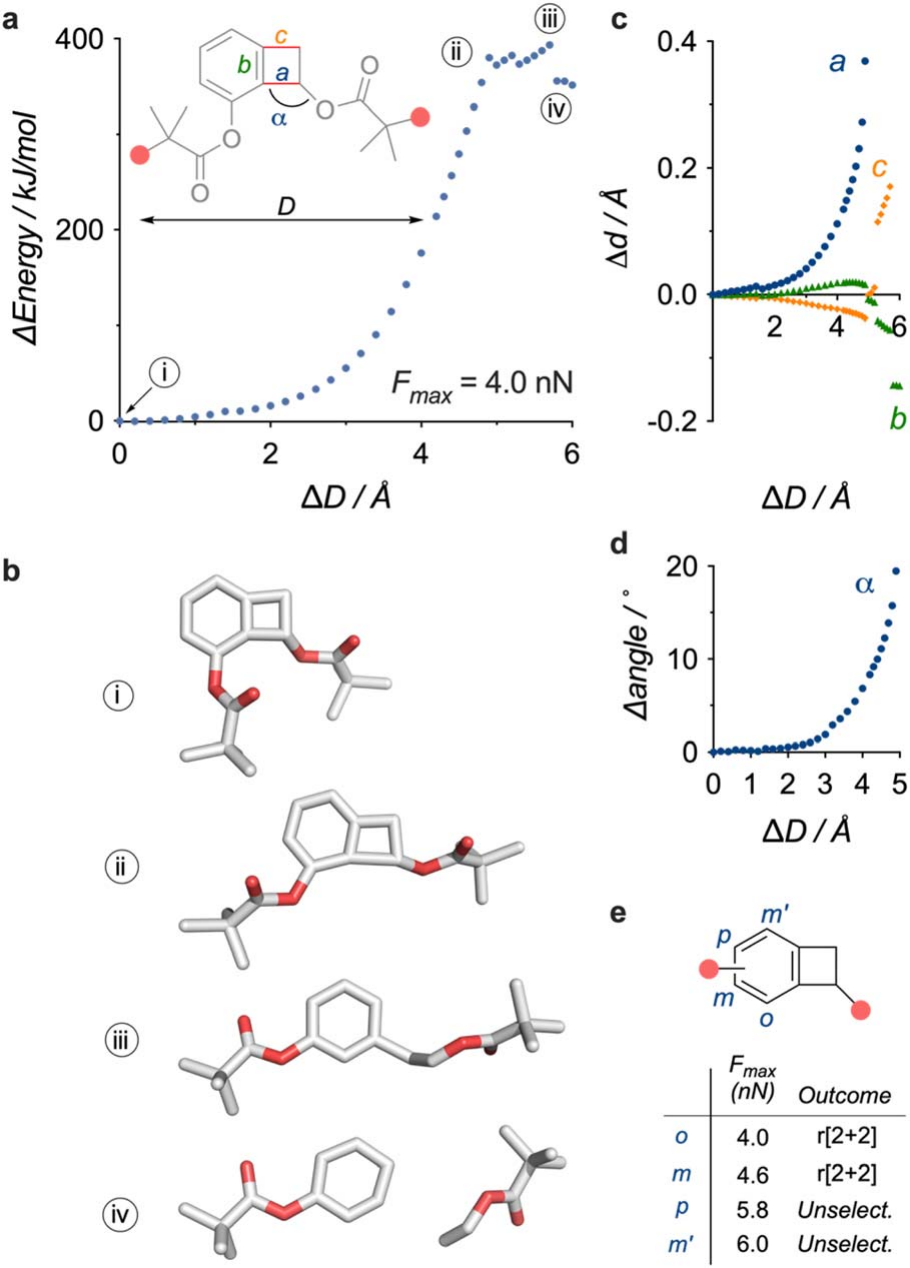
Computational investigation of the mechanochemical activation of the aryne-generating BCB mechanophore. (a) Evolution of energy upon simulated elongation (CoGEF, DFT B3LYP/6-31G*, vac.) of a model of BCB mechanophore 1. (b) Equilibrium geometries at *E*_0_ (i), *E*_max1_ (ii), *E*_max2_ (iii), and after dissociation (iv). (c) Elongation of bonds *a*, *b*, and *c*. (d) Opening of angle *α* upon simulated elongation of the same model up to *E*_max_. (e) Effect of regiochemistry on the activation of the aryne-generating BCB mechanophore.

The simulated elongation of a model of the BCB mechanophore offers some insight into the activation process (Fig. 3a–d). The elongation profile of this model (Fig. 3a), obtained from CoGEF calculations^36^ (DFT B3LYP/6-31G*), predicts the scission of C–C bond a ((iii), Fig. 3a and b), connecting the aromatic group to the cyclobutene anchor point, with a *F*_max_ of 4.0 nN, which is on par with the values calculated for BCB opening into *ortho*-quinodimethide.^37^ Further elongation delivers the aryne and the enol ester ((iv), Fig. 3a and b), and the emergence of the aryne triple bond can be visualised by the contraction of bond b (Fig. 3c), which culminates in the product with an overall contraction of 0.145 Å (from 1.397 Å to 1.252 Å). This dissociation is accompanied by a substantial amount of torsional stress (opening of angle *α*) as the cyclobutene anchor point aligns with the aromatic plane (Fig. 3d). Though, experimental^38^ and computational^39^ studies suggest that force-driven retrocycloaddition likely follow a sequential homolytic pathway, a different mechanism cannot be excluded as the CoGEF method does not account for dynamic or thermal effects.^40^ We also explored the effect of regiochemistry on the activation by varying the position of the anchor point on the aromatic ring (Fig. 3e). Only the 2 regioisomers with the anchor points closest to the putative scissile bond (*o*, *m*) display the expected *r*[2 + 2] reactivity, while the furthest 2 are predicted to cleave unselectively (*p*, *m*′, see ESI Section 8†), an observation consistent with previously reported geometrical effects in other mechanophores.^41–45^

## Conclusions

In conclusion, we have described the mechanochemical generation of an aryne species upon activation of a benzocyclobutene *via* a formal retro-[2 + 2] cycloaddition. We anticipate that the ability of mechanophores to generate reactive species should find application in materials chemistry (*e.g.* for self-healing as the aryne could insert into an adjacent chain through various pathways), and even in synthesis if it can be combined with a release mechanism.^46–48^

## Author contributions

Q. C. planned and carried out the experimental work. G. D. B. performed the computational investigation. G. D. B. directed the research. All authors contributed to the analysis of the results and the writing of the paper.

## Conflicts of interest

There are no conflicts to declare.

## Supplementary Material

SC-015-D4SC03968H-s001

SC-015-D4SC03968H-s002

## Data Availability

The data supporting this article have been included as part of the ESI.† Crystallographic data for 5 and S1† has been deposited at the CCDC under 2295694 and 2295695.
