# Supplementary material for: Mechanochemical generation of aryne
Source: Chem Sci. 2024 Aug 8;15(33):13181–4. doi: 10.1039/d4sc03968h (PMC11308379; doi:10.1039/d4sc03968h)
Supplement: SC-015-D4SC03968H-s001 [file SC-015-D4SC03968H-s001.pdf]

Supplementary Information (SI) for Chemical Science.
This journal is © The Royal Society of Chemistry 2024

Supporting Information

Mechanochemical Generation of Aryne

*Qianqian Cheng and Guillaume De Bo **

Department of Chemistry, University of Manchester, Oxford Road, Manchester, M13 9PL, UK

*Email: guillaume.debo@manchester.ac.uk

1. Table of Contents

Contents

1. TABLE OF CONTENTS 2
2. GENERAL EXPERIMENTAL DETAILS 3
3. SYNTHESIS OF MECHANOPHORE AND REFERENCE COMPOUNDS 4
   - *3.1 Synthesis of Mechanophore 7 .... 4*
   - *3.2 Synthesis of Reference Compound S5 .... 7*
   - *3.3 Synthesis of Reference Compound S9 .... 10*
   - *3.4 Synthesis of Reference Compound S11 .... 12*
   - *3.5 Synthesis of Reference Compound S13 .... 14*
   - *3.6 Synthesis of Control Compound S16 .... 16*
4. SYNTHESIS OF POLYMERS 19
   - *4.1 Representative Procedure for SET-LRP of Methyl Acrylate Using Mechanophore Initiators 19*
   - *4.2 Synthesis of Polymer 1 .... 19*
   - *4.3 Synthesis of Reference Polymer 8_{ref} 20*
   - *4.4 Synthesis of Reference Polymer S17 .... 20*
   - *4.5 Synthesis of Reference Polymer S18 .... 20*
   - *4.6 Synthesis of Reference Polymer S19 .... 21*
   - *4.7 Synthesis of Control Polymer S20 .... 21*
   - *4.8 SEC Data for Synthesised Polymers 21*
   - *4.9 SEC Traces for Polymers with Mechanophore 22*
   - *4.10 SEC Traces for Reference Polymers and Control Polymer 22*
5. MECHANOPHORE ACTIVATION VIA ULTRASOUND 23
   - *5.1 General Procedure for Sonication Experiments 23*
   - *5.2 Sonication of Polymer 1 with Furan 24*
   - *5.3 Sonication of Polymer 1 with S7 .... 25*
   - *5.4 Sonication of Polymer 1 with 3-Methyl-2-butenal 26*
   - *5.5 Sonication of Polymer 1 with 1H-Pyrazole 27*
   - *5.6 Sonication of Control Polymer S20 .... 28*
6. NMR SPECTRA 29
   - *6.1 Small Molecules NMR Spectra 29*
   - *6.2 Polymers NMR Spectra 47*
   - *6.3 Post-Sonication NMR Spectra 50*
7. CRYSTAL STRUCTURES 58
   - *7.1 Crystal Structure of 5 .... 58*
   - *7.2 Crystal Structure of Dimer S1 .... 58*
8. CALCULATIONS 59
   - *8.1 CoGEF of BCB model $1'_O$ 59*
   - *8.2 CoGEF of BCB model $1'_m$ 61*
   - *8.3 CoGEF of BCB model $1'_p$ 62*
   - *8.4 CoGEF of BCB model $1'_{m'}$ 63*
9. REFERENCES 64

2. General Experimental Details

Unless otherwise stated, all reagents and solvents were purchased from commercial suppliers and used without further purification. Dry solvents were obtained by passing through an activated alumina column on a Phoenix SDS solvent drying system (JC Meyer Solvent Systems, CA, USA). Compound **S2**, **S6** and **S7** was prepared according to a literature procedure. [1-3]

Analytical TLC was performed on precoated silica gel plates (0.25 mm thick, 60 F254, Merck, Germany) and observed under UV light or stained with a phosphomolybdic acid solution. Preparative TLC (PTLC) was performed on precoated silica gel plates: 500 µm or 2000 µm, UNIPLATE GF, Analtech Inc., DE, USA. Flash column chromatography was performed with silica gel 60 (230-400 mesh) from Sigma-Aldrich.

Size exclusion chromatography (SEC) analyses were performed in THF solution (1.0 mg mL^{-1}) at 40 °C using a GPC/SEC Agilent 1260 Infinity II with 2 × PL gel 10 µm mixed-C and a PL gel 500 Å column and equipped with a differential refractive index (DRI) detector employing narrow polydispersity polystyrene standards (Agilent Technologies) as a calibration reference. Samples were filtered through a Whatman Puradisc 4 mm syringe filter with 0.45 µm PTFE membrane before injection to equipment, and experiments were carried out with injection volume of 50 µL, flow rate of 1 mL min^{-1}. Results were analyzed using n-dodecane as internal marker using Agilent GPC/SEC Software Version 2.2.

Ultrasound experiments were performed using a Sonics VCX 500 ultrasonic processor equipped with a 13 mm diameter solid or replaceable-tip probe. The distance between the titanium tip and the bottom of the Suslick cell was 2 cm. The ultrasonic intensity was calibrated using the method outlined by Hickenboth et al.[4] The Suslick cells were fabricated by the Department of Chemistry glass workshop at the University of Manchester.

^{1}H and ^{13}C NMR spectra were recorded on a Bruker Avance III 500 MHz Prodigy instrument or a Bruker Avance III 400 MHz Prodigy instrument. Chemical shifts are reported in parts per million (ppm) from high to low frequency and referenced to the residual solvent resonance. Coupling constants (J) are reported in Hertz (Hz) and splitting patterns are designated as follows: b = broad, s = singlet, d = doublet, t = triplet, q = quartet, p = pentet and m = multiplet. ^{1}H and ^{13}C assignments were made using 1D or 2D NMR methods (HSQC, HMBC, COSY, NOESY).

Mass spectra were obtained through the Mass Spectrometry services in the Department of Chemistry at the University of Manchester.

Abbreviations: BiBB: bromoisobutyryl bromide; DCM: dichloromethane; DDQ: 2,3-Dichloro-5,6-dicyano-1,4-benzoquinone; DMSO: dimethylsulfoxide; ESI: electrospray ionization; EtOAc: ethyl acetate; HMDS: Hexamethyl disilylamine; HRMS: high resolution mass spectrometry; LiTMP: Lithium 2,2,6,6-tetramethylpiperidide; MA: methyl acrylate; MeCN: acetonitrile; MeOH: methanol; MS: mass spectrometry; Me_6TREN: tris[2-(dimethylamino)ethyl]amine; *n*-BuLi: *n*-Butyllithium solution; PE: petroleum ether; PMBCl; 4-methoxybenzyl chloride; THF: tetrahydrofuran; TLC: thin layer chromatography.

3. Synthesis of Mechanophore and Reference Compounds

3.1 Synthesis of Mechanophore 7

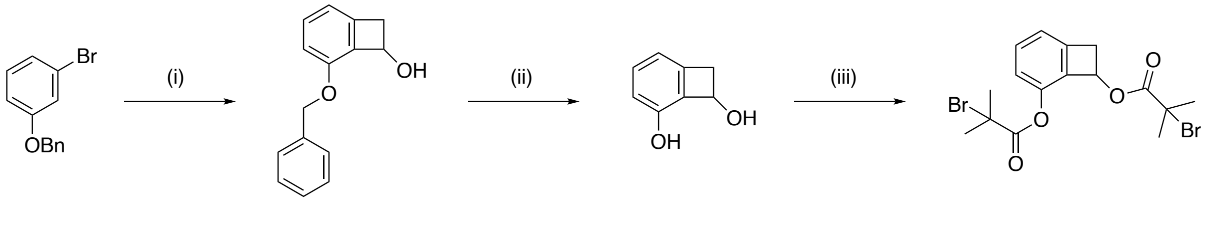

5 6 7

Figure S1. Synthetic route to **7**. Conditions: (i) LiTMP, $C_2H_4O.Li$, THF, -78 °C, 1 h, 70% yield; (ii) H_2 (gas), Pd/C powder, Methanol, EtOAc, rt, 20h, 60% yield; (iii) BiBB, Et_3N, THF, rt, 5 h, 58% yield.

3.1.1 Synthesis of 5 and S1

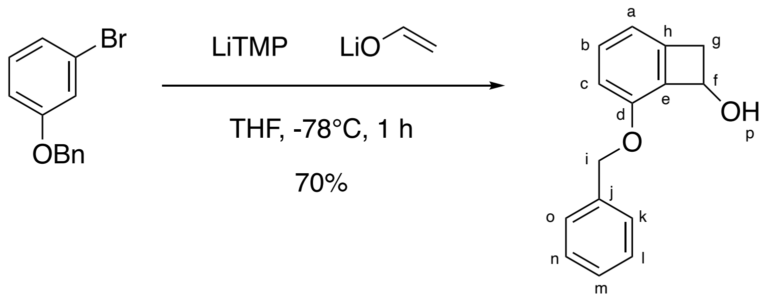

5

Synthesis of compound **5** was adapted from a literature procedure.[5] Preparation of $C_2H_4O.Li$: dry THF (25 mL) was added into a dried flask and cooled to 0° by ice bath. n-BuLi (2.5 M in hexane, 28.5 mmol, 1.5 eq.) was added dropwise. After that, the reaction was stirred for a further 16 h at room temperature under nitrogen supporting. LiTMP: 2,2,6,6-tetramethylpiperidine (24.7 mmol, 1.3 eq.) and dry THF (15 mL) were added into a dried flask. The system was cooled to 0° by ice bath, after cooling, n-BuLi (2.5 M in hexane, 22.8 mmol, 1.2 eq.) was then added dropwise and resulting mixture was stirred for 0.5 h. The LiTMP solution and the benzyne precursor (5 g, 19 mmol, 1.0 eq.) in THF (25 mL) were added to the enolate solution. The resulting solution was cooled to -78°C in an acetone/dry ice bath. After 30 mins stirring under the cooled system, the reaction was then quenched by aqueous NH_4Cl solution. The resulting mixture was then washed by water (15 mL) and extracted by ethyl acetate (30 mL) three times, then dried by $MgSO_4$. The crude was purified by column chromatography (silica gel, PE: acetone 30:1), to give **5** as white powder (2.99 g, 13 mmol, 70% yield).

^{1}H NMR (500 MHz, Acetone-d_6, 298 K) δ = 7.47 – 7.45 (m, 2H, $H_{o,k}$), 7.40 – 7.37 (m, 2H, $H_{n,l}$), 7.33 – 7.30 (m, 1H, H_m), 7.18 (dd, J = 8.4, 7.1 Hz, 1H, H_b), 6.73 (dd, J = 8.5, 0.8 Hz, 1H, H_c), 6.69 (dd, J = 7.1, 0.8 Hz, 1H, H_a), 5.41 (d, J = 12.2 Hz, 1H, H_i), 5.33 (d, J = 12.2 Hz, 1H, H_i), 5.23 (dd, J = 4.7, 2.0 Hz, 1H, H_f), 5.02 (d, J = 8.5 Hz, 1H, H_p), 3.46 (dd, J = 14.1, 4.6 Hz, 1H, H_g), 2.96 (dt, J = 14.1, 1.0 Hz, 1H, H_g).

^{13}C NMR (126 MHz, Acetone-d_6, 298 K) δ = 154.65 (C_d), 145.13 (C_h), 139.09 (C_j), 133.07 (C_e), 131.58 (C_b), 129.31 ($C_{n,l}$), 128.60 (C_m), 128.26 ($C_{o,k}$), 116.43 (C_a), 115.53 (C_c), 71.83 (C_i), 70.94 (C_f), 42.53 (C_g).

HRMS-ESI(+): 249.0884 $[M+Na]^+$, calculated for $C_{13}H_{12}O_2Na^+$: 249.0886

XRD: see section **7**.

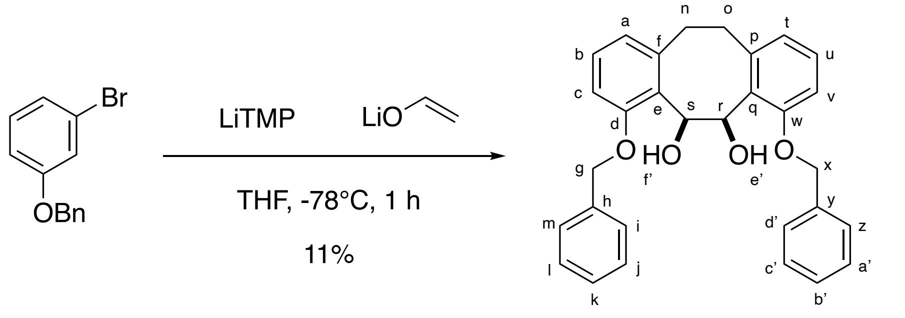

S1

The dimer structure of mechanophore (**S1**) was obtained when an old bottle of 2,2,6,6-tetramethylpiperidide was used in the procedure described above. The crude was purified by column chromatography (silica gel, PE: acetone 10:1), to give **S1** as white powder (0.19 g, 0.42 mmol, 11% yield).

^{1}H NMR (500 MHz, $CDCl_3$, 298 K) δ = 7.33 – 7.27 (m, 10H, $H_{i\text{-}m,\, z\text{-}d'}$), 6.98 (t, J = 7.9 Hz, 2H, $H_{b,u}$), 6.67 – 6.65 (m, 4H, $H_{a,c,t,v}$), 6.10 – 6.06 (m, 2H, $H_{r,s}$), 5.00 – 4.95 (m, 4H, $H_{g,x}$), 3.76 – 3.67 (m, 2H, $H_{n,o}$), 2.99 – 2.96 (m, 2H, $H_{e',f'}$), 2.91 – 2.83 (m, 2H, $H_{n,o}$).

^{13}C NMR (126 MHz, $CDCl_3$, 298 K) δ = 156.34 ($C_{d,w}$), 141.78 ($C_{f,p}$), 137.21 ($C_{h,y}$), 128.69 – 128.65 ($C_{m\text{-}i}$), 128.41 ($C_{b,u}$), 127.91 – 127.32 ($C_{y\text{-}d'}$), 124.27 ($C_{e,q}$), 110.52 ($C_{a,c,t,v}$), 70.90 ($C_{g,x}$), 68.09 ($C_{r,s}$), 34.44 ($C_{n,o}$).

HRMS-ESI(+): 475.1880 $[M+Na]^+$, calculated for $C_{30}H_{28}O_4Na^+$: 475.1880

XRD: see section 7.

3.1.2 Synthesis of 6

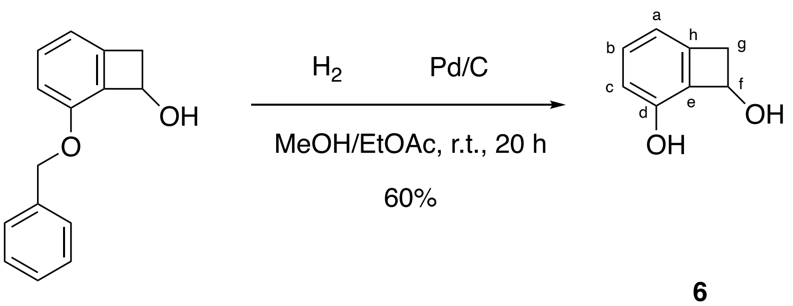

6

5 (1 g, 4.42 mmol, 1.0 eq.), Pd/C powder (14 mg), methanol (5 mL) and EtOAc (5 mL) were added together in a dried flask purged with nitrogen. Then the system was purged with hydrogen and the reaction was kept under a hydrogen atmosphere for 20 h. The mixture was filtrated, and the residual solid was washed with methanol. The organic fractions were

combined, concentrated to dryness, and purified by PTLC (silica, DCM: MeOH 30:1) to give **6** as white powder (0.36 g, 2.65 mmol, 60% yield).

^{1}H NMR (400 MHz, MeOD, 298 K) δ = 7.10 (dd, *J* = 8.3, 7.2 Hz, 1H, H_b), 6.61 – 6.57 (m, 2H, $H_{a,c}$), 5.21 (dd, *J* = 4.5, 1.8 Hz, 1H, H_f), 3.44 (dd, *J* = 13.9, 0.7 Hz, 1H, H_g), 2.89 (dt, *J* = 14.0, 0.9 Hz, 1H, H_g).

^{13}C NMR (101 MHz, MeOD, 298 K) δ = 152.59 (C_d), 145.04 (C_h), 133.12 (C_e), 132.02 (C_b), 115.60 (C_a), 115.07 (C_c), 70.03 (C_f), 42.06 (C_g).

HRMS-ESI(-): 135.0457 [M-H]$^-$, calculated for $C_8H_8O_2^-$: 135.0450

3.1.3 Synthesis of 7

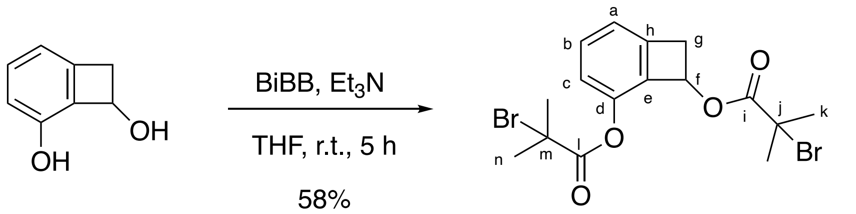

7

6 (100 mg, 0.735 mmol, 1.0 eq.), BiBB (363 µL, 2.94 mmol, 4.0 eq.), Et_3N (410 µL, 2.94 mmol, 4.0 eq.) were added into THF (3 mL) and the resulting mixture was stirred for 5h. The resulting mixture was then washed by water (10 mL) and extracted by DCM (20 mL) three times, then dried by $MgSO_4$. The crude was purified by PTLC (silica, PE: EtOAc 10:1) to get **7** as white powder (185 mg, 0.426 mmol, 58% yield).

^{1}H NMR (400 MHz, Acetone-d_6, 298 K) δ = 7.49 (ddd, *J* = 8.1, 7.3, 0.6 Hz, 1H, H_b), 7.18 (dd, *J* = 7.3, 0.8 Hz, 1H, H_a), 7.05 (dd, *J* = 8.4, 0.8 Hz, 1H, H_c), 5.97 (dd, *J* = 4.6, 1.9 Hz, 1H, H_f), 3.74 (ddt, *J* = 14.7, 4.7, 0.8 Hz, 1H, H_g), 3.26 (ddt, *J* = 14.7, 2.0, 0.9 Hz, 1H, H_g), 2.09 – 2.08 (m, 6H, $H_{k\ or\ n}$), 1.95 – 1.94 (m, 6H, $H_{k\ or\ n}$).

^{13}C NMR (126 MHz, Acetone-d_6, 298 K) δ = 171.69, 169.77 ($C_{i,l}$), 145.74 (C_d), 145.25 (C_h), 134.94 (C_e), 133.16 (C_b), 122.58 (C_c), 120.42 (C_a), 72.73 (C_f), 57.19, 56.95 ($C_{j,m}$), 39.48 (C_g), 31.01, 30.99, 30.96 ($C_{k,n}$).

HRMS -ESI(+): 454.9464 [M+Na]$^+$, calculated for $C_{16}H_{18}Br_2O_4Na^+$: 454.9481.

3.2 Synthesis of Reference Compound S5

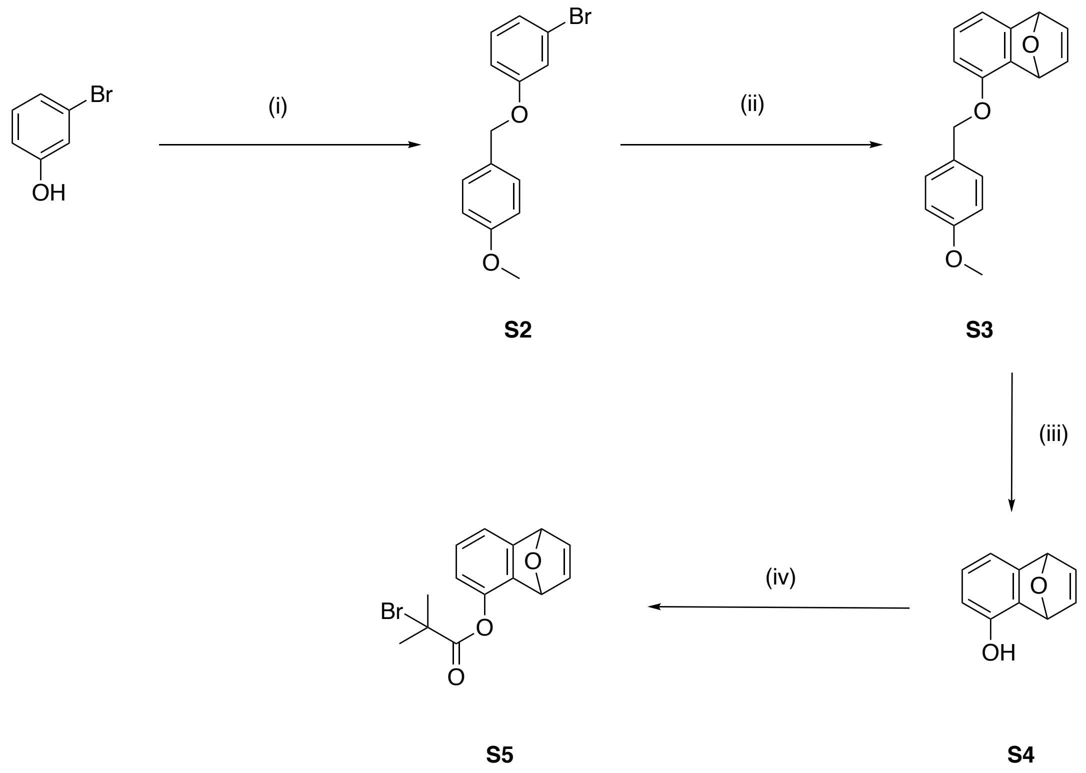

Figure S2. Synthetic route to **S5**. Conditions: (i) K_2CO_3, PMBCl, Tetrabutylammonium iodide, anhydrous THF, reflux, 24 h, 59% yield; (ii) LiTMP, Furan, THF, -78 °C, 1 h, 31% yield; (iii) DDQ, DCM, H_2O, rt, 24h, 53% yield; (iv) BiBB, Et_3N, THF, rt, 5 h, 40% yield.

3.2.1 Synthesis of S2

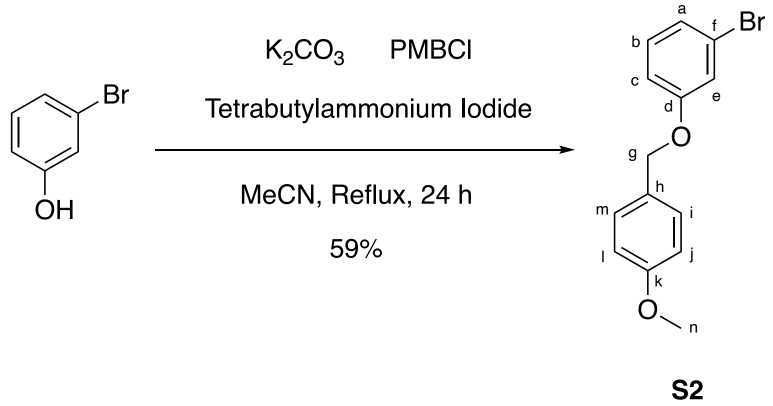

Compound **S2** was prepared according to literature procedures.[1] 3-Bromophenol (10 g, 57 mmol, 1.0 eq.), K_2CO_3 (24 g, 0.17 mol, 3.0 eq.), PMBCl (17 g, 0.11 mol, 1.5 eq.) and tetrabutylammonium iodide (2.13 g, 5.78 mmol, 0.1 eq.) were added into anhydrous THF (60 mL). The system was heated to reflux overnight. The mixture was filtrated and the residual solid was washed with acetone and DCM. The organic fractions were combined, concentrated to dryness, and purified by column chromatography (silica gel, PE: acetone 20:1), to give **S2** as white powder (9.6 g, 57.8 mmol, 59% yield). The ^1H NMR spectroscopic data is consistent with previous publication.[1]

^{1}**H NMR** (500 MHz, Acetone-d_6, 298 K) δ = 7.42 – 7.39 (m, 2H, $H_{m,i}$), 7.23 (t, J = 8.2 Hz, 1H, H_b), 7.19 (dd, J = 2.4, 1.8 Hz, 1H, H_e), 7.11 (ddd, J = 7.9, 1.8, 0.9 Hz, 1H, H_a), 7.01 (ddd, J = 8.4, 2.4, 0.9 Hz, 1H, H_c), 6.97 – 6.94 (m, 2H, $H_{l,j}$), 5.06 (s, 2H, H_g), 3.81 (s, 3H, H_n).

3.2.2 Synthesis of S3

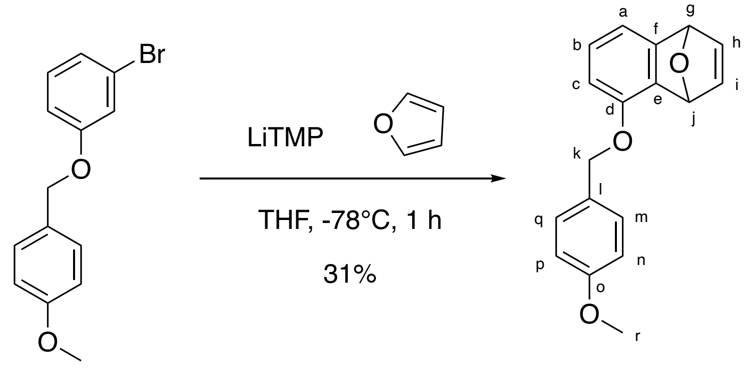

S3

Preparation of LiTMP: 2,2,6,6-tetramethylpiperidine (24.7 mmol, 1.3 eq.) and dry THF (15 mL) were added into a dried flask. The system was cooled to 0° then n-BuLi (2.5 M in hexane, 22.8 mmol, 1.2 eq.) was added dropwise, and resulting mixture was stirred for 0.5 h. The LiTMP solution and **S2** (4 g, 13.6 mmol, 1.0 eq.) in THF (13 mL) were added into the furan (1.39 g, 20.4 mmol, 1.5 eq.) in THF (2 mL). The resulting mixture solution was cooled to -78°C in an acetone/dry ice bath. After 15 mins stirring under the cooled system, the reaction was quenched by aqueous NH_4Cl solution. The mixture was then washed with water (15 mL) and extracted with ethyl acetate (30 mL) three times. The combined organic fractions were washed with brine, dried over $MgSO_4$, filtered, and concentrated to dryness. The crude was purified by column chromatography (silica gel, PE: acetone 10:1), to give **S3** as white powder (1.19g, 1.77 mmol, 31% yield).

^{1}**H NMR** (500 MHz, Acetone-d_6, 298 K) δ = 7.42 – 7.39 (m, 2H, $H_{m,q}$), 7.04 – 7.01(m, 2H, $H_{h,i}$), 6.96 – 6.90 (m, 4H, $H_{b,c,n,p}$), 6.71 (dd, J = 7.9, 1.2 Hz, 1H, H_a), 5.87 (s, 1H, H_g), 5.67 (s, 1H, H_j), 5.07 (q, J = 11.4 Hz, 2H, H_k), 3.81 (s, 3H, H_r).

^{13}**C NMR** (126 MHz, Acetone-d_6, 298 K) δ = 160.50 (C_o), 153.26 (C_f), 153.06 (C_d), 143.92 (C_h), 143.70 (C_i), 136.95 (C_e). 130.36 (C_l), 130.14 ($C_{m,q}$), 127.45 ($C_{b,c}$), 114.67 ($C_{n,p}$), 113.15 (C_a), 83.14 (C_j), 80.77 (C_g), 71.09 (C_k), 55.54 (C_r).

HRMS-ESI(+): 303.0982 [M+Na]$^+$, calculated for $C_{18}H_{16}O_3Na^+$: 303.0992

3.2.3 *Synthesis of S4*

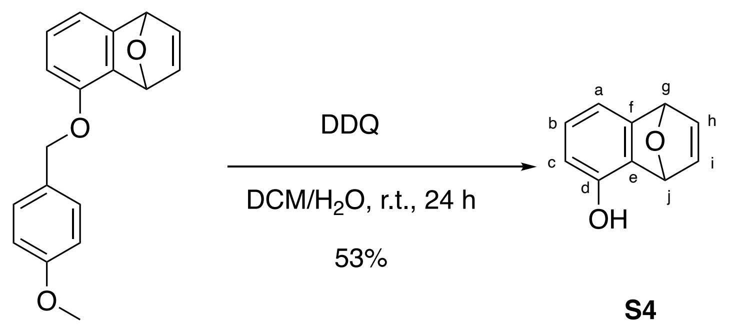

S4

S3 (100 mg, 0.36 mmol, 1.0 eq.) and DDQ (121.5 mg, 0.54 mmol, 1.5 eq.) were added into a dried flask, DCM (2 mL) and water (0.12 mL) was added. The resulting mixture was stirred for 24 h. The mixture was filtrated, and the residual solid was washed with DCM. The organic fractions were combined, concentrated to dryness, and purified by column chromatography (silica gel, PE: acetone 3:1), to give **S4** as white powder (30.3 mg, 0.189 mmol, 53% yield).

^{1}H NMR (400 MHz, Acetone-d_6, 298 K) δ = 7.04 – 7.00 (m, 2H, $H_{h,i}$), 6.84 – 6.78 (m, 2H, $H_{a,b}$), 6.48 (dd, *J* = 7.8, 1.2 Hz, 1H, H_c), 5.87 (s, 1H, H_j), 5.64 (s, 1H, H_g).

^{13}C NMR (101 MHz, Acetone-d_6, 298 K) δ = 152.97 (C_f), 150.86 (C_d), 143.60, 143.55 ($C_{h,i}$), 134.28 (C_e), 127.14 (C_b), 115.19 (C_c), 113.33 (C_a), 83.15 (C_g), 80.31 (C_j).

HRMS-ESI(-): 159.0440 [M-H]$^-$, calculated for $C_{10}H_7O_2^-$: 159.0452

3.2.4 *Synthesis of S5*

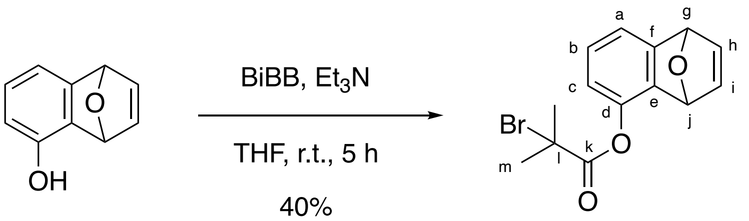

S5

S4 (30 mg, 0.19 mmol, 1.0 eq.), BiBB (46.3 µL, 0.37 mmol, 2.0 eq.), Et_3N (52.2 µL, 0.37 mmol, 2.0 eq.) were added into THF (2 mL). The resulting mixture was stirred for 5h. The resulting mixture was then washed by water (5 mL) and extracted by DCM (10 mL) three times, then dried by $MgSO_4$. The crude was purified by PTLC (silica, PE: EtOAc 20:1) to get **S5** as white powder (23 mg, 0.075 mmol, 40% yield).

^{1}H NMR (400 MHz, Acetone-d_6, 298 K) δ = 7.20 – 7.25 (m, 1H, H_a), 7.10 – 7.03 (m, 3H, $H_{b,h,i}$), 6.74 (dd, *J* = 8.3, 0.7 Hz, 1H, H_c), 5.80 (s, 1H, H_g), 5.73 (s, 1H, H_j), 2.12 (m, 3H, H_m), 2.11 (m, H, H_m)

^{13}C NMR (101 MHz, Acetone-d_6, 298 K) δ = 170.53 (C_k), 153.59 (C_d), 144.39 (C_f), 144.07 (C_h), 143.36 (C_i), 141.82 (C_e), 127.73 (C_b), 118.92 ($C_{a,c}$), 83.17 (C_g), 80.94 (C_j), 57.23 (C_l), 30.79 – 30.77 (C_m)

HRMS -ESI(+): 330.9940 [M+Na]$^+$, calculated for $C_{14}H_{13}BrO_3Na^+$: 330.9949.

3.3 Synthesis of Reference Compound S9

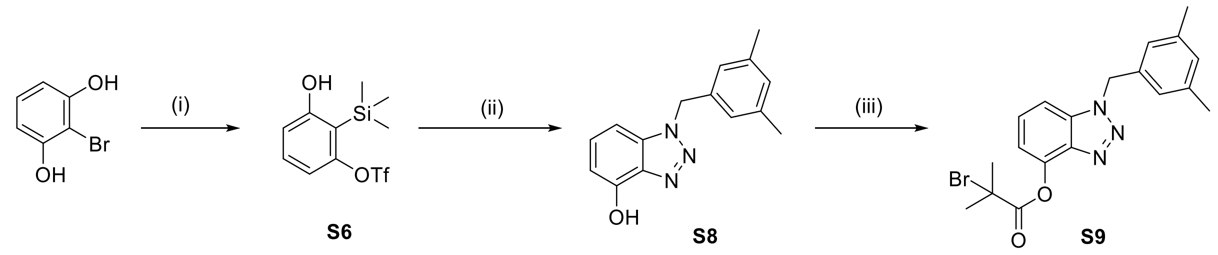

Figure S3. Synthetic route to **S8**. Conditions: (i) i: HMDS, THF, reflux, 12h, ii: n-BuLi, THF, -78°C 1h, iii: Tf2O, THF 1h; 41% yield (ii)**S7**, CsF, MeCN, rt, 2 h, 74% yield; (iii) BiBB, Et_3N, THF, rt, 3 h, 53% yield.

3.3.1 Synthesis of S6

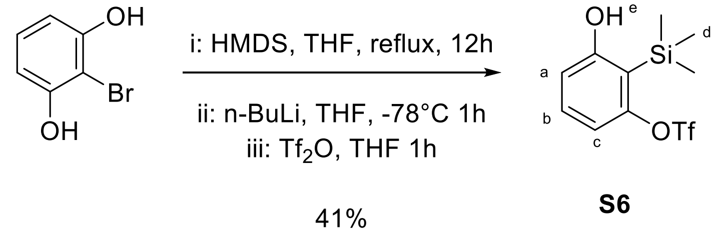

Compound **S6** was prepared according to literature procedures.[2] HMDS (1.35 mL, 5.9 mmol, 2.2 eq.) was added into a solution of 2-bromoresorcinol (500 mg, 2.7 mmol, 1.0 eq.) in dry THF (5 mL) under inert atmosphere. The solution was heated to reflux for 12 hours. After that, the solution was concentrated by rotary evaporator and dried with vacuum pump for 2 hours. The dried product was then dissolved in dry THF (5 mL) under inert atmosphere again. The solution was cooled to -78°C in an acetone/dry ice bath. n-BuLi (2.5 M in hexane, 1.16 mL, 2.9 mmol, 1.1eq.) was added into the solution dropwise. The solution was keep stirring for 1 hour. After that, trifluoromethanesulfonic anhydride (Tf_2O) (0.67 mL, 4.0 mmol, 1.5eq.) was added into the reaction solution and keep stirring for 1 hour. The solution was quenched with $NaHCO_3$ (15 mL) and extracted with ethyl acetate (15 mL) three times. The combined organic layers were washed by brine (15 mL), dried over $MgSO_4$, and concentrated to dryness. The crude product was purified by column chromatography (silica gel, PE: ethyl acetate 30:1), to give **S6** as pale yellow solid (338 mg, 1.07 mmol, 41% yield). The ^{1}H NMR spectroscopic data is consistent with previous publication.[2]

^{1}H NMR (500 MHz, Acetone-d_6, 298 K) δ = 9.36 (bs, 1H, H_e) 7.36 (t, J = 8.2 Hz, 1H, H_b), 6.93 (dd, J = 8.1, 0.8 Hz, 1H, H_a), 6.87 (d, J = 8.3 Hz, 1H, H_c), 0.40 (s, 9H, H_d).

3.3.2 *Synthesis of S7*

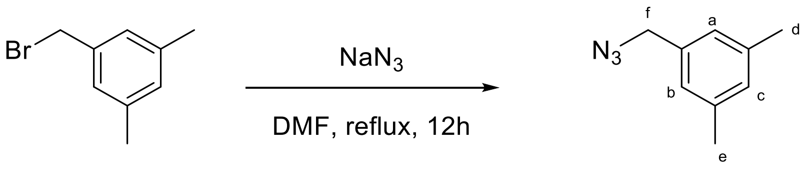

Compound **S2** was prepared according to literature procedures.[3] 3,5-Dimethylbenzyl bromide (1 g, 5.1 mmol, 1.0 eq.) is dissolved in DMF (10 mL) under inert atmosphere, sodium azide (0.49 g, 7.6 mmol, 1.5 eq.) was added. The solution then heated to reflux and keep for 12 h. The reaction then was quenched by water (10 mL) and extracted with ethyl acetate (15 mL) three times. The combined organic layers were washed by brine (20 mL), dried by $MgSO_4$, and concentrated to dryness. The crude product was purified by column chromatography (silica gel, PE: ethyl acetate 80:1), to give **S7** as colourless oily liquid (653 mg, 4.05 mmol, 80% yield). The ^{1}H NMR spectroscopic data is consistent with previous publication.[3]

^{1}H NMR (400 MHz, Acetone-d_6, 298 K) δ = 6.99 (s, 3H, $H_{a,b,c}$), 4.33 (s, 2H, H_f), 2.30 (s, 6H, $H_{d,e}$).

3.3.3 *Synthesis of S8*

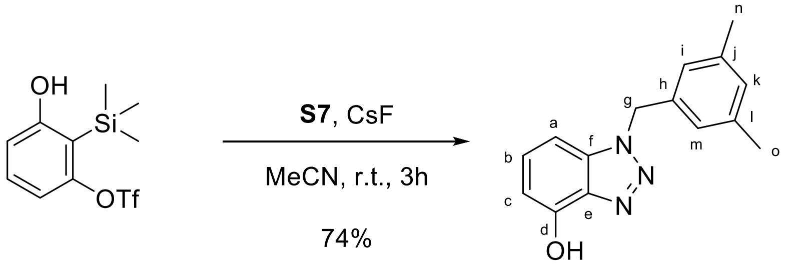

S6 (30 mg, 0.10 mmol, 1.0 eq.), **S7** (30.8 mg, 0.20 mmol, 2.0 eq.) and CsF (36.3 mg, 0.24 mmol, 2.5 eq.) was added in to MeCN (2 mL), the solution was stirred for 3 hours at room temperature. The colour of the mixture solution has changed from pale blue to dark green. The reaction then was quenched by NH_4Cl and extracted with ethyl acetate (5 mL) three times. The combined organic layers were washed by brine (5 mL), dried by $MgSO_4$, and concentrated to dryness. dried by $MgSO_4$ and concentrated to dryness. The crude was purified by PTLC (silica, PE: EtOAc 8:1) to get **S8** as white powder (17.8 mg, 0.070 mmol, 74% yield).

^{1}H NMR (400 MHz, Acetone-d_6, 298 K) δ = 9.51 (bs, 1H, O*H*), 7.28 (t, *J* = 8.3 Hz, 1H, H_b), 7.10 (dd, *J* = 8.3, 0.8 Hz, 1H, H_a), 6.97 (s, 2H, $H_{i,m}$), 6.94 (s, 1H, H_k), 6.71 (dd, *J* = 7.7, 0.8 Hz, 1H, H_c), 5.8 (s, 2H, H_g), 2.23 (s, 6H, $H_{n,o}$)

^{13}C NMR (101 MHz, Acetone-d_6, 298 K) δ = 150.31 (C_d), 139.24 (C_e), 136.88 (C_f), 130.53 (C_k), 129.50 (C_b), 126.41 ($C_{i,m}$), 107.95 (C_c), 101.80 (C_a), 52.33 (C_g), 21.30 ($C_{n,l}$).

HRMS -ESI(+): 276.1114 [M+Na]$^+$, calculated for $C_{15}H_{15}N_3ONa^+$: 276.1107

3.3.4 Synthesis of S9

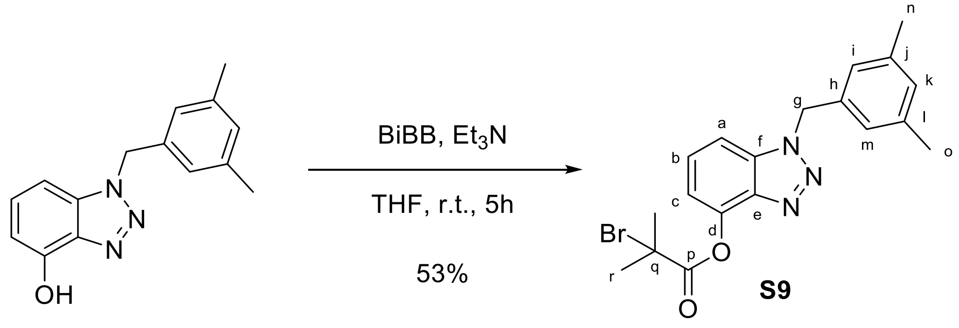

S8 (8 mg, 0.32 mmol, 1.0 eq.), BiBB (7.8 µL, 0.063 mmol, 2.0 eq.), Et_3N (8.8 µL, 0.063 mmol, 2.0 eq.) were added into THF (1 mL). The resulting mixture was stirred for 5h. The resulting mixture was then washed by water (5 mL) and extracted by DCM (10 mL) three times, then dried by $MgSO_4$. The crude was purified by PTLC (silica, PE: EtOAc 10:1) to get **S9** as white powder (6.71 mg, 0.017 mmol, 53% yield).

^{1}H NMR (500 MHz, Acetone-d_6, 298 K) δ = 7.67 (dd, J = 8.5, 0.8 Hz, 1H, H_a) 7.54 (t, J = 8.5, 7.4 Hz, 1H, H_b), 7.19 (dd, J = 7.5, 0.7Hz, 1H, H_c), 7.02 (s, 2H, $H_{i,m}$), 6.95 (s, 1H, H_k), 5.91 (s, 2H, H_g), 2.24 (s, 6H, $H_{n,o}$), 2.18 (s, 6H, $H_{s,r}$).

^{13}C NMR (126 MHz, Acetone-d_6, 298 K) δ = 170.41 (C_q), 142.65 (C_f), 140.26 (C_e), 139.37 ($C_{j,l}$), 136.41 (C_h), 136.01 (C_d), 130.72 (C_k), 128.64 (C_b), 126.54 ($C_{i,m}$), 116.28 (C_c), 109.75 (C_a), 56.51 (C_p, 52.70 (C_g), 31.21 ($C_{s,r}$), 21.27($C_{n,o}$).

HRMS -ESI(+): 402.0817[M+H]$^+$, calculated for $C_{19}H_{21}BrN_3O_2^+$: 402.0812.

3.4 Synthesis of Reference Compound S11

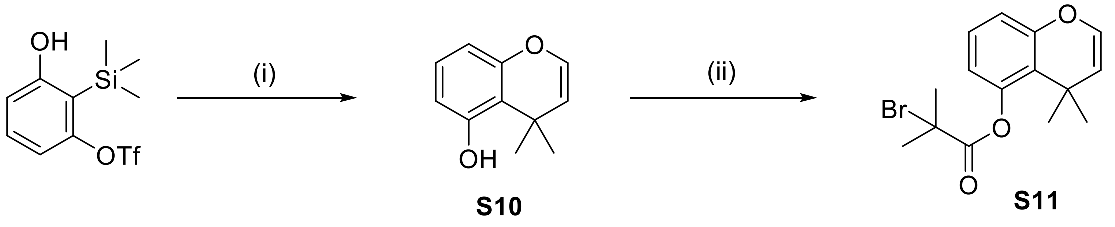

Figure S4. Synthetic route to **S11**. Conditions: (i) 3-Methyl-2-butenal, CsF, MeCN, rt, 2 h, 58% yield; (iii) BiBB, Et_3N, THF, rt, 3 h, 59% yield.

3.4.1 Synthesis of S10

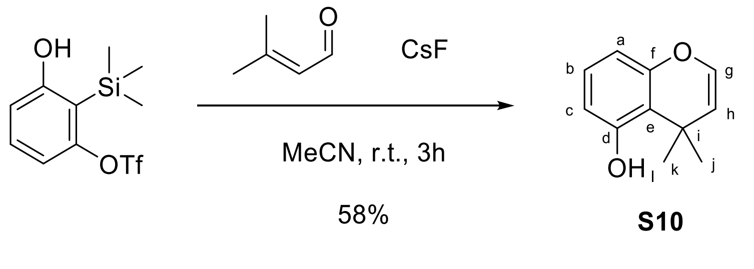

S6 (30 mg, 0.10 mmol, 1.0 eq.), 3-Methyl-2-butenal (20.1 mg, 0.19 mmol, 2.0 eq.) and CsF (36.3 mg, 0.24 mmol, 2.5 eq.) was added in to MeCN (2 mL), the solution was stirred for 3

hours at room temperature. The colour of the mixture solution has changed from pale blue to dark green. The reaction then was quenched by NH_4Cl and extracted with ethyl acetate (5 mL) three times. The combined organic layers were washed by brine (5 mL), dried by $MgSO_4$, and concentrated to dryness. dried by $MgSO_4$ and concentrated to dryness. The crude was purified by PTLC (silica, DCM:MeOH 70:1) to get **S10** as pale yellow solid (9.8 mg, 0.056 mmol, 58% yield).

^{1}H NMR (400 MHz, Acetone-d_6, 298 K) δ = 8.48 (bs, 1H, H_l), 6.89 (t, J = 8.1 Hz, 1H, H_b), 6.67 (dd, J = 9.9, 0.7Hz, 1H, H_g), 6.39 (dd, J = 8.1, 1.0 Hz, 1H, $H_{a\text{ or }c}$), 6.25(dt, J = 8.1, 0.9 Hz, 1H, $H_{c\text{ or }a}$), 5.61(t, J = 9.9 Hz, 1H, H_h), 1.37(s, 6H, $H_{k,j}$).

^{13}C NMR (101 MHz, Acetone-d_6, 298 K) δ = 155.01 (C_f), 154.07 (C_d), 129.76 (C_b), 129.30 (C_h), 117.88 (C_g), 110.41 (C_e), 108.74 ($C_{a\text{ orc}}$), 108.48 ($C_{c\text{ or }a}$), 76.18 (C_i), 28.05 ($C_{k,j}$).

HRMS -ESI(-):175.0769 $[M-H]^-$, calculated for $C_{11}H_{11}O_2^-$: 175.0765.

3.4.2 Synthesis of S11

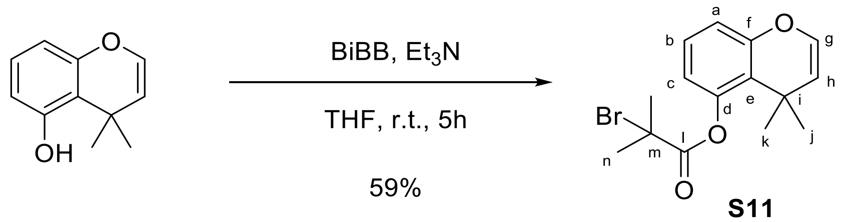

S10 (5 mg, 0.028 mmol, 1.0 eq.), BiBB (7.0 µL, 0.057 mmol, 2.0 eq.), Et_3N (7.9 µL, 0.057 mmol, 2.0 eq.) were added into THF (1 mL). The resulting mixture was stirred for 5h. The resulting mixture was then washed by water (5 mL) and extracted by DCM (10 mL) three times, then dried by $MgSO_4$. The crude was purified by PTLC (silica, PE: EtOAc 30:1) to get **S11** as pale yellow solid (5.68 mg, 0.017 mmol, 59% yield).

^{1}H NMR (400 MHz, Acetone-d_6, 298 K) δ = 7.16 (t, J = 8.1 Hz, 1H, H_b), 6.71 – 6.64 (m, 2H, $H_{a,c}$), 6.53 (dd, J = 9.9, 0.8 Hz, 1H, H_g), 5.81 (d, J = 10.0 Hz, 1H, H_h), 2.10 (s, 6H, H_n), 1.41 (s, 6H, $H_{k,j}$).

^{13}C NMR (101 MHz, Acetone-d_6, 298 K) δ = 170.39 (C_l), 154.82 (C_f), 147.37 (C_d), 132.76 (C_h),129.67 (C_b), 116.44 (C_g), 115.48 (C_e), 115.08 ($C_{a\text{ or }c}$), 114.70 ($C_{c\text{ or }a}$), 77.02 (C_i), 57.09 (C_m), 30.86 (C_n), 27.99 ($C_{k,j}$).

HRMS -APCI(+): 325.0434$[M+H]^+$, calculated for $C_{15}H_{18}BrO_3^+$: 325.0434.

3.5 Synthesis of Reference Compound S13

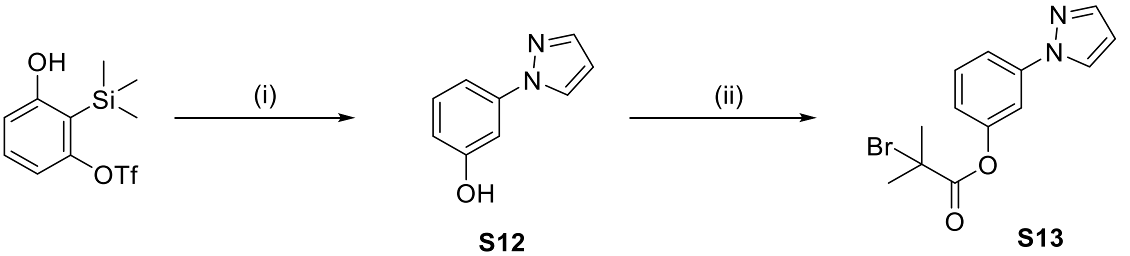

S12 **S13**

Figure S5. Synthetic route to **S13**. Conditions: (i) 1H-Pyrazole, CsF, MeCN, rt, 2 h, 58% yield; (iii) BiBB, Et_3N, THF, rt, 3 h, 51% yield.

3.5.1 Synthesis of S12

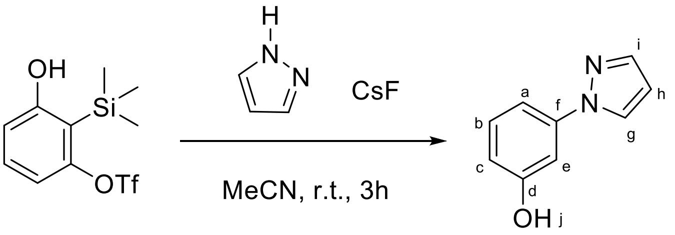

58% **S12**

S6 (30 mg, 0.10 mmol, 1.0 eq.), 1H-Pyrazole (11.8 mg, 0.19 mmol, 2.0 eq.) and CsF (36.3 mg, 0.24 mmol, 2.5 eq.) was added in to MeCN (2 mL), the solution was stirred for 3 hours at room temperature. The colour of the mixture solution has changed from pale green to dark green. The reaction then was quenched by NH_4Cl and extracted with ethyl acetate (5 mL) three times. The combined organic layers were washed by brine (5 mL), dried by $MgSO_4$, and concentrated to dryness. dried by $MgSO_4$ and concentrated to dryness. The crude was purified by PTLC (silica, PE: EtOAc 7:1) to get **S12** as pale yellow solid (8.8 mg, 0.055 mmol, 58% yield).

^{1}H NMR (400 MHz, Acetone-d_6, 298 K) δ = 8.73 (bs, 1H, H_j), 8.25 (d, J = 2.5 Hz, 1H, H_g) 7.66 (d, J = 1.7 Hz, 1H, H_i), 7.36 (d, J = 2.4, 1.0 Hz, 1H, H_a), 7.31 – 7.26 (m, 2H, $H_{b,c}$), 6.80 – 6.74 (m, 1H, H_e), 6.47 (dd, J = 2.5, 1.8 Hz 1H, H_h).

^{13}C NMR (101 MHz, Acetone-d_6, 298 K) δ = 159.34 (C_d), 142.49 (C_f), 141.46 (C_i), 131.17 (C_b), 127.84 (C_g), 114.08 (C_e), 110.45 (C_c), 108.27 (C_h), 106.91 (C_a).

HRMS-APCI(+): 161.0712 $[M+H]^+$, calculated for $C_9H_9N_2O^+$: 161.0709.

3.5.2 Synthesis of S13

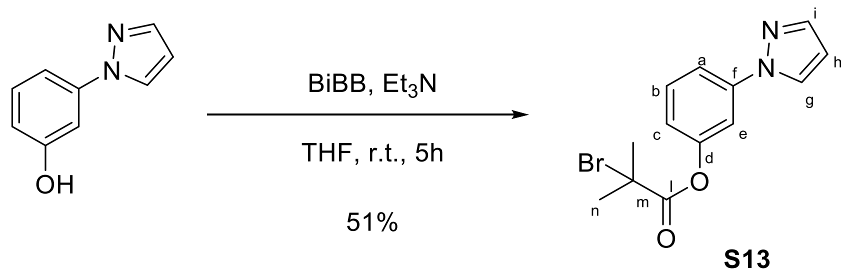

S13

S10 (5 mg, 0.041 mmol, 1.0 eq.), BiBB (10.2 µL, 0.082 mmol, 2.0 eq.), Et_3N (11.5 µL, 0.082 mmol, 2.0 eq.) were added into THF (1 mL). The resulting mixture was stirred for 5h. The resulting mixture was then washed by water (5 mL) and extracted by DCM (10 mL) three times, then dried by $MgSO_4$. The crude was purified by PTLC (silica, PE: EtOAc 10:1) to get **S11** as pale yellow solid (6.81 mg, 0.021 mmol, 51% yield).

^{1}H NMR (500 MHz, Acetone-d_6, 298 K) δ = 8.38 (d, J = 2.6 Hz 1H, H_i), 7.79 (ddd, 1H, J = 8.2, 2.2, 0.9 Hz H_a), 7.73 (t, J = 2.2 Hz, 1H, H_e), 7.71 (d, J = 1.7 Hz, 1H, H_g), 7.57 (t, J = 8.2 Hz, 1H, H_b), 7.12 (ddd, J = 8.1, 2.3, 0.9 Hz, 1H, H_c), 6.53 (t, J = 2.6, 1.8 Hz, 1H, H_h), 2.10 (s, 6H, H_n).

^{13}C NMR (126 MHz, Acetone-d_6, 298 K) δ = 170.49 (C_l), 152.61 (C_d), 141.98 ($C_{g,f}$), 131.28 (C_b), 128.08 (C_i), 119.57 (C_c), 116.66 (C_a), 112.77 (C_e), 108.78 (C_h), 56.93 (C_m), 30.72 (C_n).

HRMS-APCI(+): 309.0243 [M+H]$^+$, calculated for $C_{13}H_{14}BrN_2O_2^+$: 309.0233.

3.6 Synthesis of Control Compound S16

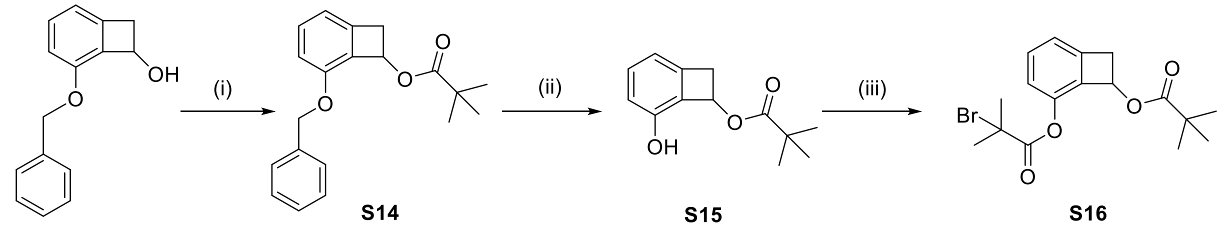

Figure S6. Synthetic route to **S16**. Conditions: (i) Trimethylacetyl chloride, Et_3N, DCM, rt, 24 h, 65% yield. (ii) H_2 (gas), Pd/C powder, Methanol, EtOAc, rt, 24h, 39% yield; (iii) BiBB, Et_3N, THF, rt, 5 h, 67% yield.

3.6.1 Synthesis of S14

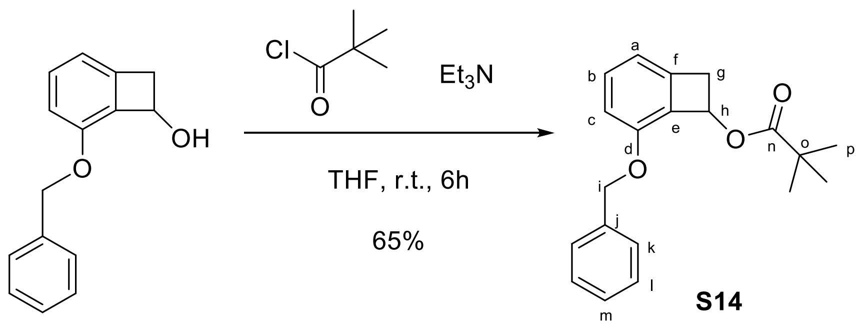

5 (50 mg, 0.22 mmol, 1.0 eq.), Trimethylacetyl chloride (46.4 µL, 0.44 mmol, 2.0 eq.), Et_3N (61.6 µL, 0.44 mmol, 2.0 eq.) were added into DCM (1.5 mL). The resulting mixture was stirred for 24h. The resulting mixture was then washed by water (5 mL) and extracted by DCM (10 mL) three times, then dried by $MgSO_4$. The crude was purified by PTLC (silica, PE: EtOAc 20:1) to get **S14** as white powder (44.5 mg, 0.143 mmol, 65% yield).

^{1}H NMR (400 MHz, Acetone-d_6, 298 K) δ = 7.47 – 7.36 (m, 4H, $H_{k,l}$), 7.36 – 7.27 (m, 2H, $H_{b,m}$), 6.83 (dd, , *J* = 8.5, 0.8 Hz, 1H, H_c), 6.77 (dd, *J* = 7.2, 0.8 Hz, 1H, H_a), 6.01 (dd, *J* = 4.4, 1.8 Hz, 1H, H_h), 5.24 (s, 2H, H_i), 3.61 (ddt, *J* = 14.6, 4.4, 0.8 Hz, 1H, H_g), 3.00 (ddt, *J* = 14.6, 1.8, 0.9 Hz, 1H, H_g), 1.23 (s, 9H, H_p).

^{13}C NMR (101 MHz, Acetone-d_6, 298 K) δ = 177.97 (C_n), 154.71 (C_d), 145.54 (C_f), 138.41 (C_j), 132.83 (C_b), 129.44 (C_k), 128.82 (C_m), 128.18 (C_l), 127.68 (C_e), 116.57 (C_a), 115.92 (C_c), 71.87 (C_h), 71.64 (C_i), 40.80 (C_g), 39.28 (C_o), 27.48 (C_p).

HRMS-ESI(+): 333.1459 $[M+Na]^+$, calculated for $C_{20}H_{22}O_3Na^+$: 333.1461.

3.6.2 *Synthesis of S15*

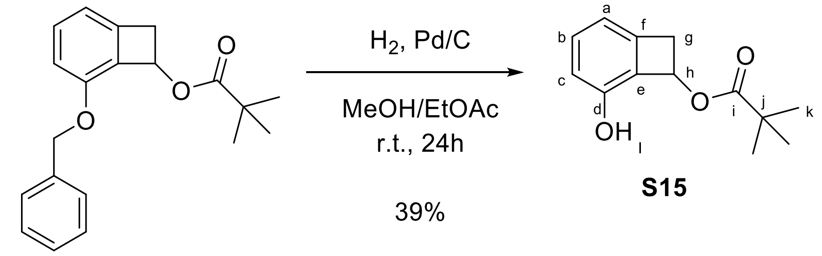

S14 (40 mg, 0.13 mmol, 1.0 eq.), Pd/C powder (14 mg), methanol (3 mL) and EtOAc (3 mL) were added together in a dried flask purged with nitrogen. Then the system was purged with hydrogen and the reaction was kept under a hydrogen atmosphere for 24 h. The mixture was filtrated, and the residual solid was washed with methanol. The organic fractions were combined, concentrated to dryness, and purified by PTLC (silica, PE:EtOAc 20:1) to give **S15** as colourless solid (11.2 Mg, 0.051 mmol, 39% yield).

^{1}H NMR (400 MHz, Acetone-d_6, 298 K) δ = 8.13 (s, 1H, H_l), 7.24 (t, J = 7.2 Hz, 1H, H_b), 6.70 – 6.62 (m, 2H, $H_{a,c}$), 5.65 (dd, J = 4.6, 1.9 Hz, 1H, H_h), 3.56 (ddt, J = 14.3, 4.5, 0.7 Hz, 1H, H_g), 3.20 (ddt, J = 14.3, 1.8, 0.9 Hz, 1H, H_g), 1.23 (s, 9H, H_k),.

^{13}C NMR (101 MHz, Acetone-d_6, 298 K) δ = 181.98 (C_i), 152.40 (C_d), 144.85 (C_f), 133.45 (C_b), 128.71 (C_e), 115.30 (C_a), 114.97 (C_c), 71.75 (C_h), 39.63 (C_j), 38.31 (C_g), 27.28 (C_k).

HRMS-ESI(+): 243.0997 $[M+Na]^+$, calculated for $C_{13}H_{16}O_3Na^+$: 243.1099.

3.6.3 *Synthesis of S16*

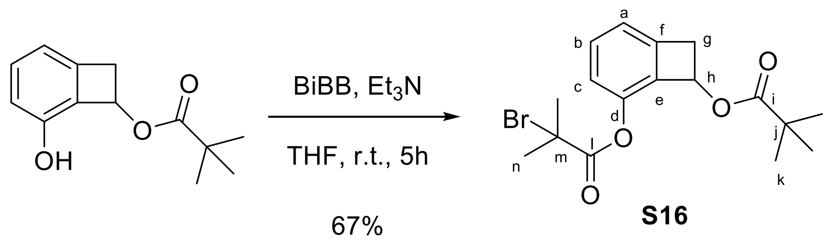

S15 (10 mg, 0.045 mmol, 1.0 eq.), BiBB (11.2 µL, 0.09 mmol, 2.0 eq.), Et_3N (12.7 µL, 0.09 mmol, 2.0 eq.) were added into THF (1 mL) and the resulting mixture was stirred for 5h. The resulting mixture was then washed by water (5 mL) and extracted by DCM (5 mL) three times, then dried by $MgSO_4$. The crude was purified by PTLC (silica, PE: EtOAc 30:1) to get **S16** as colourless solid (11.2 mg, 0.03mmol, 67% yield).

^{1}H NMR (500 MHz, Acetone-d_6, 298 K) δ = 7.46 (t, *J* = 7.8 Hz, 1H, H_b), 7.15 (d, *J* = 7.2 Hz, 1H, H_a), 7.02 (d, *J* = 8.3 Hz, 1H, H_c), 5.88 (dd, *J* = 4.6, 2.0 Hz, 1H, H_h), 3.67 (dd, *J* = 14.6, 4.6 Hz, 1H, H_g), 3.18 (dd, *J* = 14.7, 1.9 Hz, 1H, H_g), 2.09 (s, 3H, H_n), 2.05 (s, 3H, H_n, overlapped with the solvent peak), 1.19 (s, 6H, H_k).

^{13}C NMR (126 MHz, Acetone-d_6, 298 K) δ = 178.23 (C_i), 169.68 (C_l), 145.81 (C_d), 145.14 (C_f), 135.81 (C_e), 132.72 (C_b), 122.42 (C_a), 120.27 (C_c), 71.32 (C_h), 56.94 (C_m), 39.61 (C_g), 39.20 (C_j), 30.89 (C_n), 30.86 (C_n), 27.40 (C_k).

HRMS -ESI(+): 391.0498 [M+Na]$^+$, calculated for $C_{17}H_{21}BrO_4Na^+$: 391.0515.

4. Synthesis of Polymers

4.1 Representative Procedure for SET-LRP of Methyl Acrylate Using Mechanophore Initiators

Methyl acrylate was filtered through basic alumina to remove the inhibitor prior to use. A stock catalytic solution of Me_6TREN (16 µL, 0.060 mmol) and $CuBr_2$ (5.6 mg, 0.025 mmol) in dry DMSO (1 mL) was prepared. The appropriate initiator compound along with catalytic solution, methyl acylate and dry DMSO was added into a 5 mL microwave vial. This solution was degassed by bubbling with nitrogen gas for 10 mins. A Cu(0) wire wrapped around a stirrer bar, having been cleaned in 12 N HCl for 20 mins, washed by excess acetone and dried, was added to the reaction mixture. The reaction mixture was degassed for a further 2 mins before being allowed to stir for 20 - 45 min (depends on the extent of polymerization, as determined approximately by the increasing viscosity of the solution). The reaction solution was added dropwise to a solution of vigorously stirred methanol; the precipitated polymer was recovered and dried under vacuum for 24 h to yield a white material. Molecular weight and polydispersity indices were determined using an analytical SEC that had been calibrated with polystyrene standards.

4.2 Synthesis of Polymer 1

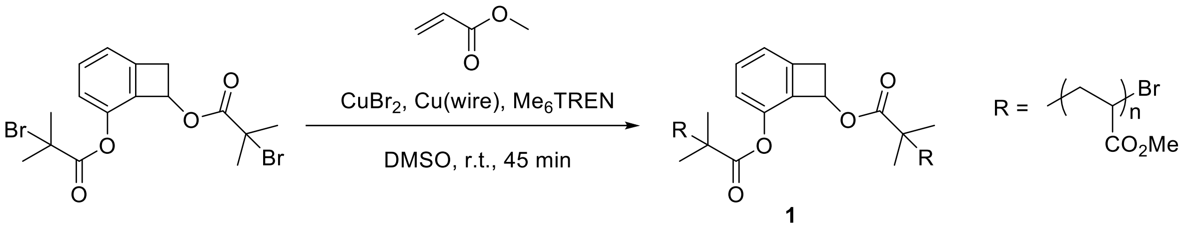

Synthesis followed the representative procedure. **7** (2 mg, 4.6 µmol, 1.0 eq.), 20 µL of catalytic solution (Me_6TREN: 2.3 µmol, 0.5 eq.; $CuBr_2$: 0.92 µmol, 0.2 eq.), methyl acylate (498 µL, 5.53 mmol, 1200.0 eq.), Cu (0) wire (~3 cm, ~30 mg, 0.5 mmol, ~500.0 eq.) and dry DMSO (500 µL) were used in the reaction to yield polymer **1** (526 mg, M_n = 116 kDa; $Đ$ = 1.09).

4.3 Synthesis of Reference Polymer 8_{ref}

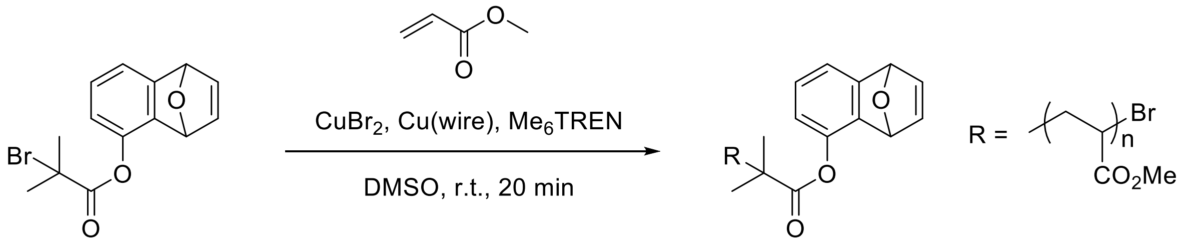

8ref

Synthesis followed the representative procedure. **S5** (2 mg, 6.5 µmol, 1.0 eq.), 20 µL of catalytic solution (Me_6TREN: 3.23 µmol, 0.5 eq.; $CuBr_2$: 1.29 µmol, 0.2 eq.), methyl acylate (699 µL, 7.76 mmol, 1200.0 eq.), Cu (0) wire (~3 cm, ~30 mg, 0.5 mmol, ~500.0 eq.) and dry DMSO (700 µL) were used in the reaction to yield polymer $\mathbf{8_{ref}}$ (77 mg, M_n = 50 kDa; $Đ$ = 1.58).

4.4 Synthesis of Reference Polymer S17

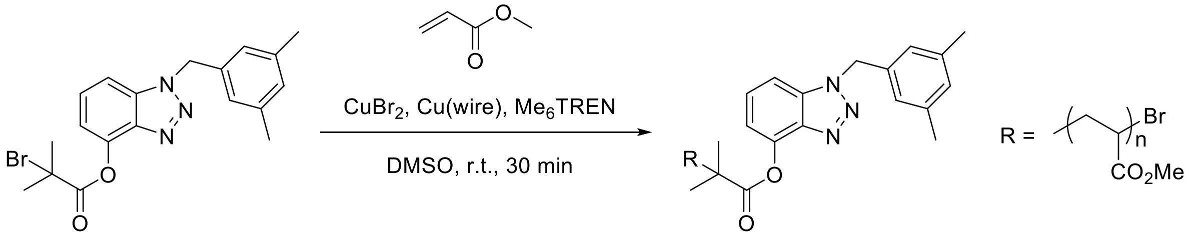

S17

Synthesis followed the representative procedure. **S8** (2 mg, 5.0 µmol, 1.0 eq.), 20 µL of catalytic solution (Me_6TREN: 2.5 µmol, 0.5 eq.; $CuBr_2$: 1.0 µmol, 0.2 eq.), methyl acylate (516 µL, 5.99 mmol, 1200.0 eq.), Cu (0) wire (~3 cm, ~30 mg, 0.5 mmol, ~500.0 eq.) and dry DMSO (500 µL) were used in the reaction to yield polymer **S17** (143 mg, M_n = 93 kDa; $Đ$ = 1.46).

4.5 Synthesis of Reference Polymer S18

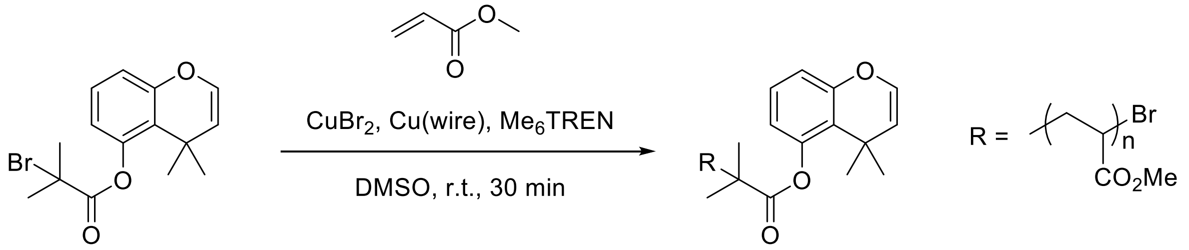

S18

Synthesis followed the representative procedure. **S10** (2 mg, 6.2 µmol, 1.0 eq.), 20 µL of catalytic solution (Me_6TREN: 3.1 µmol, 0.5 eq.; $CuBr_2$: 1.24 µmol, 0.2 eq.), methyl acylate (637 µL, 7.41 mmol, 1200.0 eq.), Cu (0) wire (~3 cm, ~30 mg, 0.5 mmol, ~500.0 eq.) and dry DMSO (700 µL) were used in the reaction to yield polymer **S18** (115 mg, M_n = 102 kDa; $Đ$ = 1.26).

4.6 Synthesis of Reference Polymer S19

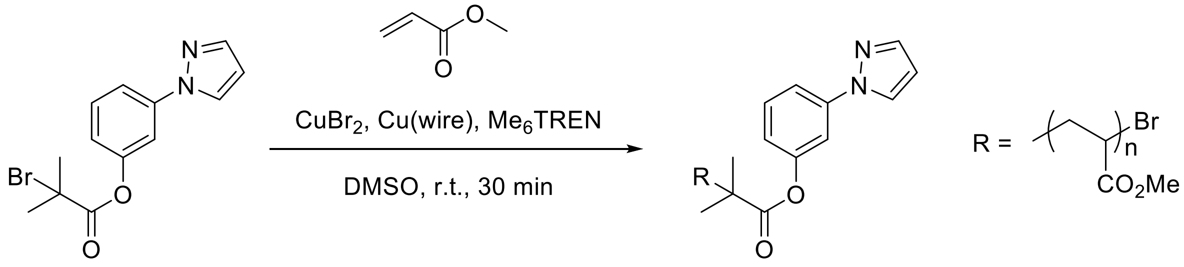

S19

Synthesis followed the representative procedure. **S12** (2 mg, 6.5 µmol, 1.0 eq.), 20 µL of catalytic solution (Me$_6$TREN: 3.25 µmol, 0.5 eq.; $CuBr_2$: 1.30 µmol, 0.2 eq.), methyl acylate (670 µL, 7.79 mmol, 1200.0 eq.), Cu (0) wire (~3 cm, ~30 mg, 0.5 mmol, ~500.0 eq.) and dry DMSO (500 µL) were used in the reaction to yield polymer **S19** (73 mg, M_n = 45 kDa; *Đ* = 1.21).

4.7 Synthesis of Control Polymer S20

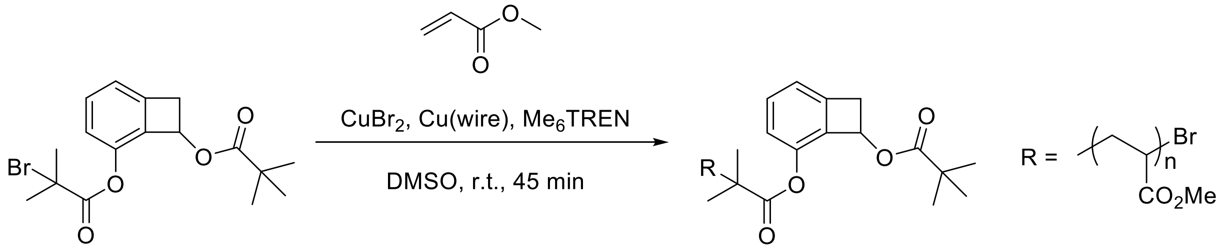

S20

Synthesis followed the representative procedure. **S16** (2 mg, 5.3 µmol, 1.0 eq.), 20 µL of catalytic solution (Me$_6$TREN: 2.67 µmol, 0.5 eq.; $CuBr_2$: 1.07 µmol, 0.2 eq.), methyl acylate (587 µL, 6.52 mmol, 1200.0 eq.), Cu (0) wire (~3 cm, ~30 mg, 0.5 mmol, ~500.0 eq.) and dry DMSO (600 µL) were used in the reaction to yield polymer **S20** (244 mg, M_n = 89 kDa; *Đ* = 1.53).

4.8 SEC Data for Synthesised Polymers

Table S1. M_n and *Đ* values for all synthesised polymers.

Polymer	M_n / kDa	*Đ*
**1**	116	1.09
**8$_{ref}$**	50	1.58
**S17**	93	1.46
**S18**	102	1.26
**S19**	45	1.21
**S20**	89	1.53

4.9 SEC Traces for Polymers with Mechanophore

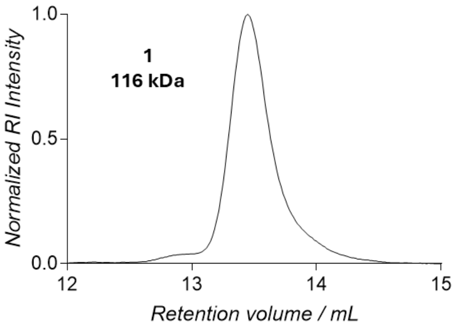

Figure S7. SEC traces for polymer **1**

4.10 SEC Traces for Reference Polymers and Control Polymer

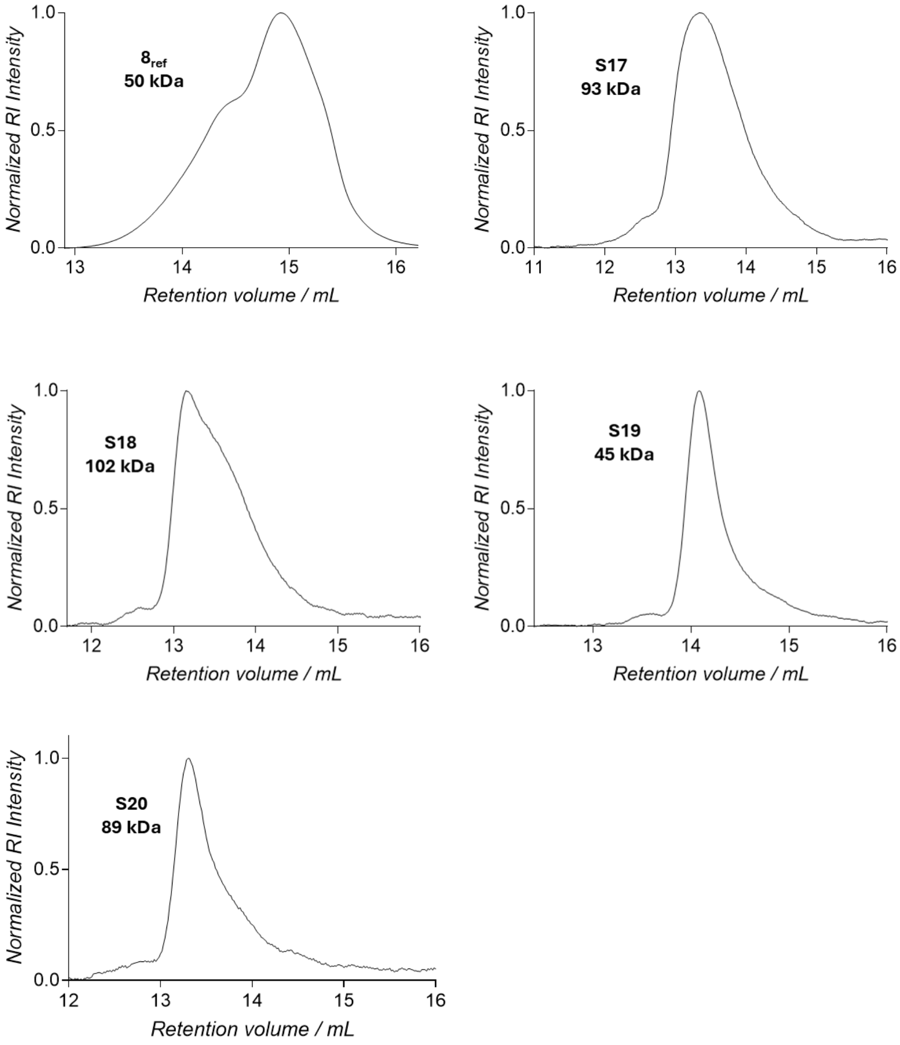

Figure S8. SEC traces of reference polymers **8_{ref}, S17, S18, S19**, and control polymer **S20**).

5. Mechanophore Activation via Ultrasound

5.1 General Procedure for Sonication Experiments

The appropriate polymer (20 mg) was added to a Suslick cell and dissolved in dry MeCN (16 mL). The solution was degassed by bubbling N_2 through it for a minimum of 10 min prior to the start of sonication; bubbling of N_2 was also maintained throughout the experiment. Trapping reagents (0.35 mmol, 2000.0 eq) for each experiment have been added just before the sonication. The Suslick cell was cooled with an ice bath throughout the duration of the sonication to maintain a temperature of ~ 5-10 °C inside the cell. Pulsed ultrasound was applied to the system (1 s ON / 1 s OFF, 25% amplitude (13.0 $W.cm^{-2}$), 20 kHz) for the desired period of time. After sonication, the solvent was evaporated, and the polymer was analysed by SEC and NMR spectroscopy. The post-sonication polymer was recovered and washed with MeOH to extract any small molecules not attached to polymer chains. The remaining MeOH-washed polymer and the concentrated MeOH washings were then analysed by NMR spectroscopy.

5.2 Sonication of Polymer 1 with Furan

Mechanophore activation rate calculated from integration of cyclo-furan trapped reference peaks (integration of peak z (or w) / integration of peak e) is 46%; Mechanophore activation rate calculated from integration of vinyl pivalate reference peaks (integration of peak 2_{tr} (or 2_{ci}) / integration of peak e) is 52%; The trapping rate of furan is 88%.

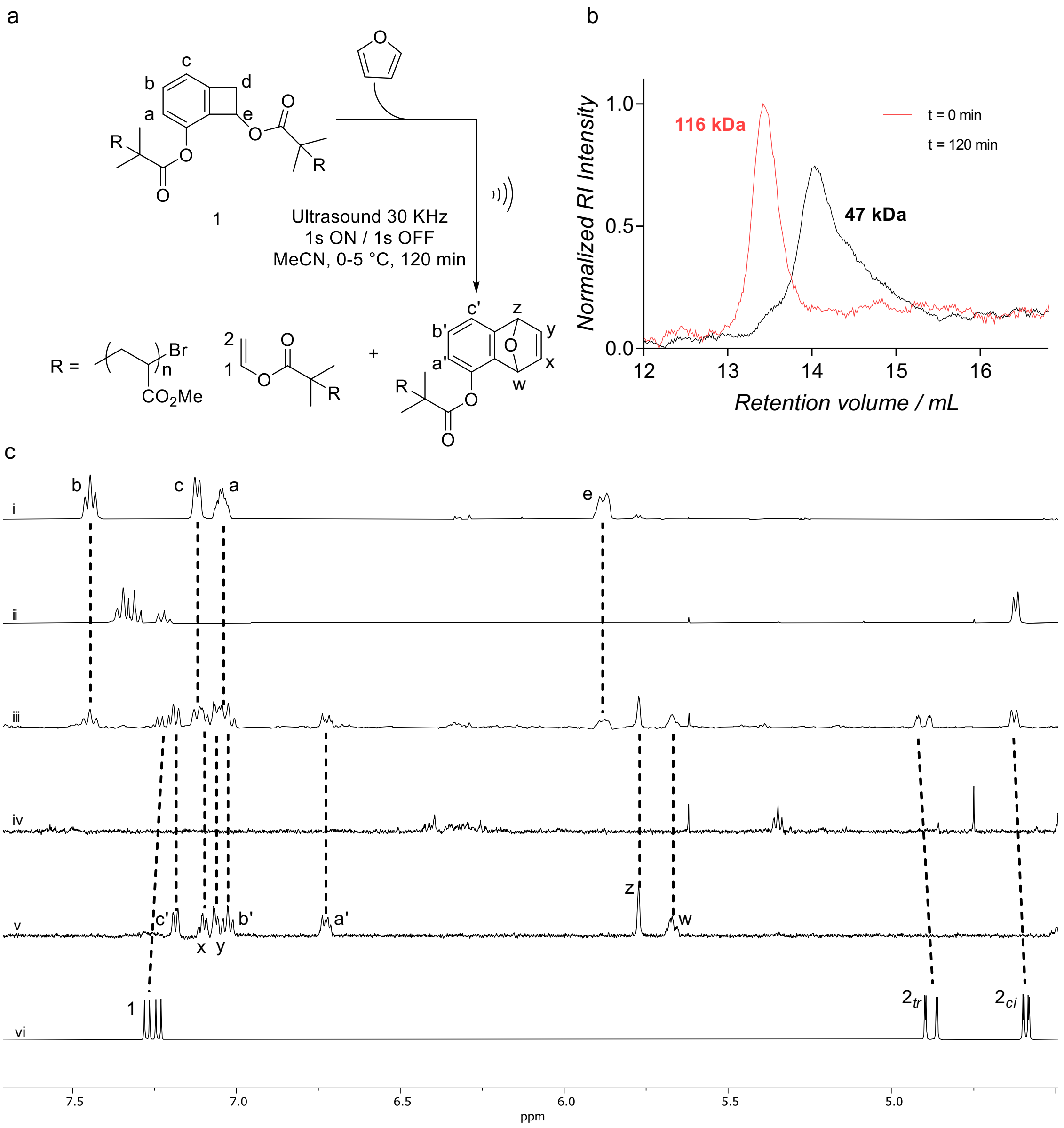

Figure S9. Sonication of polymer **1**. (a) Ultrasonication mechanism of polymer **1**. (b) SEC traces of polymer **1**. (c) Partial ^{1}H NMR (400 MHz, Acetone-d_6, 298K) spectra comparison of the pre-sonication polymer **1** (i), post-sonication polymer **1** before being washed with methanol (ii), post-sonication polymer **1** after being washed with methanol (iii), concentrated methanol washings (iv), reference polymer $\mathbf{8_{ref}}$ (v), reference vinyl pivalate compound (vi).

5.3 Sonication of Polymer 1 with S7

Mechanophore activation rate calculated from integration of triazole trapped reference peaks (integration of peak a' (or b') / integration of peak b) is 39%; Mechanophore activation rate calculated from integration of vinyl pivalate reference peaks (integration of peak 2_{tr} (or 2_{ci}) / integration of peak b) is 45%; The trapping rate of **S7** is 87%.

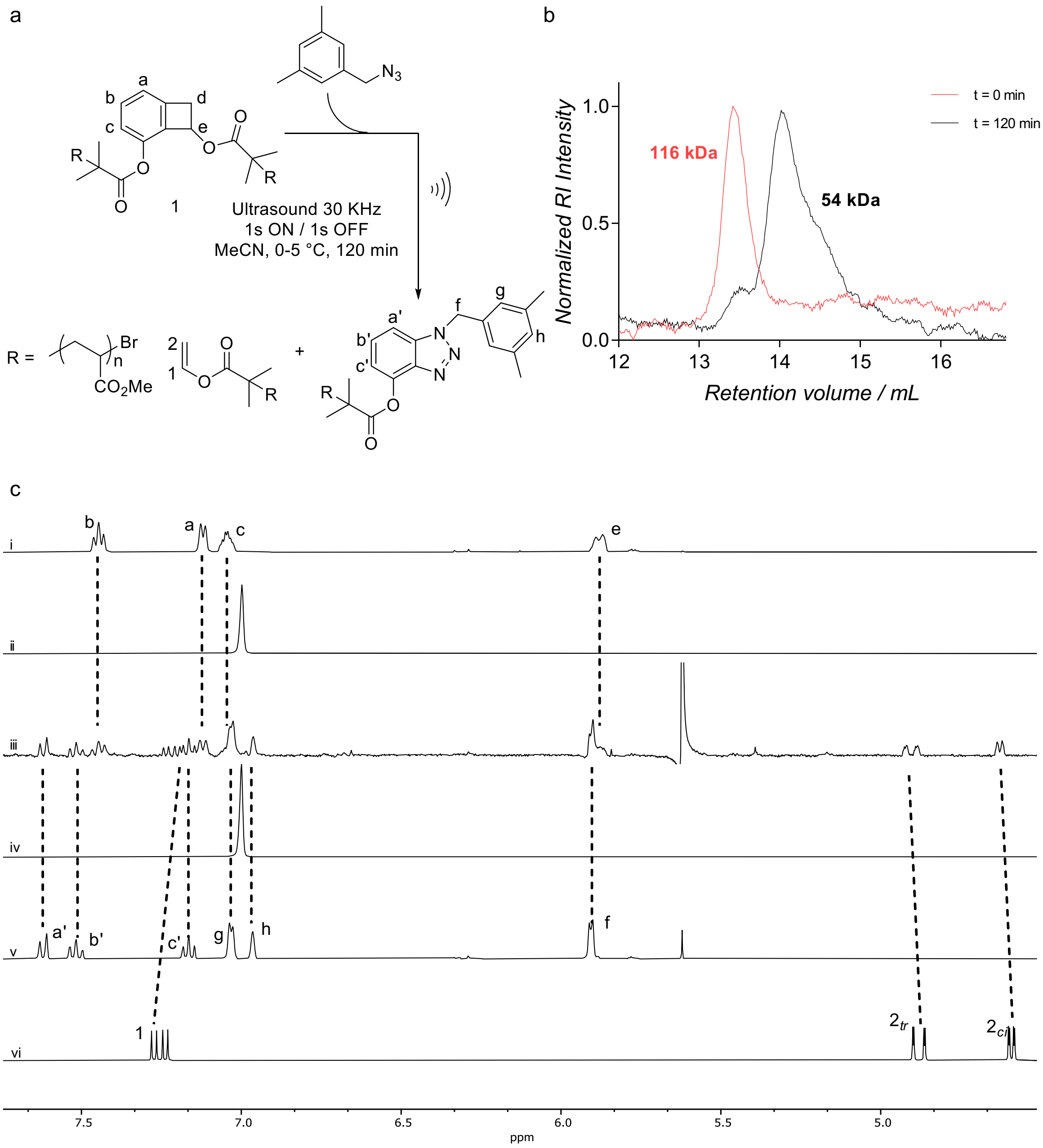

Figure S10. Sonication of polymer **1**. (a) Ultrasonication mechanism of polymer **1**. (b) SEC traces of polymer **1**. (c) Partial ^{1}H NMR (400 MHz, Acetone-d_6, 298K) spectra comparison of the pre-sonication polymer **1** (i), post-sonication polymer **1** before being washed with methanol (ii), post-sonication polymer **1** after being washed with methanol (iii), concentrated methanol washings (iv), reference polymer **S17** (v), reference vinyl pivalate compound (vi).

5.4 Sonication of Polymer 1 with 3-Methyl-2-butenal

Mechanophore activation rate calculated from integration of chromene trapped reference peaks (integration of peak f (or g) / integration of peak e) is 51%; Mechanophore activation rate calculated from integration of vinyl pivalate reference peaks (integration of peak 2_{tr} (or 2_{ci}) / integration of peak e) is 52%; The trapping rate of 3-Methyl-2-butenal is 98%.

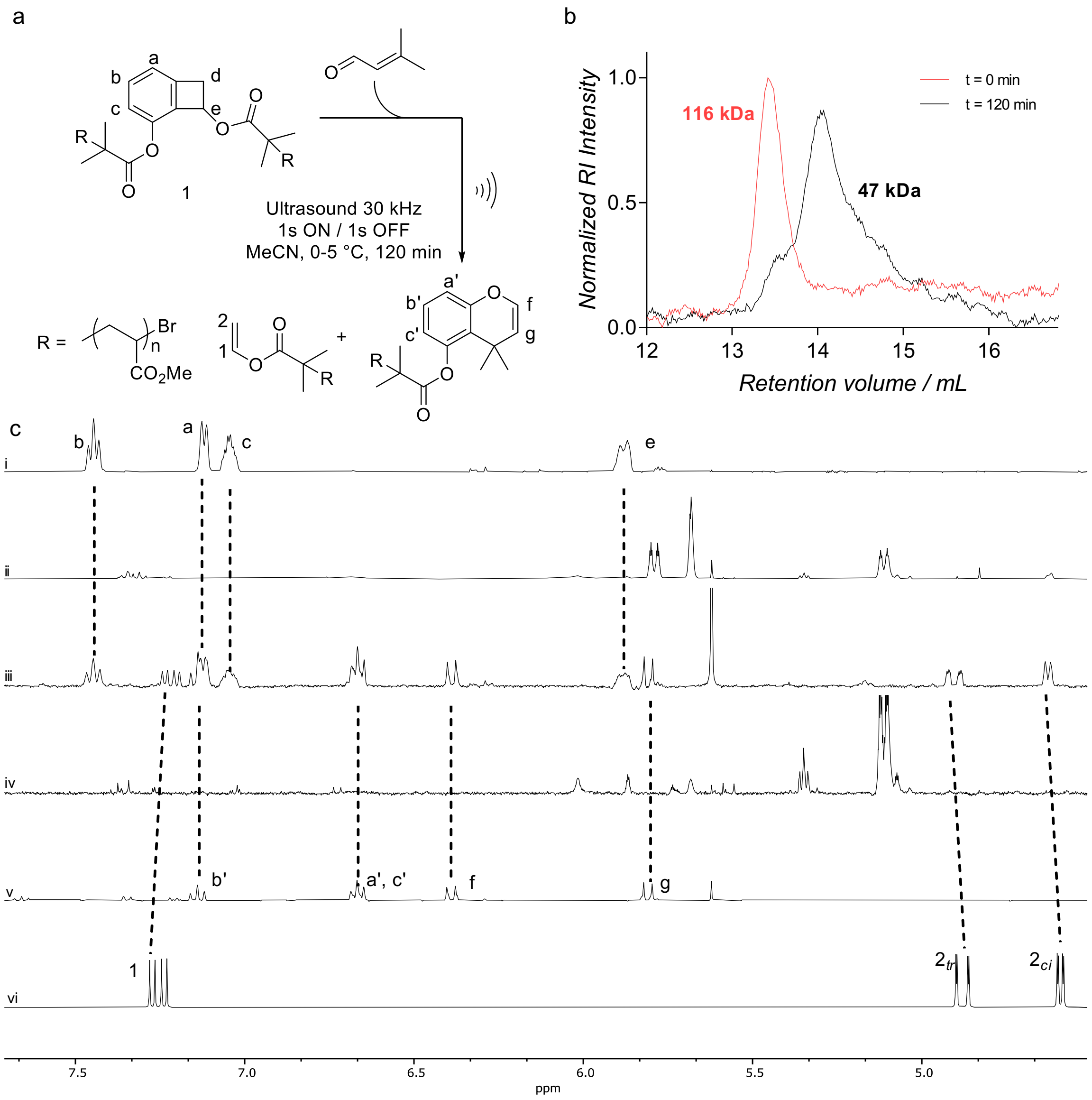

Figure S11. Sonication of polymer **1**. (a) Ultrasonication mechanism of polymer **1**. (b) SEC traces of polymer **1**. (c) Partial ^{1}H NMR (400 MHz, Acetone-d_6, 298K) spectra comparison of the pre-sonication polymer **1** (i), post-sonication polymer **1** before being washed with methanol (ii), post-sonication polymer **1** after being washed with methanol (iii), concentrated methanol washings (iv), reference polymer **S18** (v), reference vinyl pivalate compound (vi).

5.5 Sonication of Polymer 1 with 1H-Pyrazole

Mechanophore activation rate calculated from integration of pyrazole trapped reference peaks (integration of peak g (or h) / integration of peak e) is 45%; Mechanophore activation rate calculated from integration of vinyl pivalate reference peaks (integration of peak 2_{tr} (or 2_{ci}) / integration of peak e) is 52%; The trapping rate of 1H-Pyrazole is 87%.

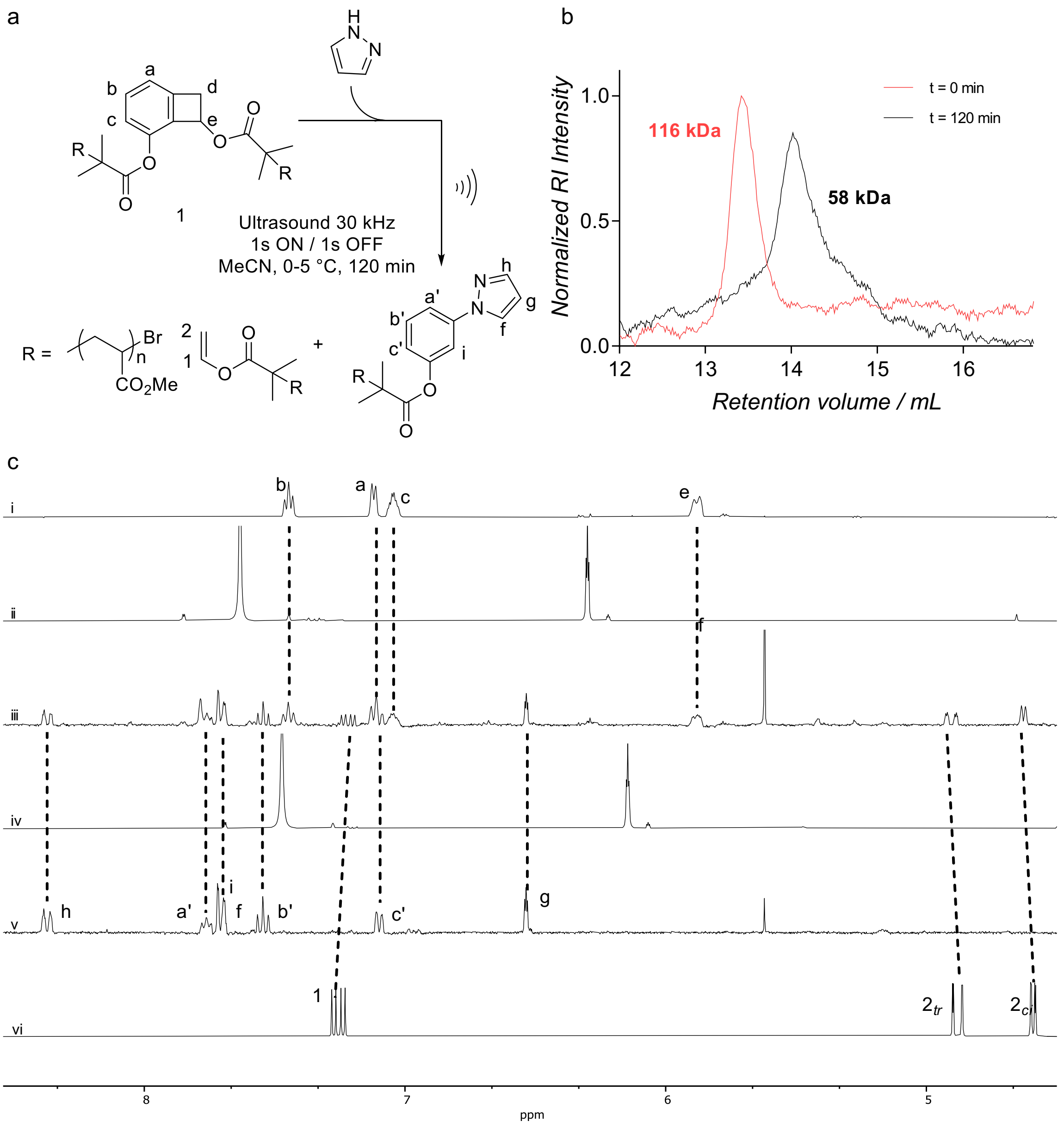

Figure S12. Sonication of polymer **1**. (a) Ultrasonication mechanism of polymer **1**. (b) SEC traces of polymer **1**. (c) Partial ^{1}H NMR (400 MHz, Acetone-d_6, 298K) spectra comparison of the pre-sonication polymer **1** (i), post-sonication polymer **1** before being washed with methanol (ii), post-sonication polymer **1** after being washed with methanol (iii), concentrated methanol washings (iv), reference polymer **S19** (v), reference vinyl pivalate compound (vi).

5.6 Sonication of Control Polymer S20

In order to prove that cleavage of the mechanophores was due to mechanical activation, and not other effects such as thermal activation, we subjected **S20**, a derivative of **5** positioned terminally in a chain of PMA, to the same sonication experiments used to cleave **1**, as described in Section 5. Analysis of the polymer post-sonication by ^{1}H NMR spectroscopy showed no change.

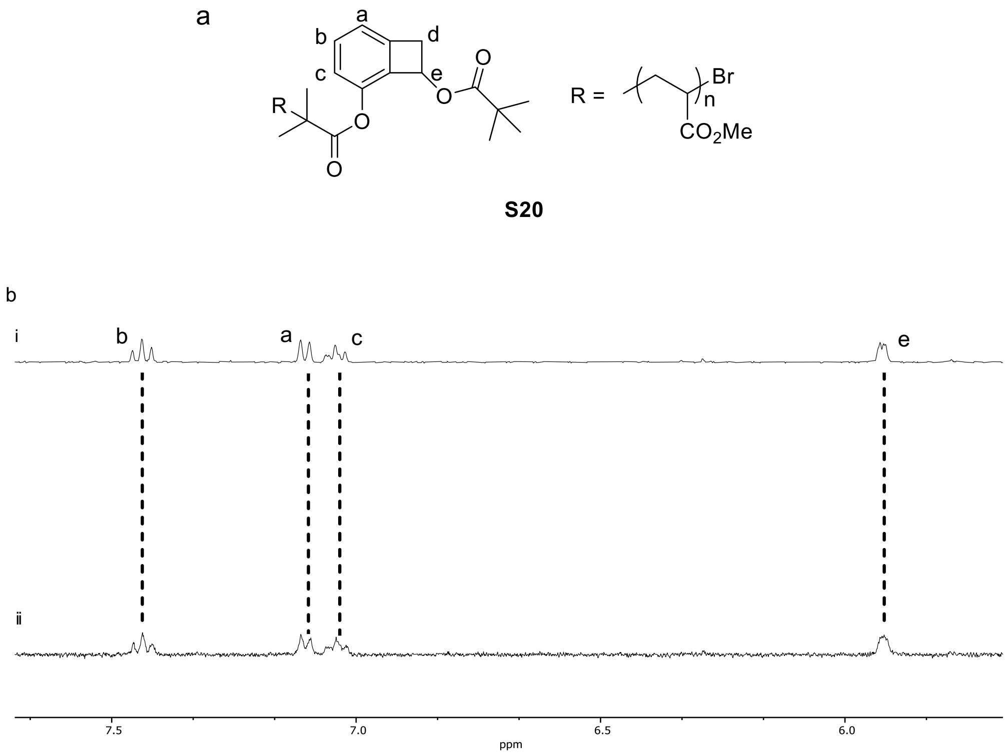

Figure S13. Sonication of polymer **S20**. (a) Structure of **S20**. (b) Partial ^{1}H NMR (400 MHz, Acetone-d_6, 298K) spectra comparison of the pre-sonication polymer **S20** (i), post-sonication polymer **S20** after being washed with methanol (ii).

6. NMR Spectra

6.1 Small Molecules NMR Spectra

6.1.1 Spectra of 5

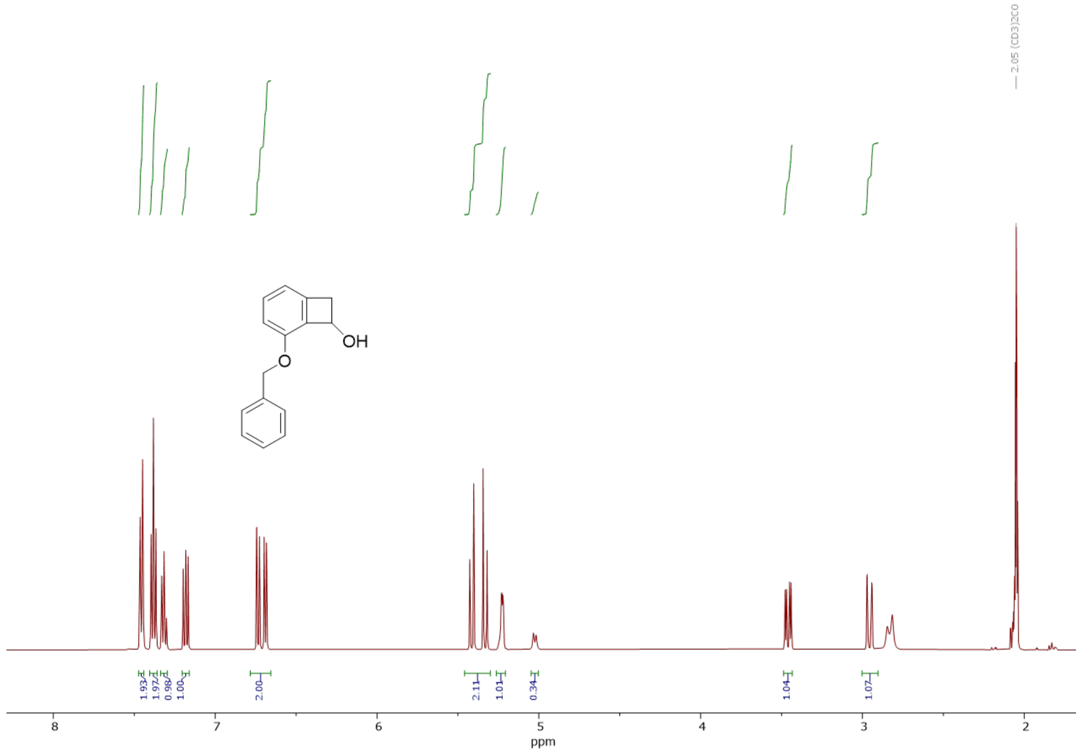

Spectrum S1. ^{1}H NMR (500 MHz, Acetone-d$_6$, 298 K) spectrum of compound **5**.

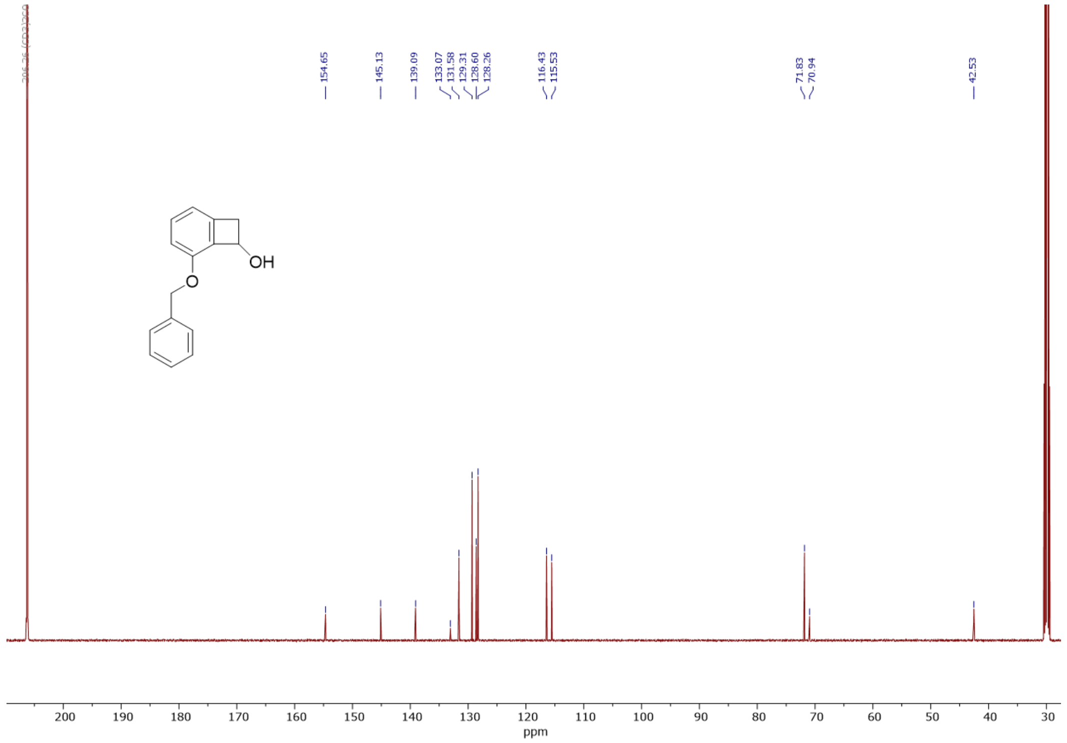

Spectrum S2. ^{13}C NMR (126 MHz, Acetone-d$_6$, 298 K) spectrum of compound **5**.

6.1.2 Spectra of S1

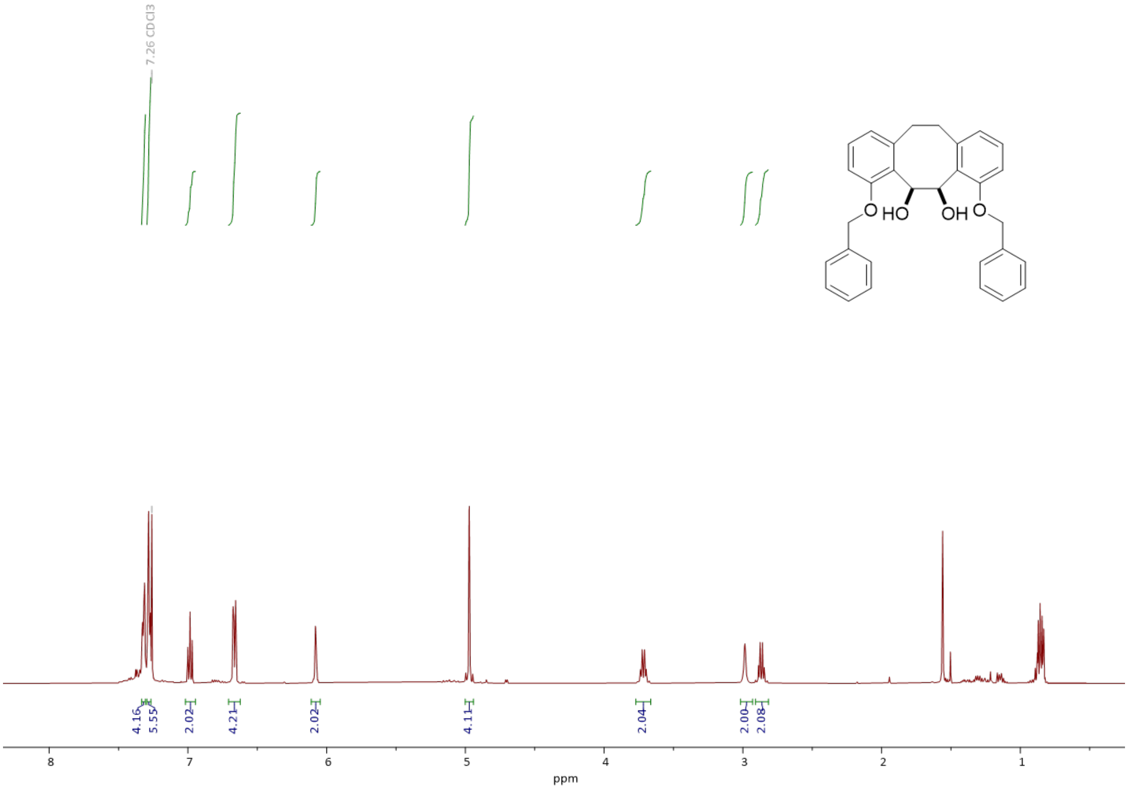

Spectrum S3. ^{1}H NMR (500 MHz, $CDCl_3$, 298 K) spectrum of compound **S1**.

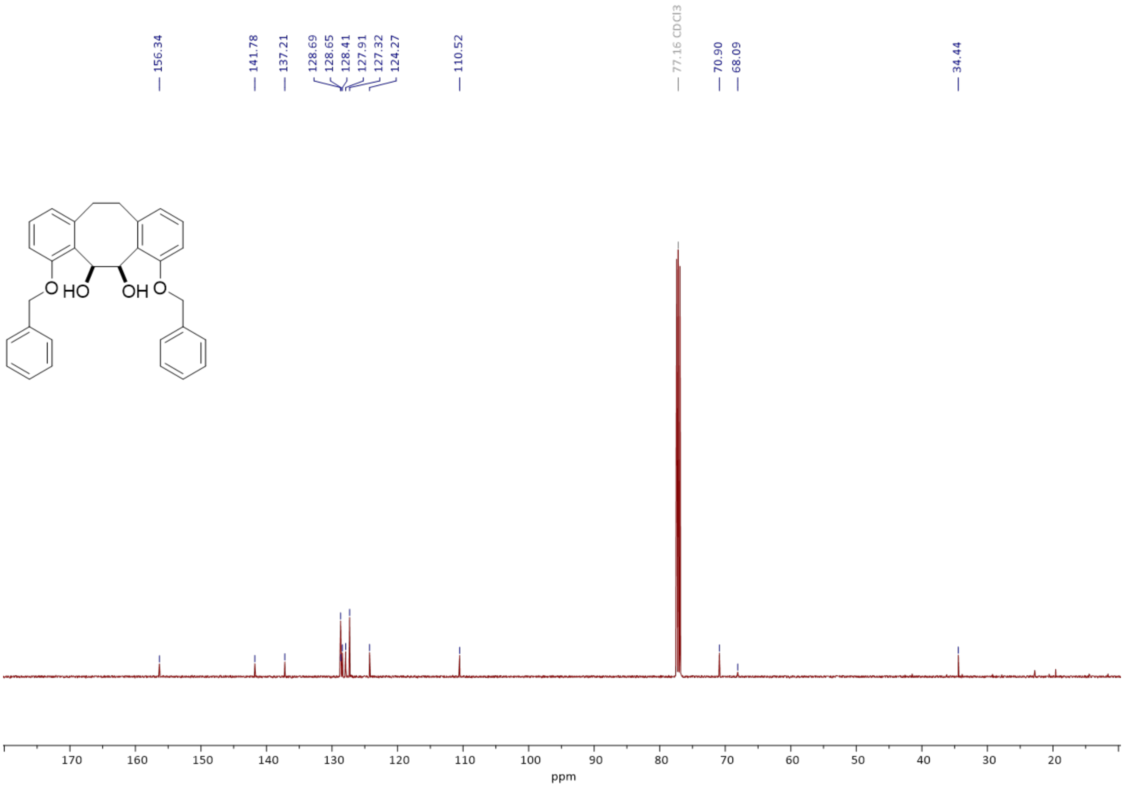

Spectrum S4. ^{13}C NMR (126 MHz, $CDCl_3$, 298 K) spectrum of compound **S1**.

6.1.3 Spectra of 6

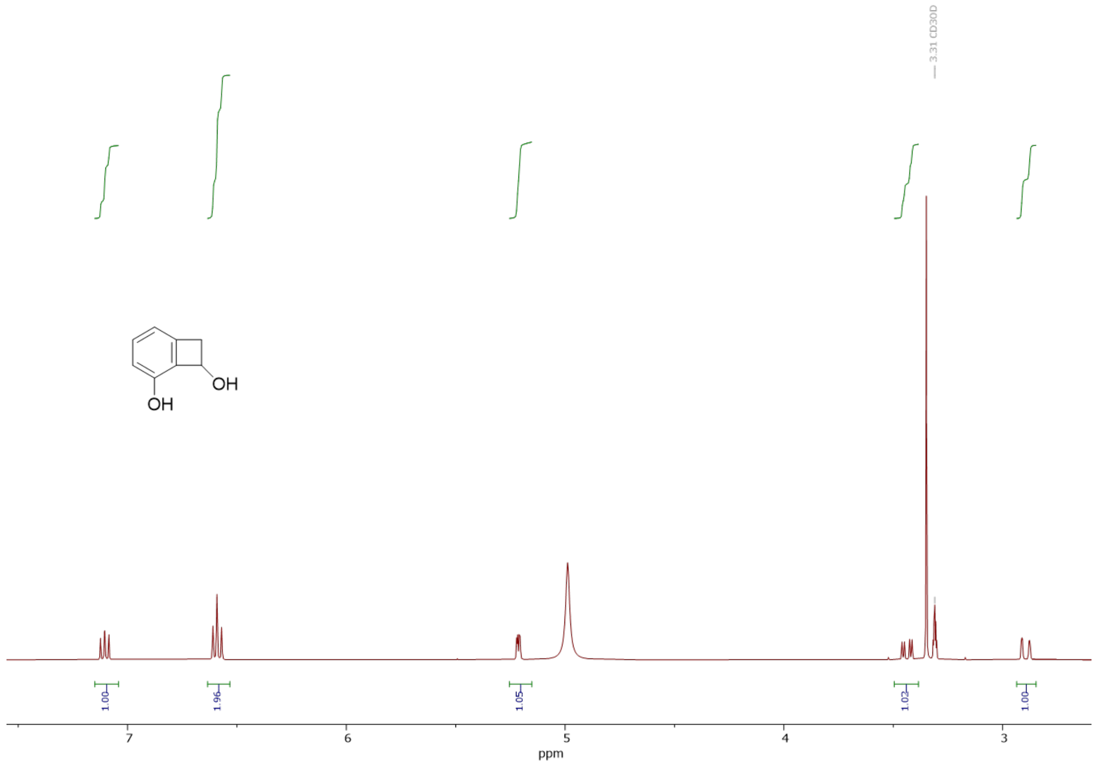

Spectrum S5. ^{1}H NMR (400 MHz, CD_3OD, 298 K) spectrum of compound **6**.

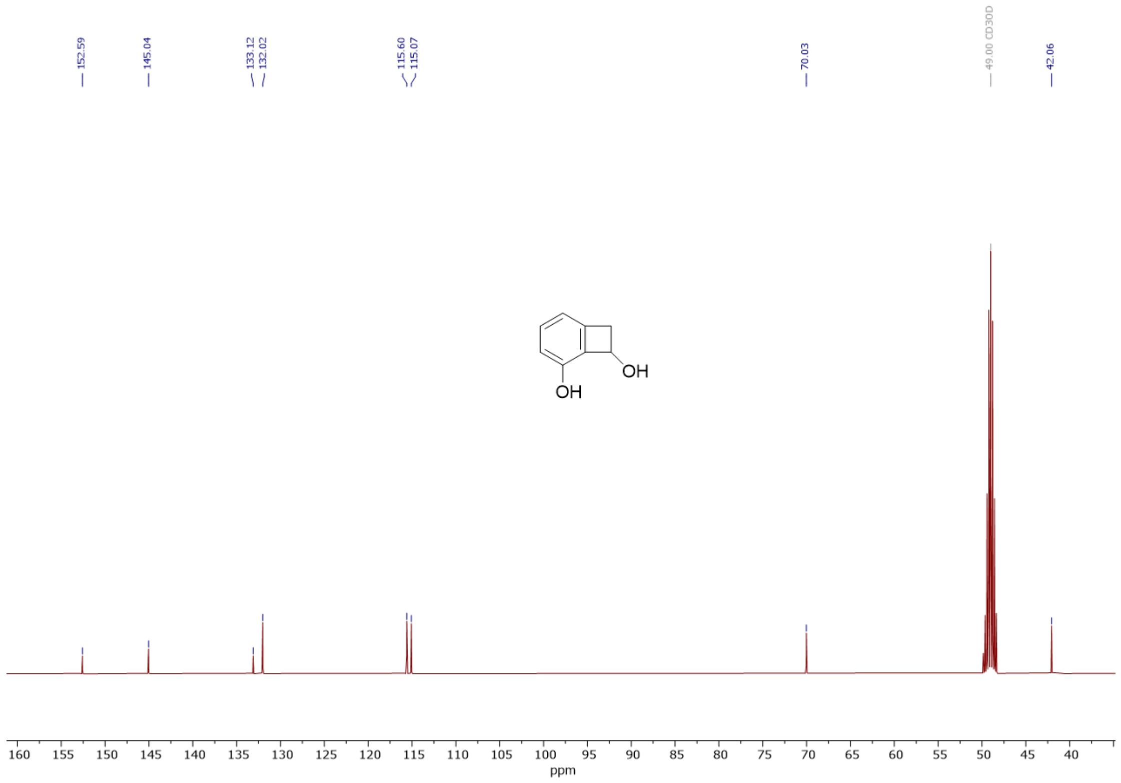

Spectrum S6. ^{13}C NMR (101 MHz, CD_3OD, 298 K) spectrum of compound **6**.

6.1.4 Spectra of 7

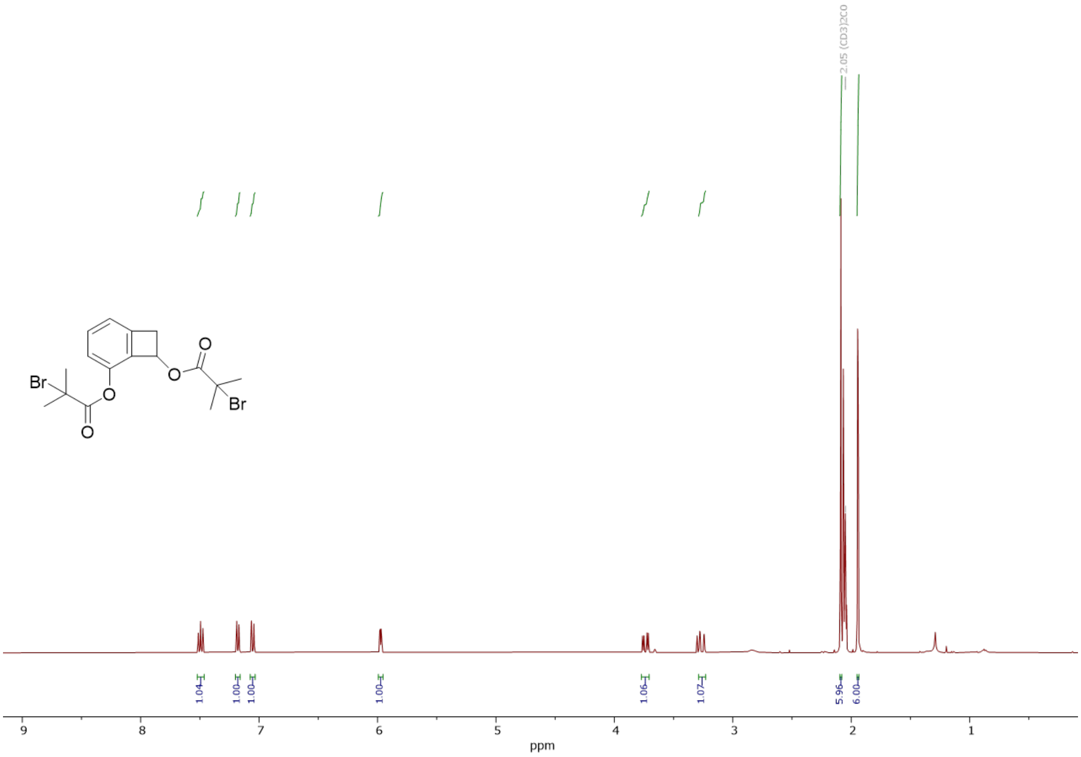

Spectrum S7. ^{1}H NMR (400 MHz, Acetone-d_6, 298 K) spectrum of compound **7**.

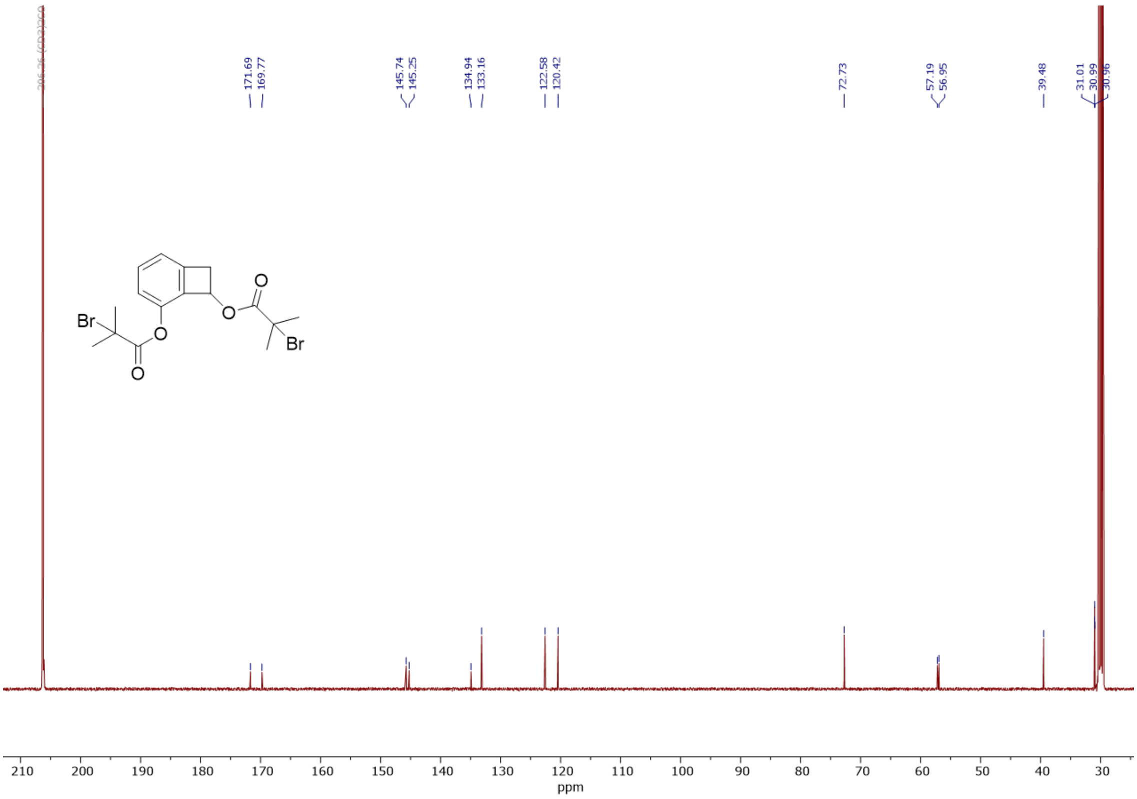

Spectrum S8. ^{13}C NMR (126 MHz, Acetone-d_6, 298 K) spectrum of compound **7**.

6.1.5 Spectrum of S2

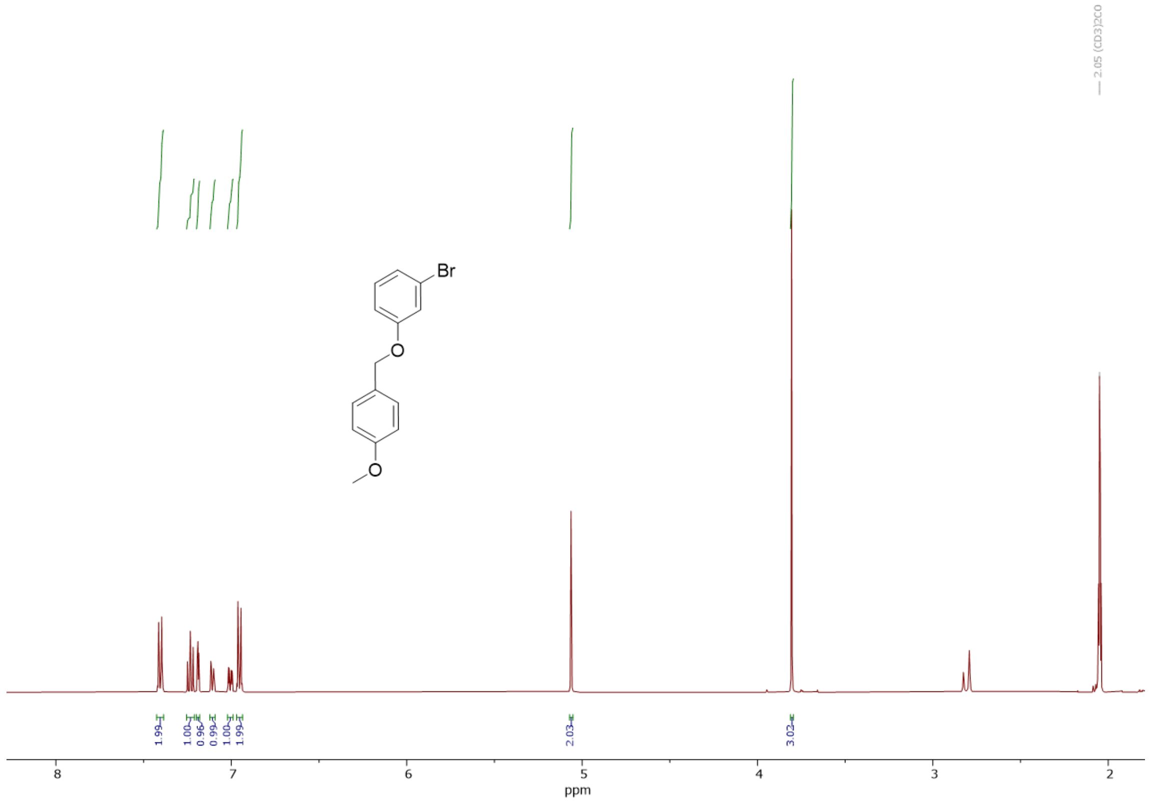

Spectrum S9. ^{1}H NMR (500 MHz, Acetone-d_6, 298 K) spectrum of compound **S2**.

6.1.6 Spectra of S3

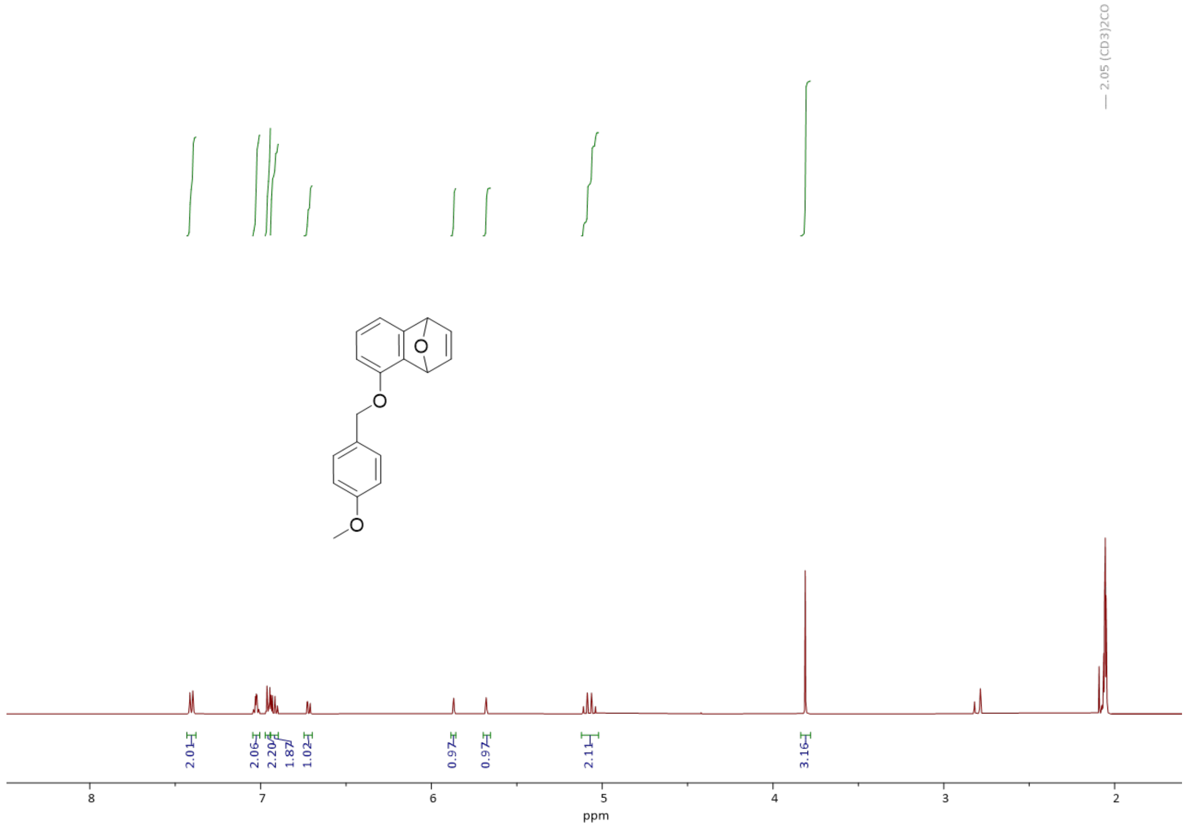

Spectrum S10. ^{1}H NMR (500 MHz, Acetone-d_6, 298 K) spectrum of compound **S3**

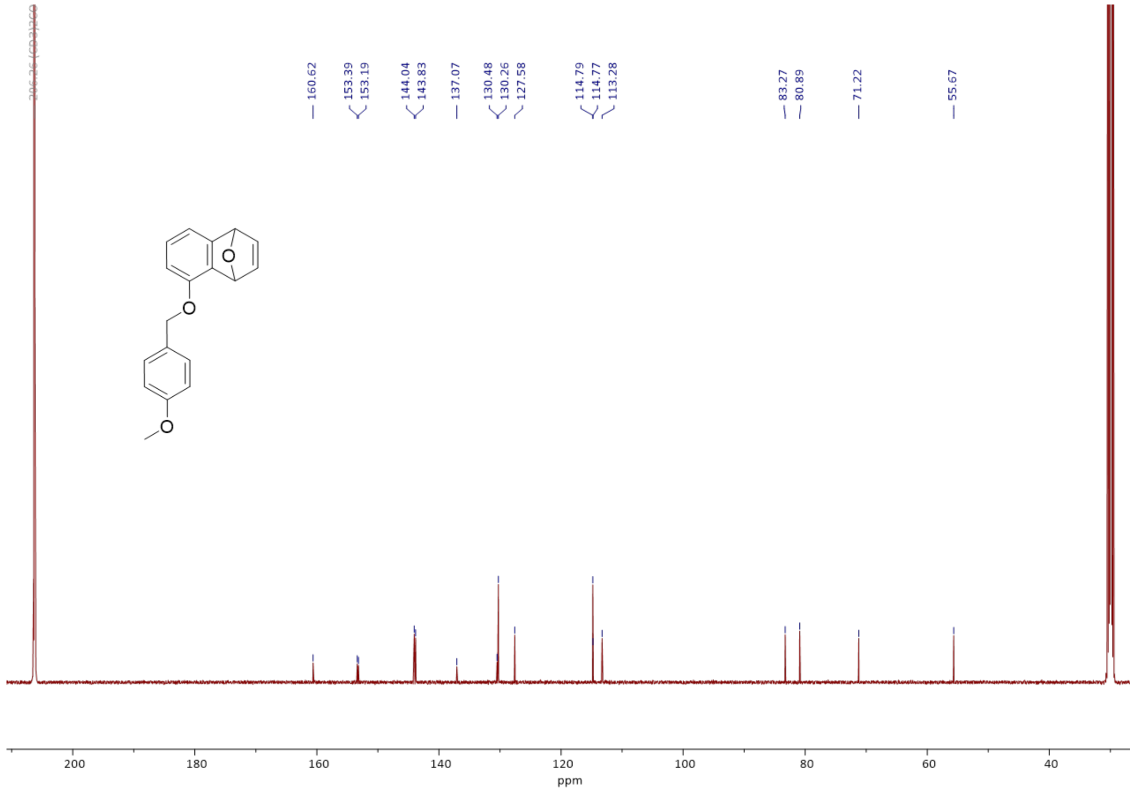

Spectrum S11. ^{13}C NMR (126 MHz, Acetone-d_6, 298 K) spectrum of compound **S3**.

6.1.7 Spectra of S4

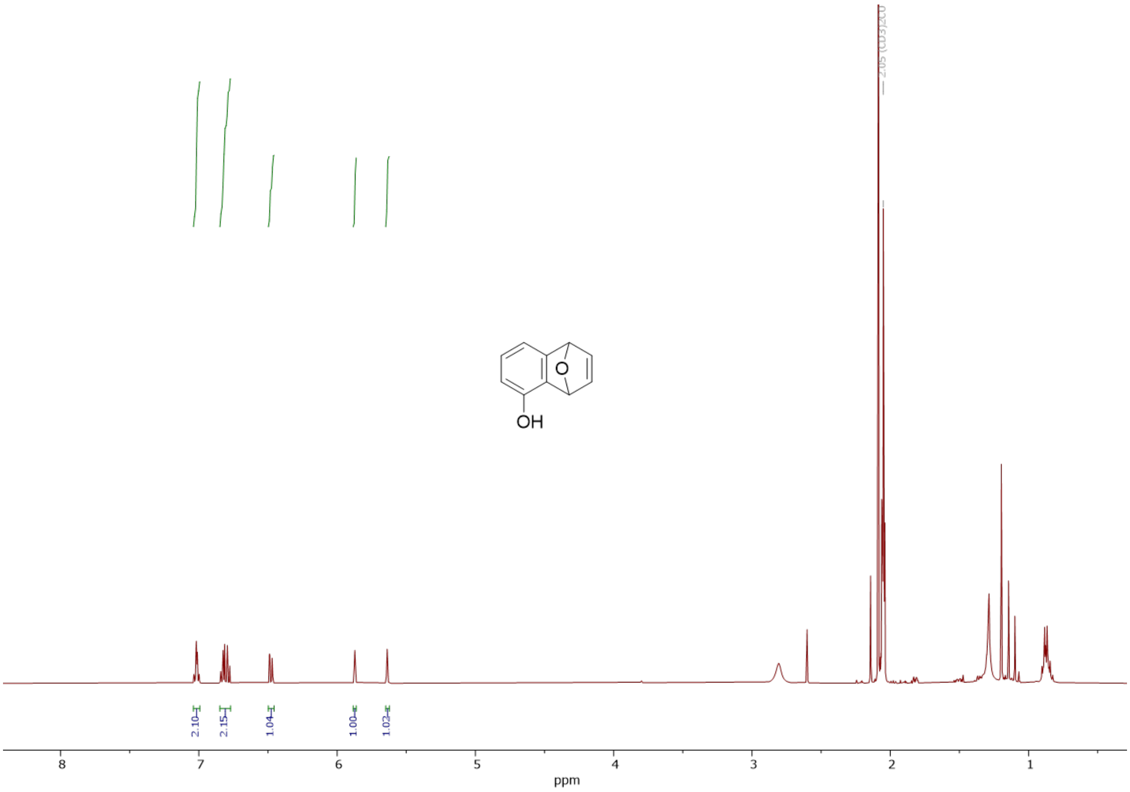

Spectrum S12. ^{1}H NMR (400 MHz, Acetone-d_6, 298 K) spectrum of compound **S4**.

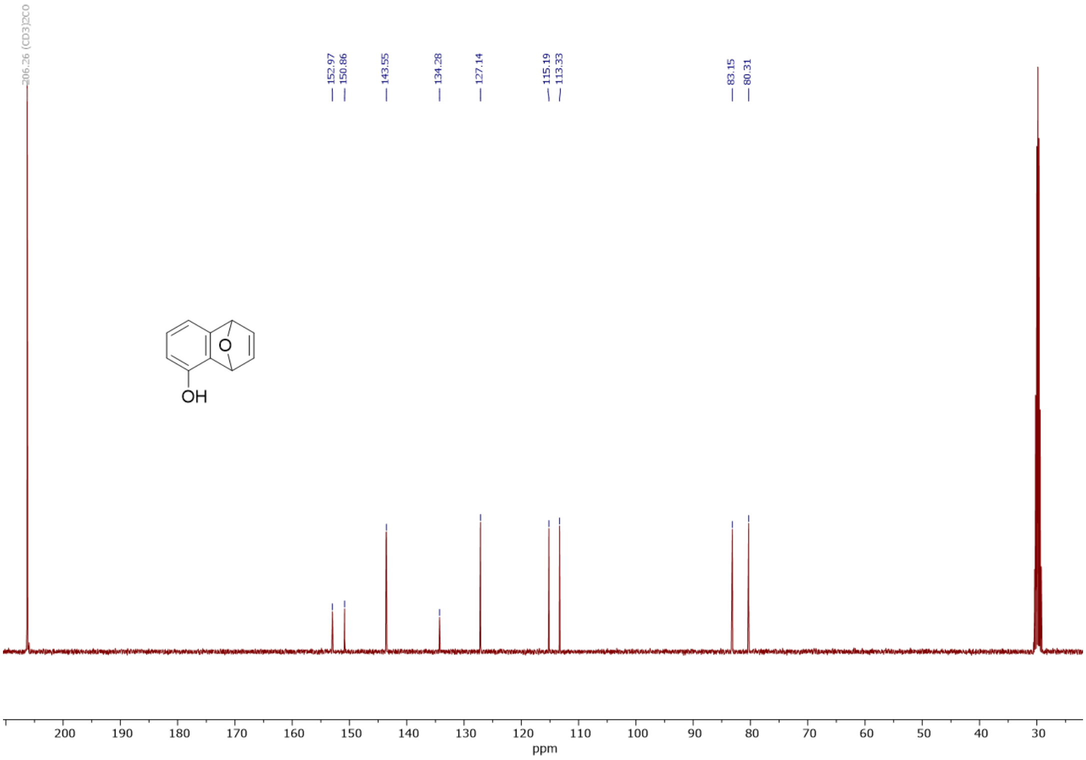

Spectrum S13. ^{13}C NMR (101 MHz, Acetone-d_6, 298 K) spectrum of compound **S4**.

6.1.8 Spectra of S5

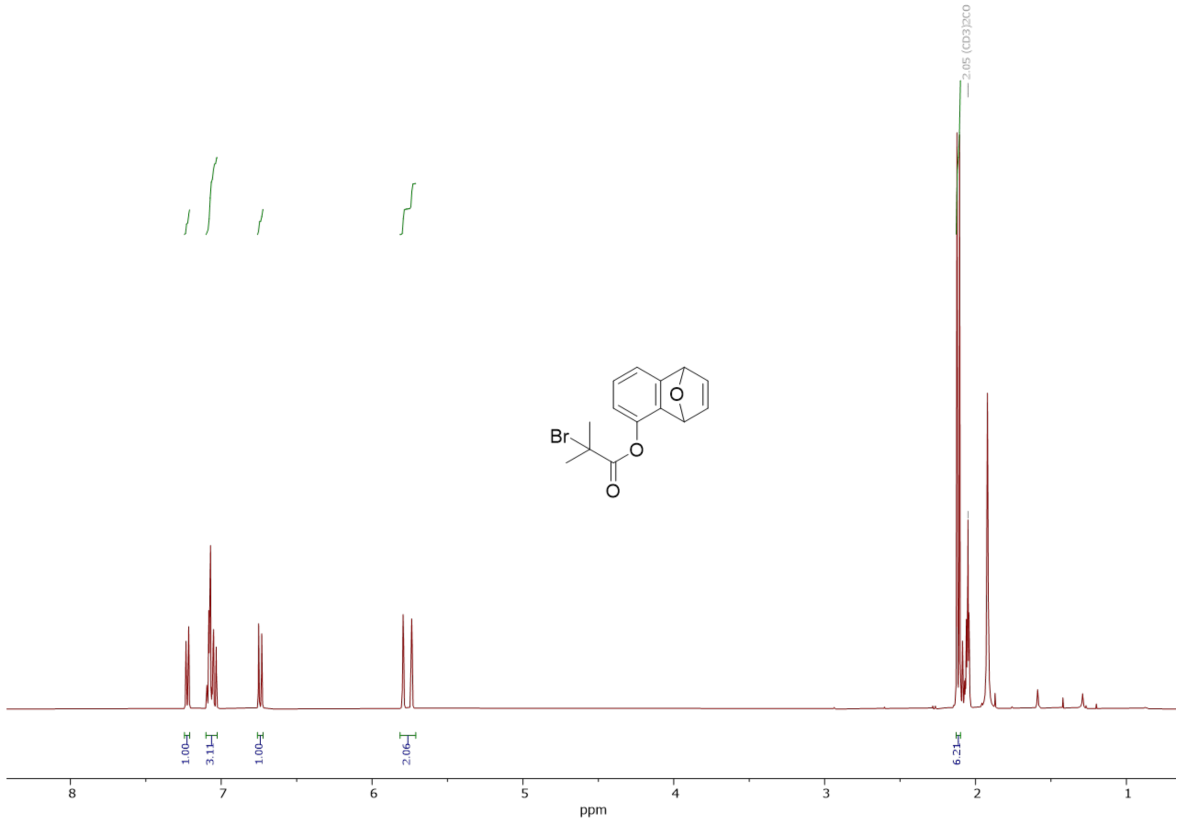

Spectrum S14. ^{1}H NMR (400 MHz, Acetone-d_6, 298 K) spectrum of compound **S5**.

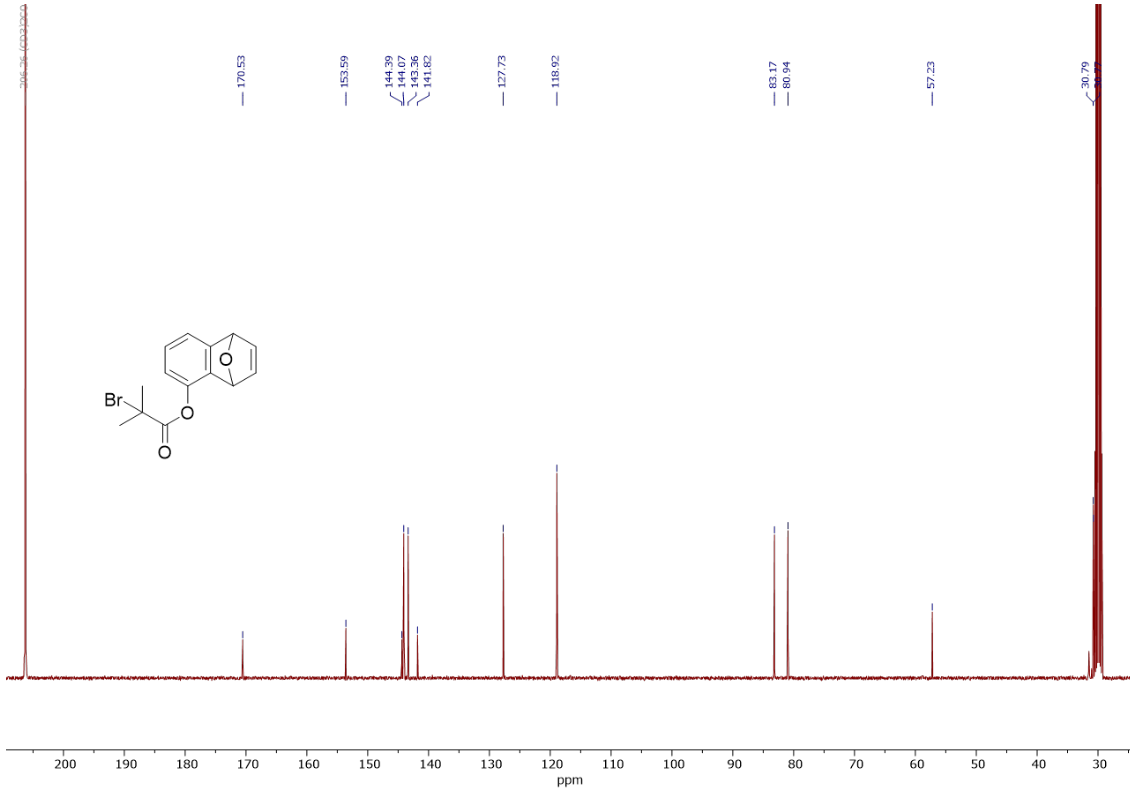

Spectrum S15. ^{13}C NMR (101 MHz, Acetone-d_6, 298 K) spectrum of compound **S5**.

6.1.9 Spectrum of S6

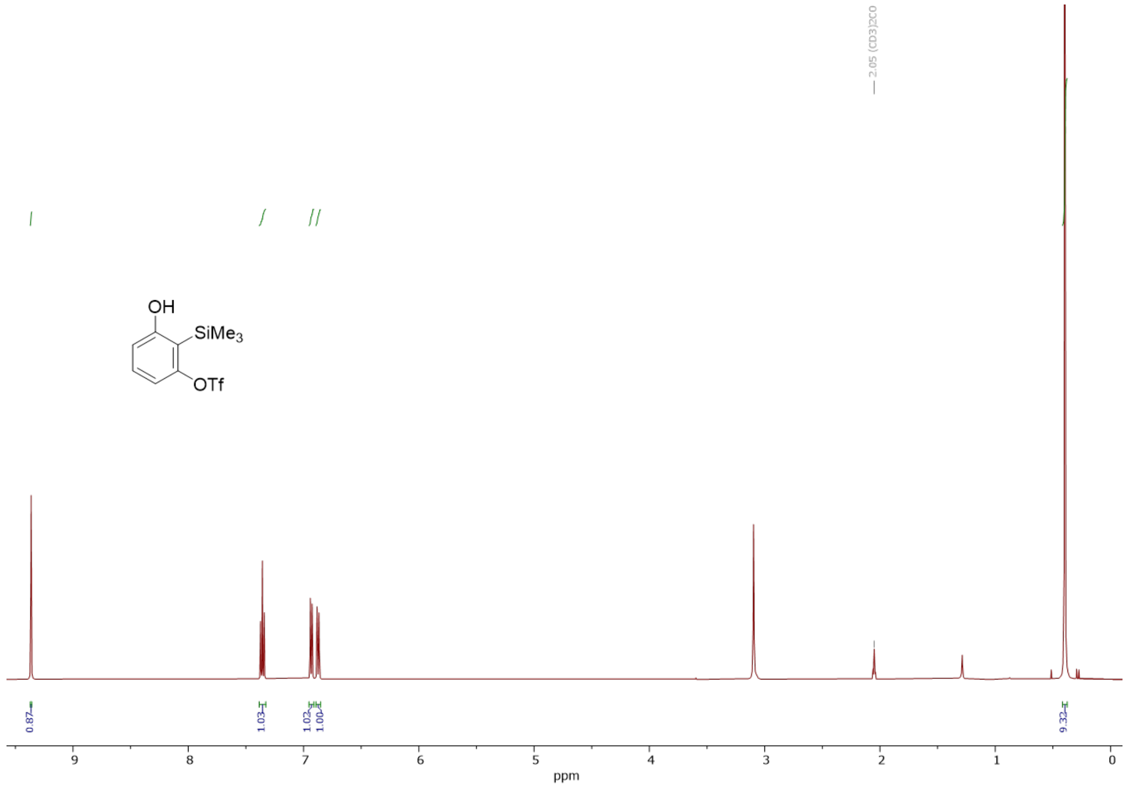

Spectrum S16. ^{1}H NMR (400 MHz, Acetone-d_6, 298 K) spectrum of compound **S6**

6.1.10 Spectrum of S7

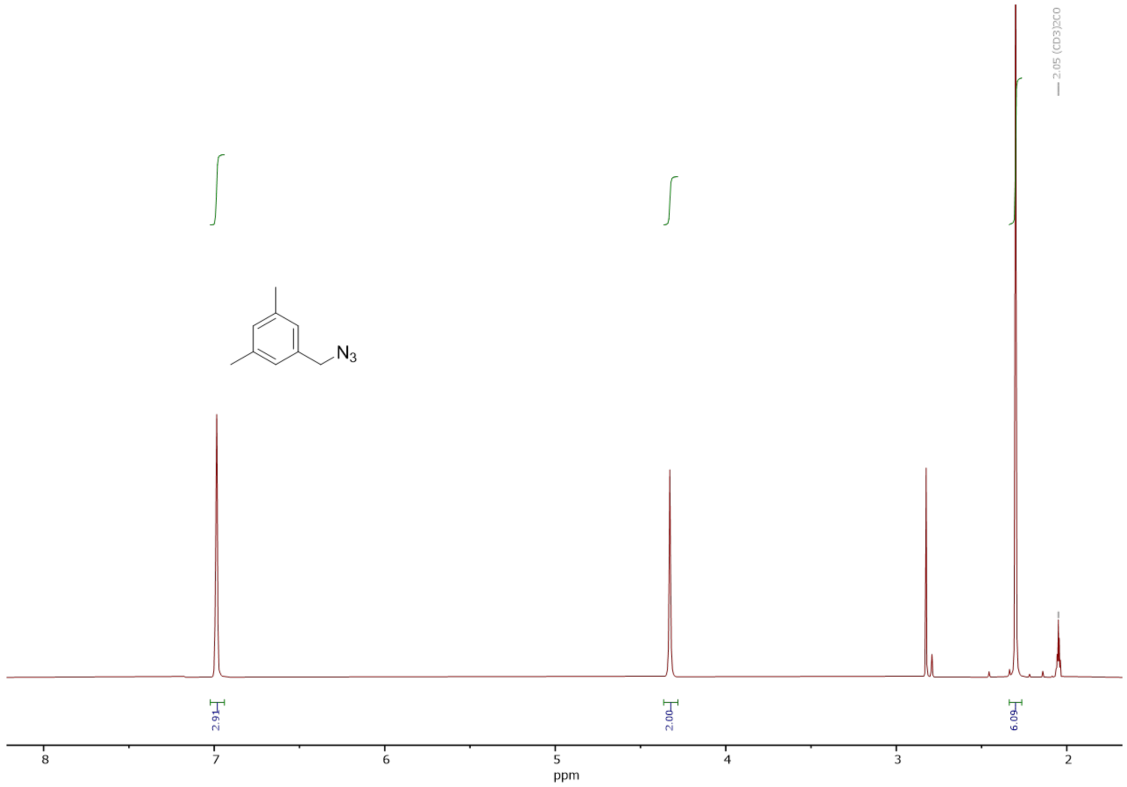

Spectrum S17. ^{1}H NMR (400 MHz, Acetone-d_6, 298 K) spectrum of compound **S7.**

6.1.11 Spectra of S8

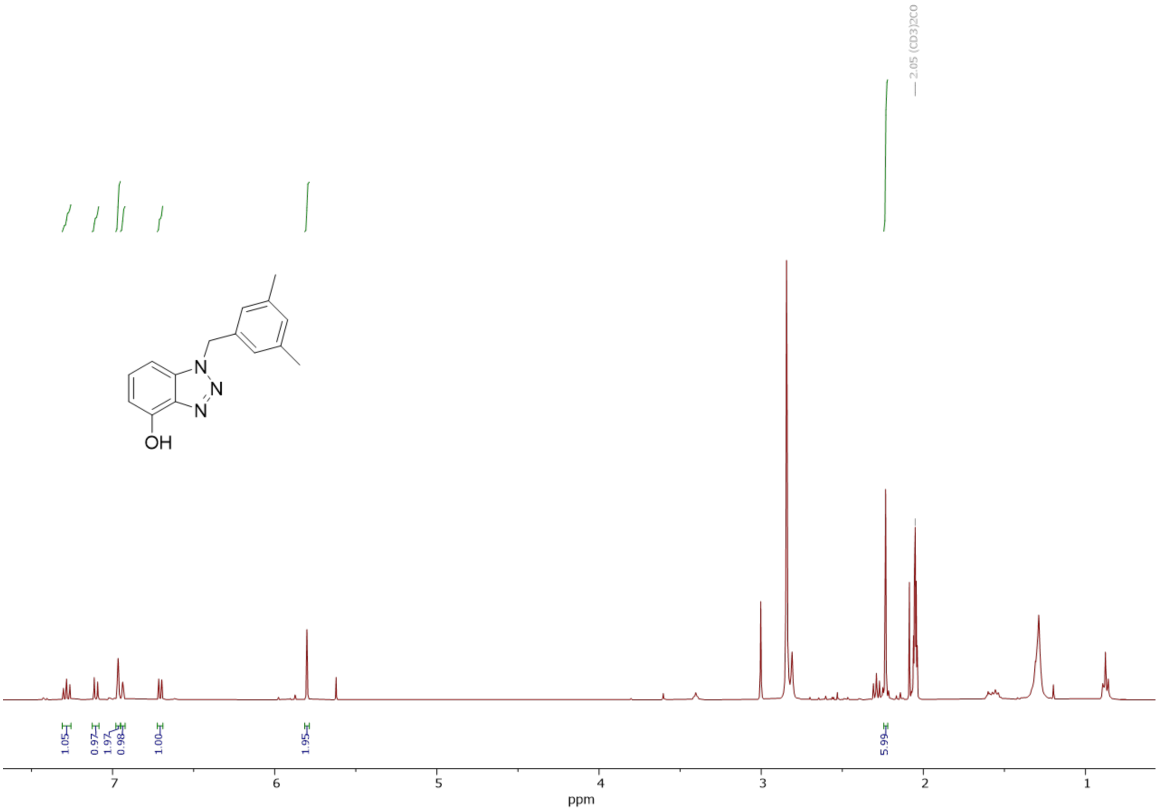

Spectrum S18. ^{1}H NMR (400 MHz, Acetone-d_6, 298 K) spectrum of compound **S8**.

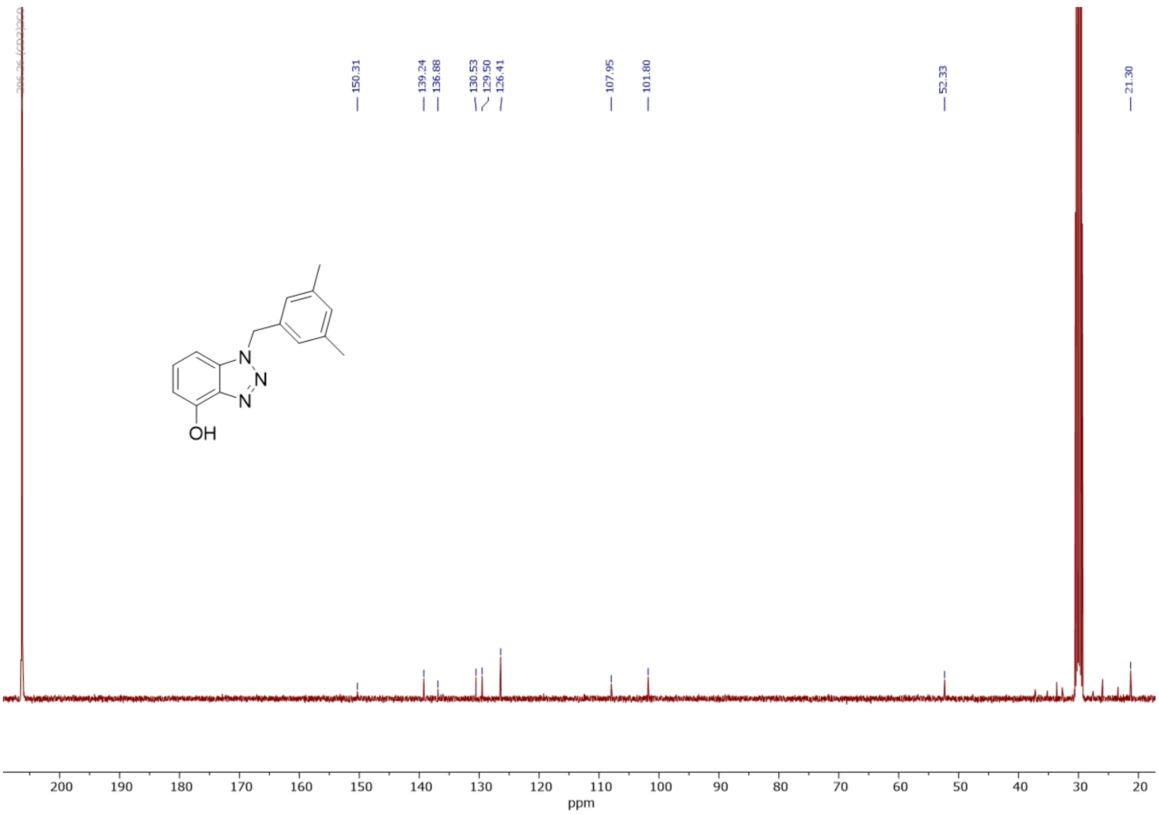

Spectrum S19. ^{13}C NMR (101 MHz, Acetone-d_6, 298 K) spectrum of compound **S8**.

6.1.12 Spectra of S9

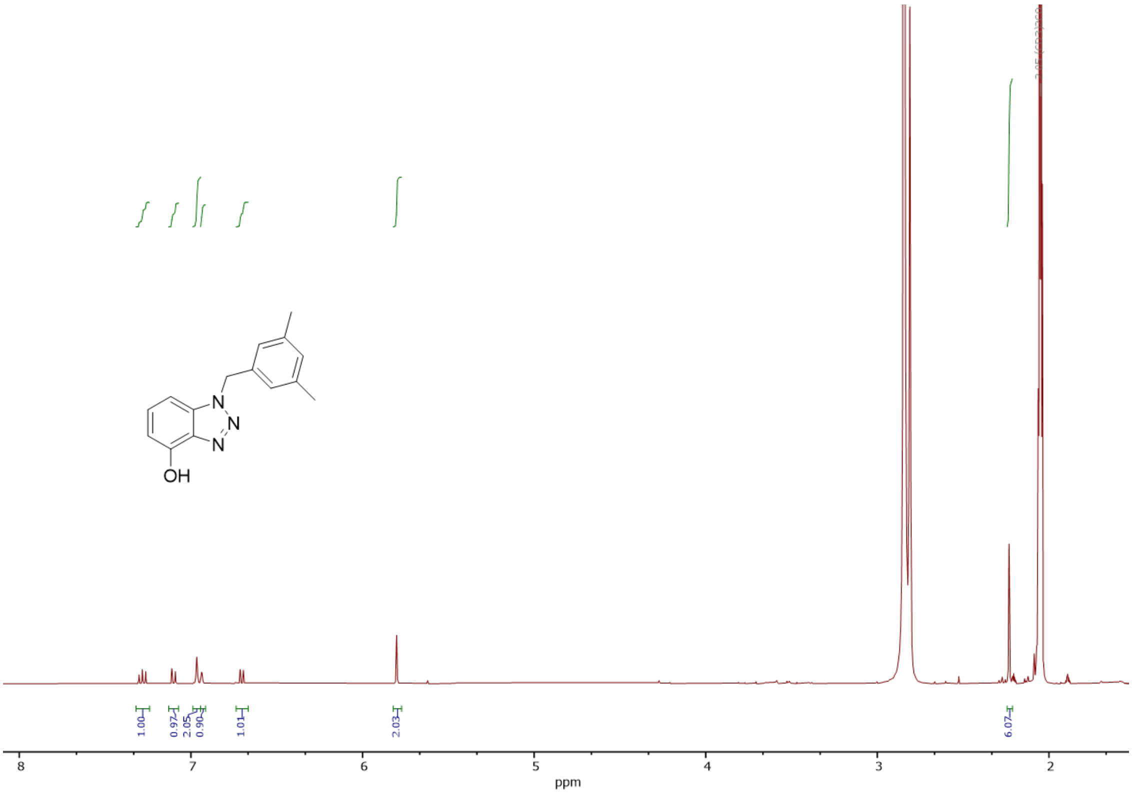

Spectrum S20. ^{1}H NMR (400 MHz, Acetone-d_6, 298 K) spectrum of compound **S9**.

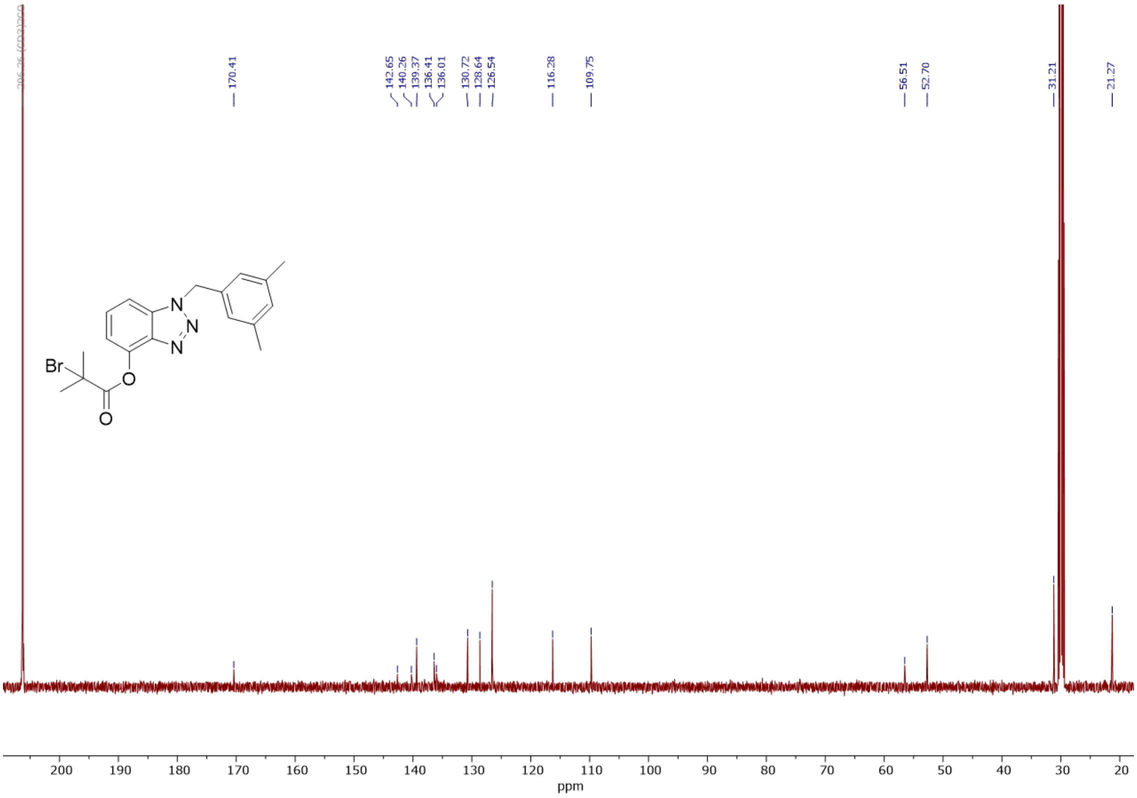

Spectrum S21. ^{13}C NMR (101 MHz, Acetone-d_6, 298 K) spectrum of compound **S9**.

6.1.13 Spectra of S10

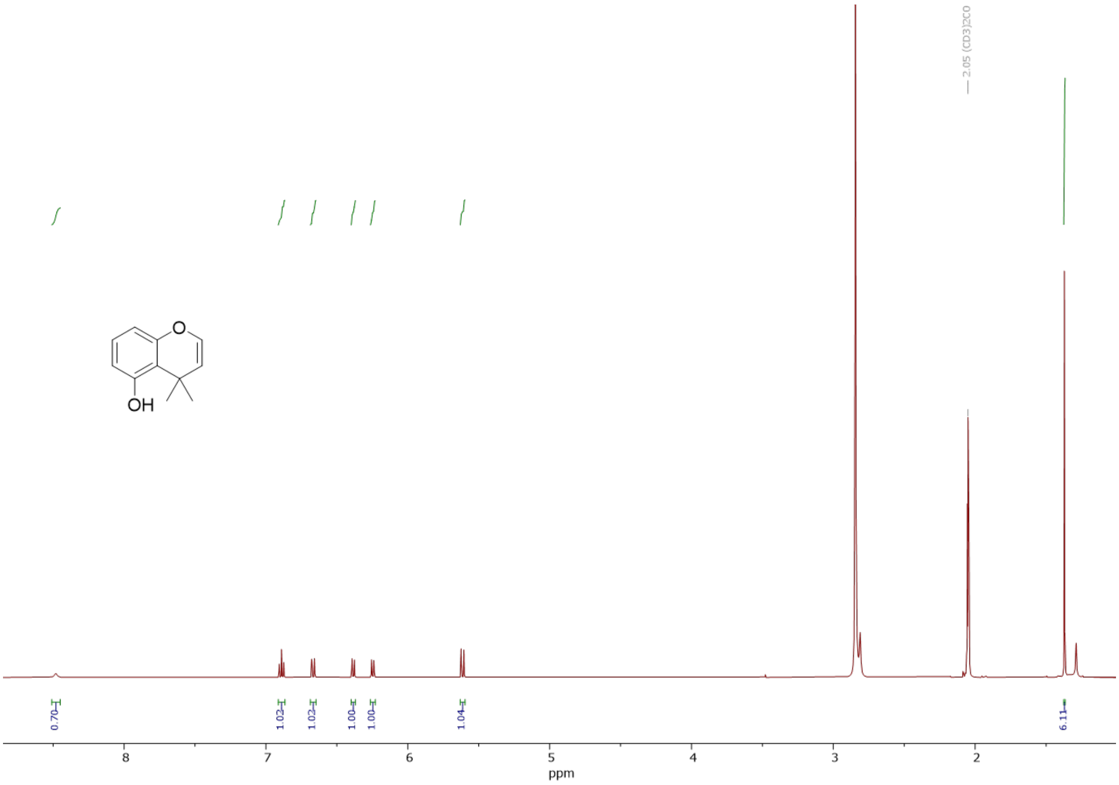

Spectrum S22. ^{1}H NMR (400 MHz, Acetone-d_6, 298 K) spectrum of compound **S10**.

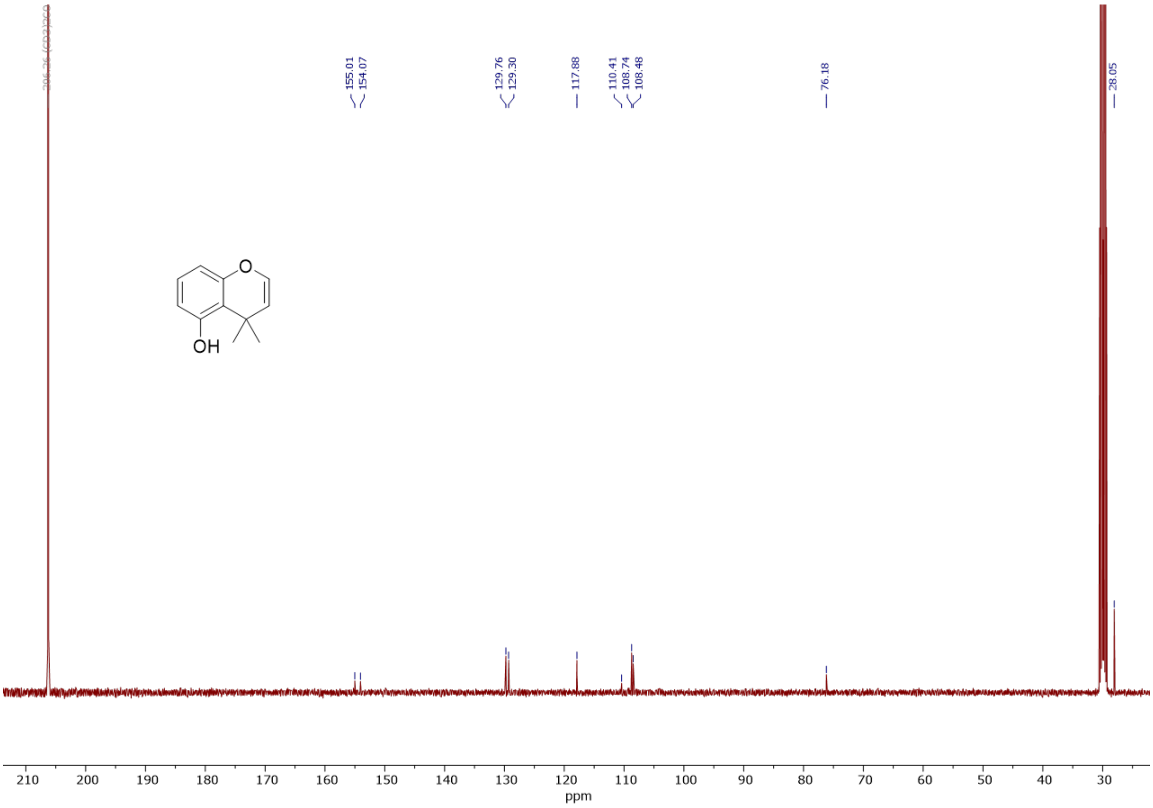

Spectrum S23. ^{13}C NMR (101 MHz, Acetone-d_6, 298 K) spectrum of compound **S10**.

6.1.14 Spectra of S11

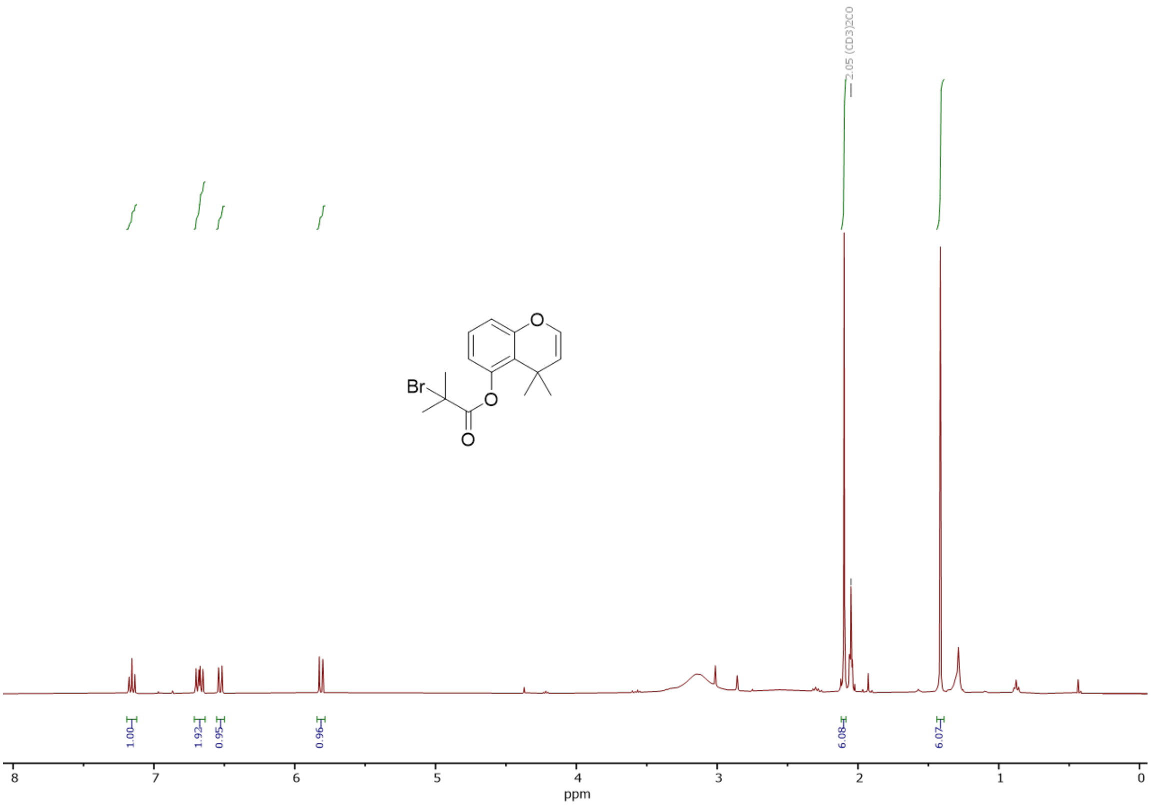

Spectrum S24. ^{1}H NMR (400 MHz, Acetone-d_6, 298 K) spectrum of compound **S11**.

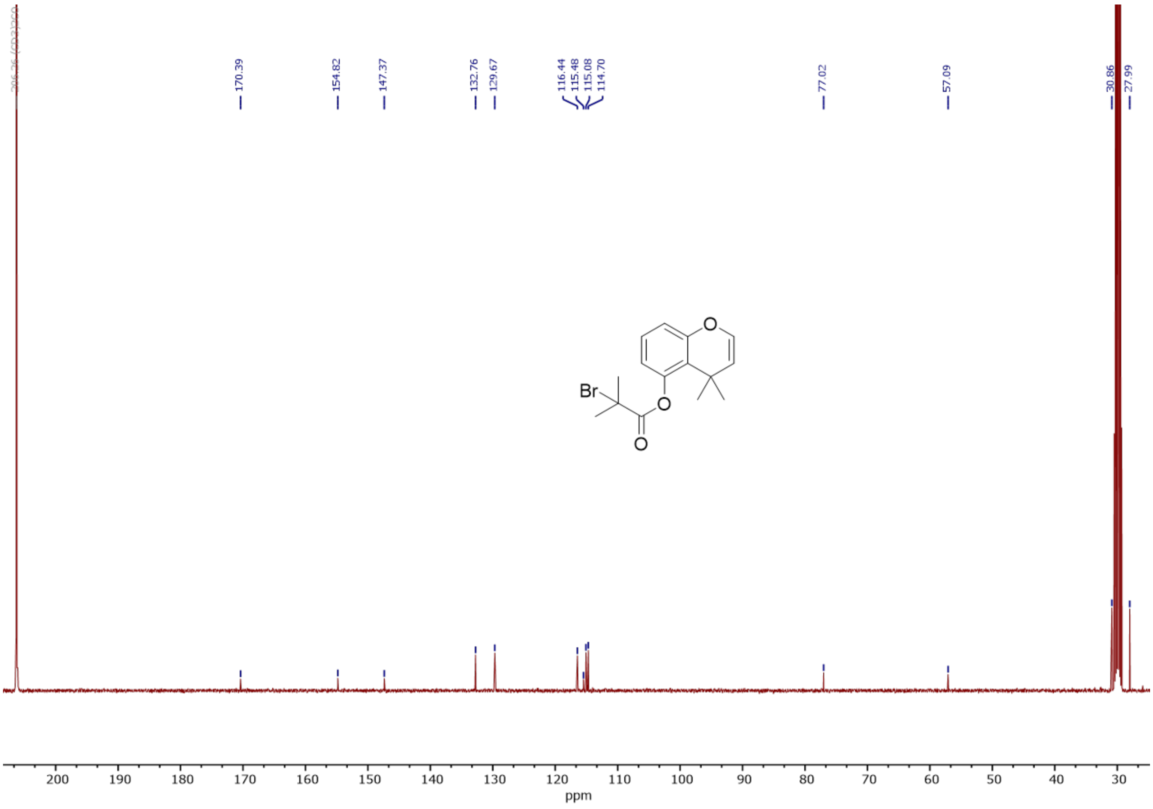

Spectrum S25. ^{13}C NMR (101 MHz, Acetone-d_6, 298 K) spectrum of compound **S11**.

6.1.15 Spectra of S12

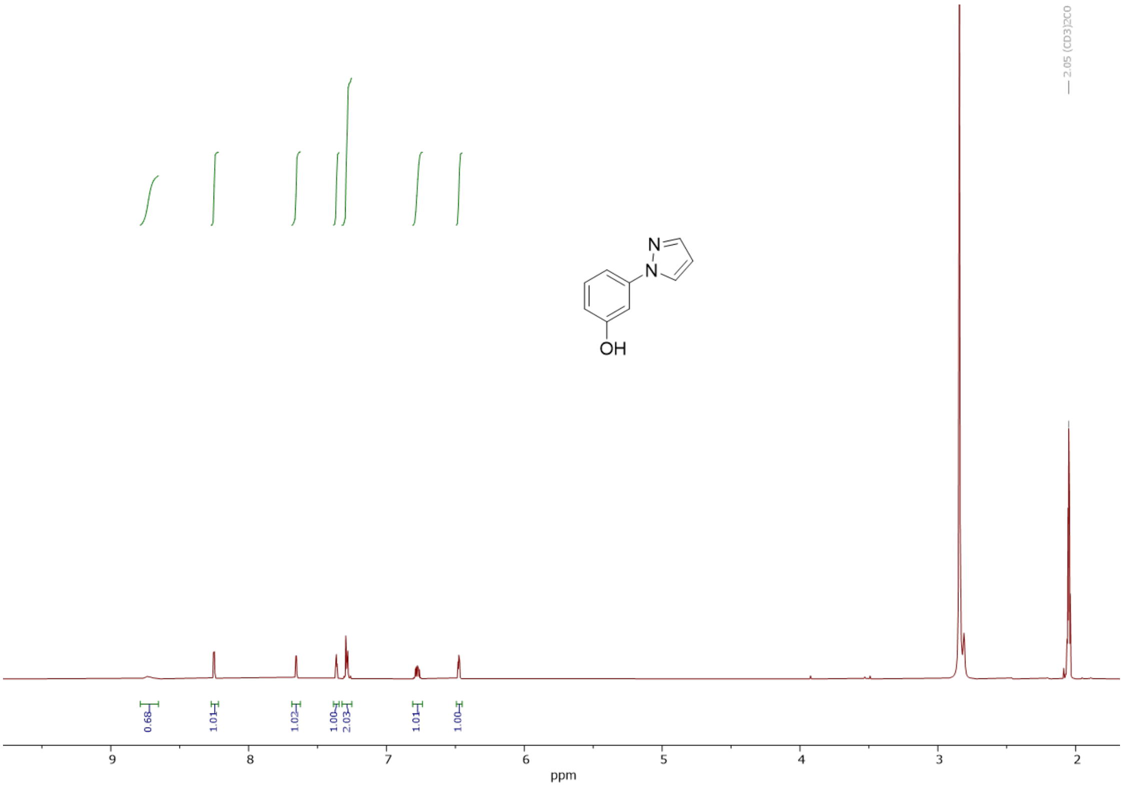

Spectrum S26. ^{1}H NMR (400 MHz, Acetone-d_6, 298 K) spectrum of compound **S12**.

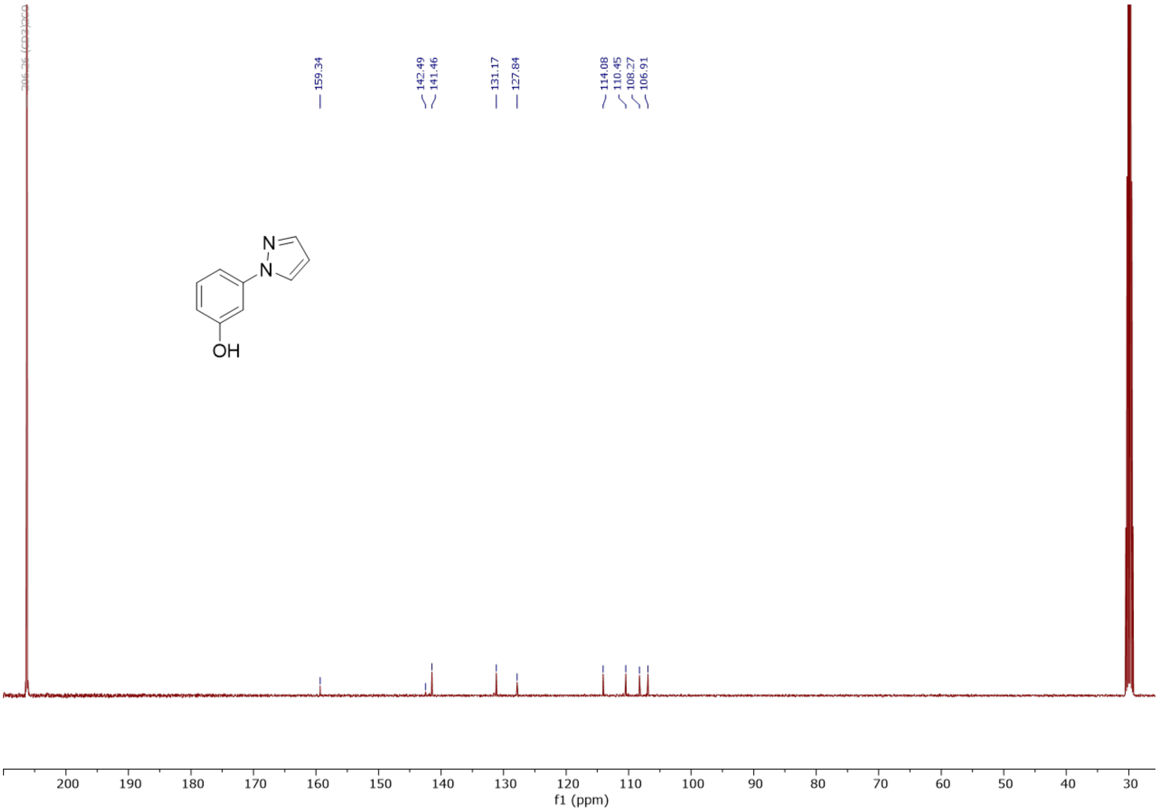

Spectrum S27. ^{13}C NMR (101 MHz, Acetone-d_6, 298 K) spectrum of compound **S12**.

6.1.16 Spectra of S13

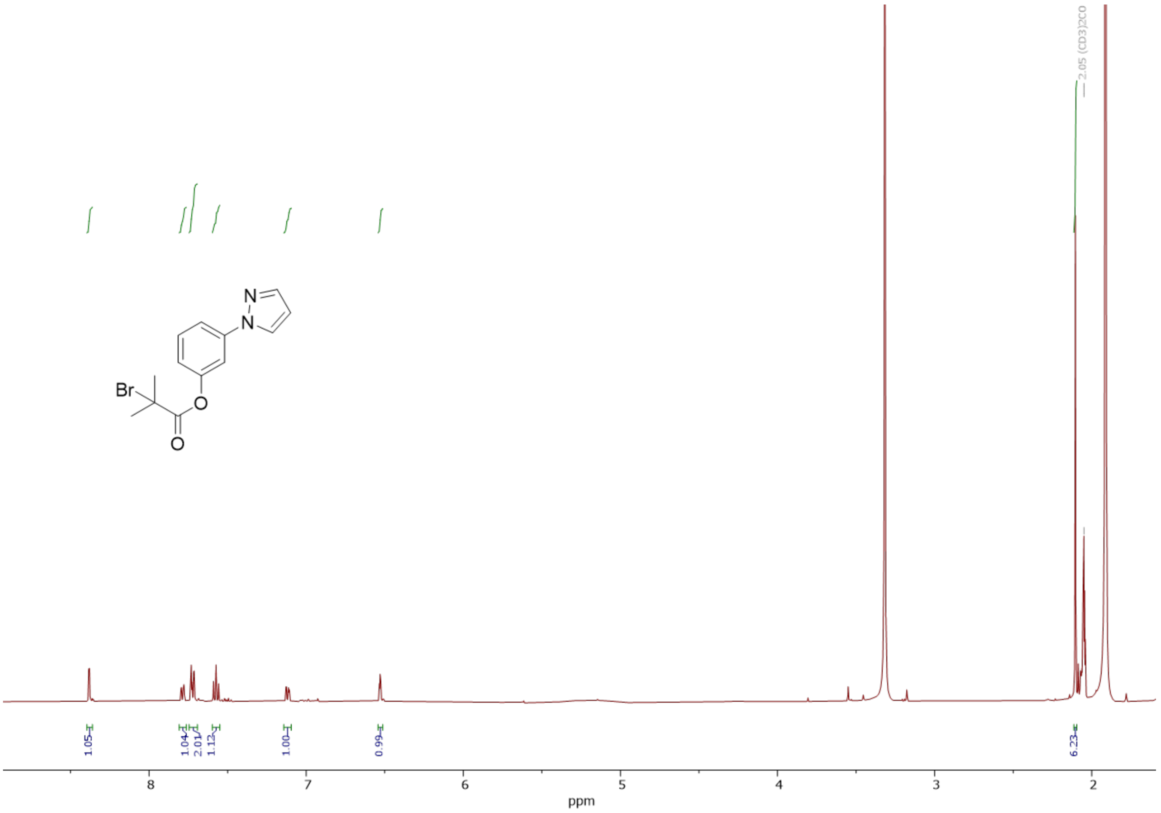

Spectrum S28. ^{1}H NMR (400 MHz, Acetone-d_6, 298 K) spectrum of compound **S13**.

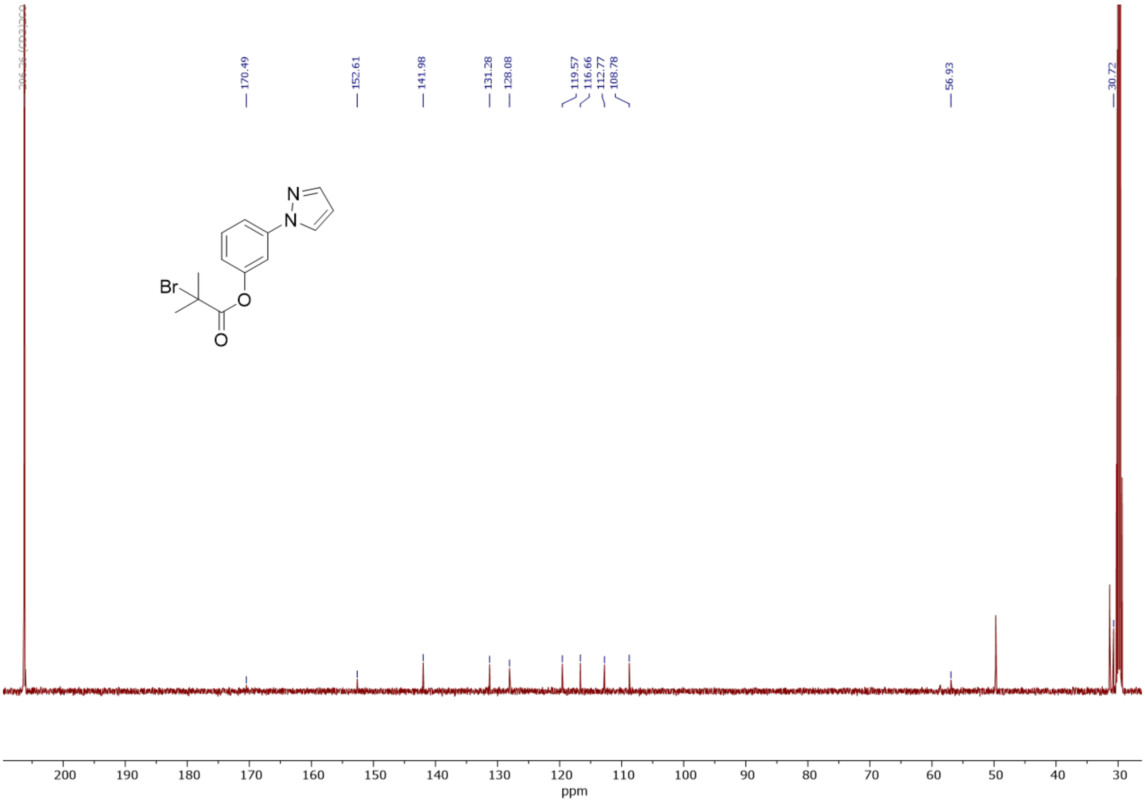

Spectrum S29. ^{13}C NMR (101 MHz, Acetone-d_6, 298 K) spectrum of compound **S13**.

6.1.17 Spectra of S14

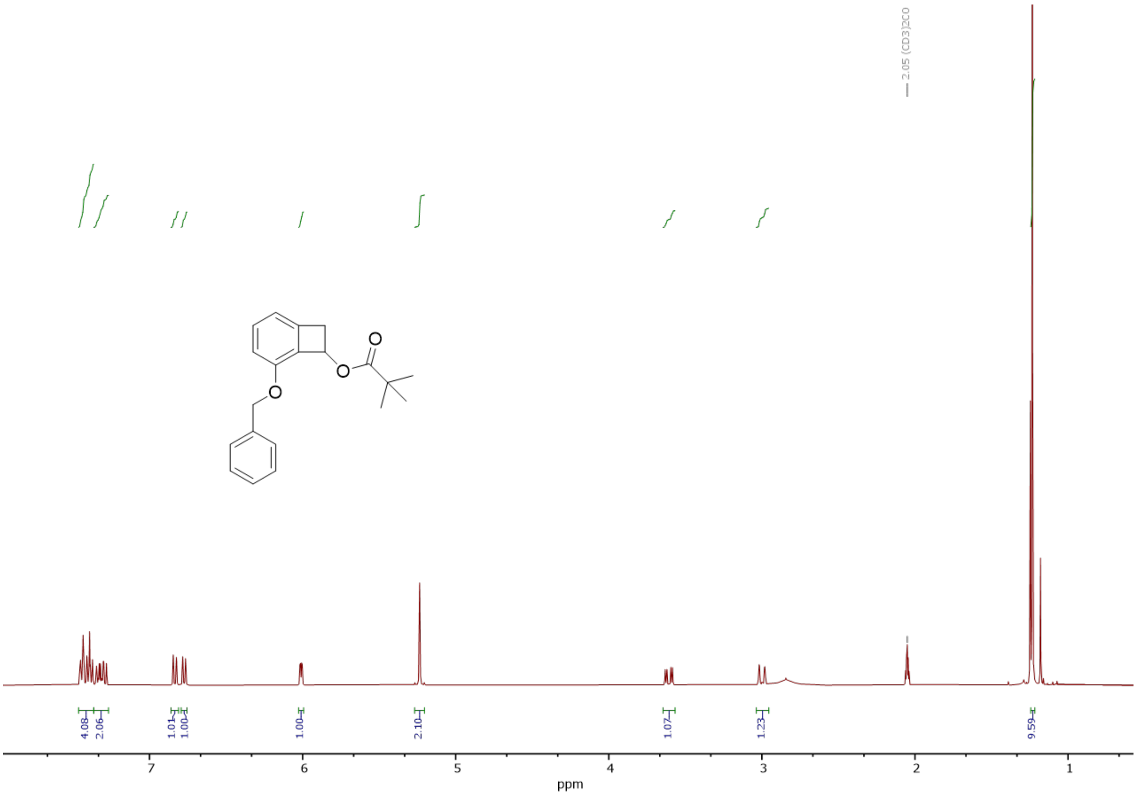

Spectrum S30. ^{1}H NMR (400 MHz, Acetone-d_6, 298 K) spectrum of compound **S14**.

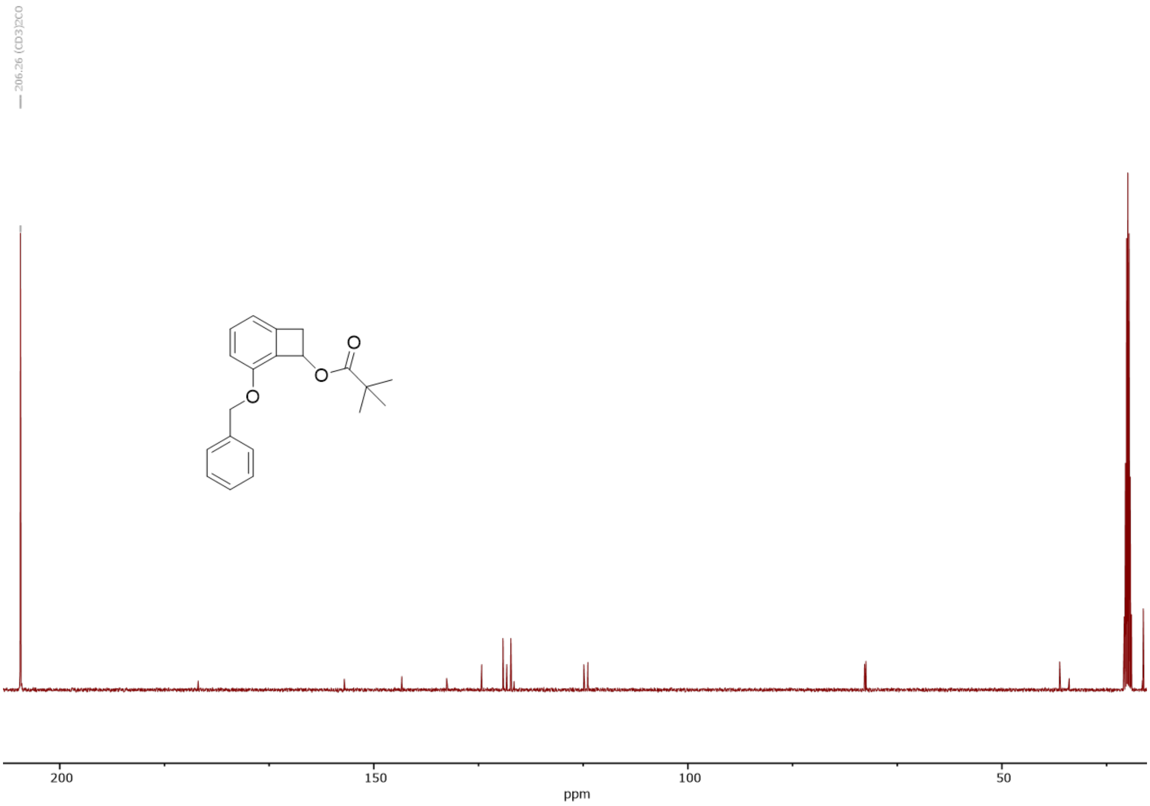

Spectrum S31. ^{13}C NMR (101 MHz, Acetone-d_6, 298 K) spectrum of compound **S14**.

6.1.18 Spectra of S15

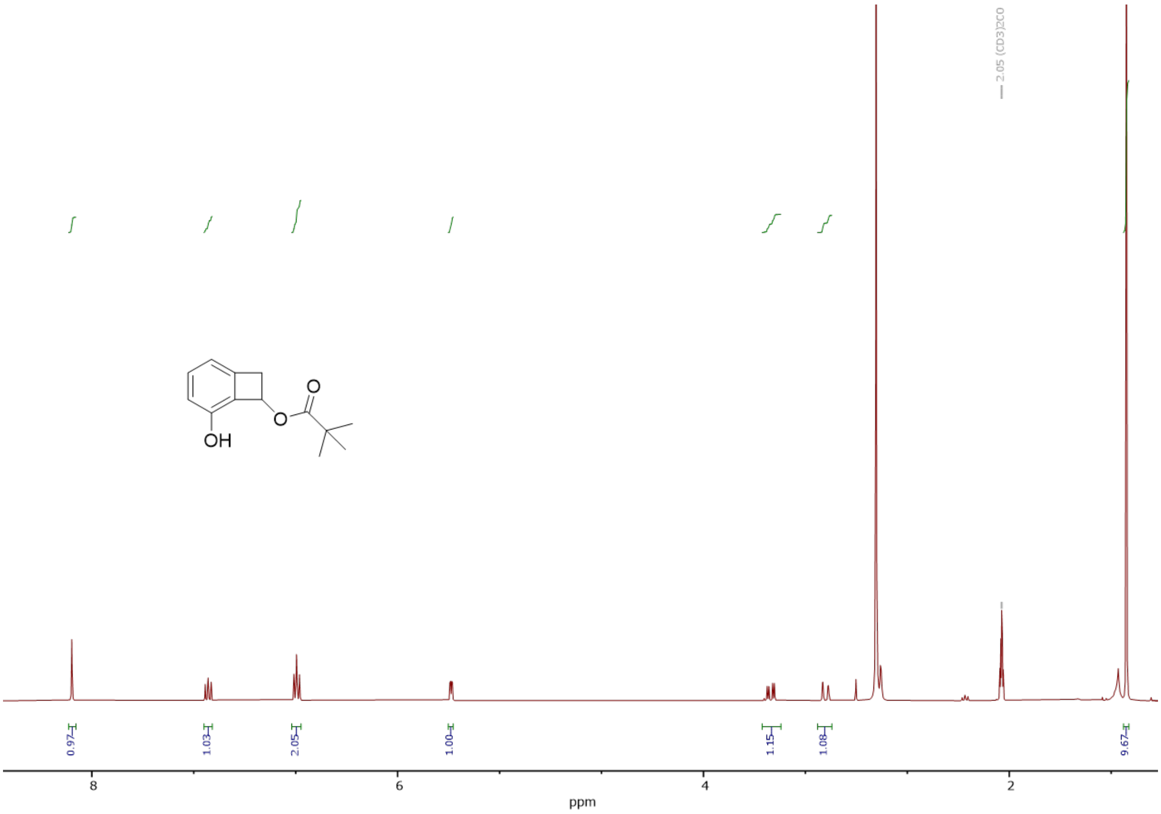

Spectrum S32. ^{1}H NMR (400 MHz, Acetone-d_6, 298 K) spectrum of compound **S15**.

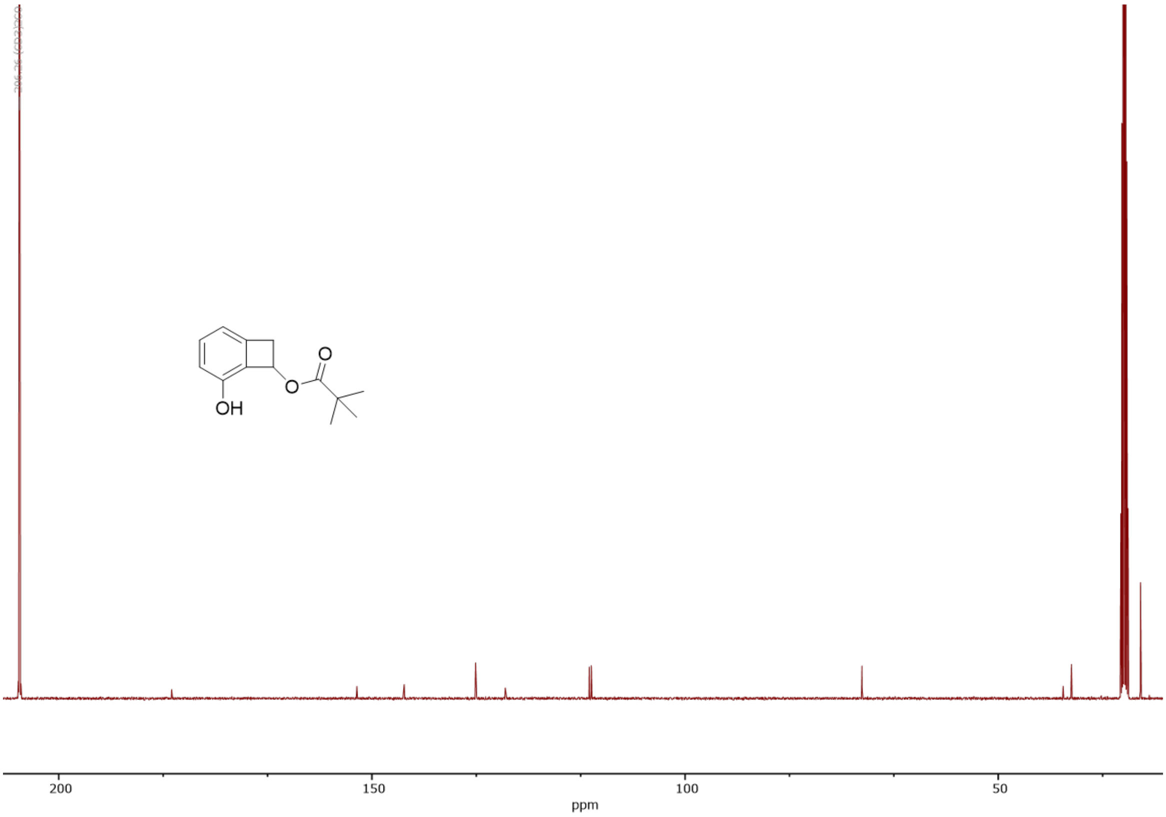

Spectrum S33. ^{13}C NMR (101 MHz, Acetone-d_6, 298 K) spectrum of compound **S15**.

6.1.19 Spectra of S16

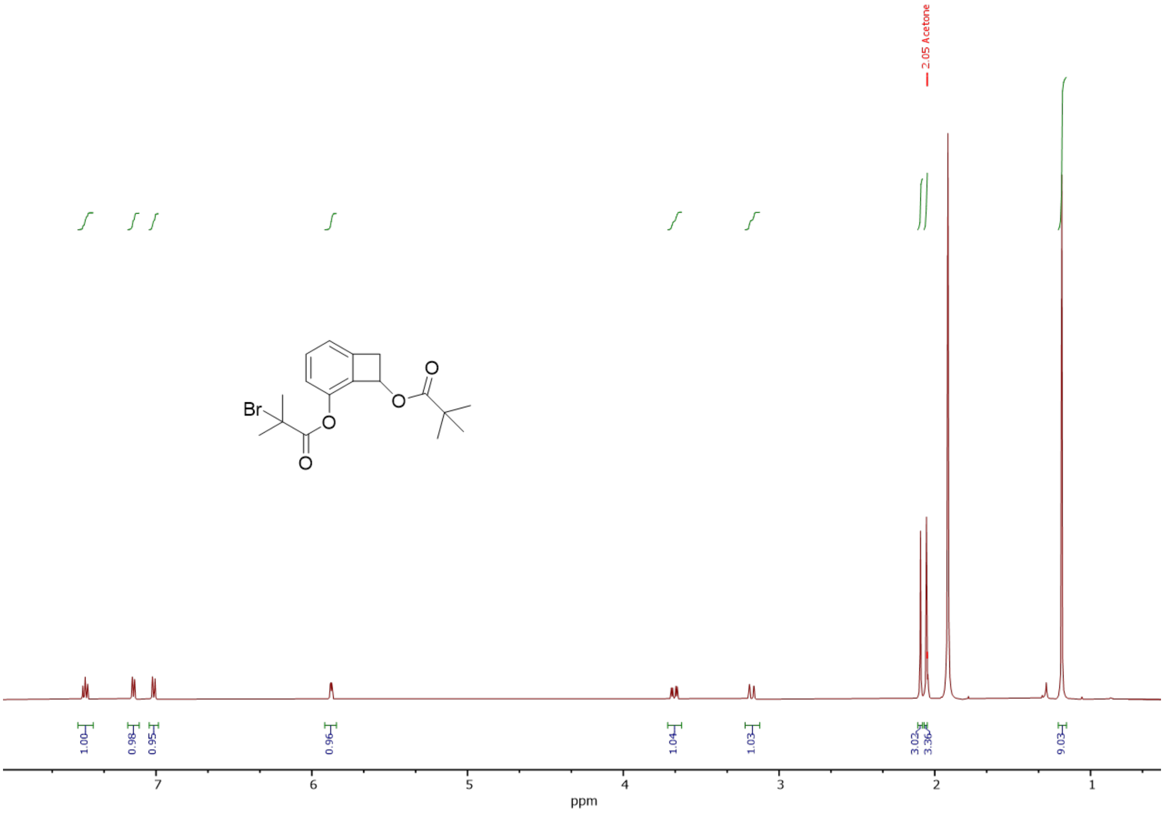

Spectrum S34. ^{1}H NMR (400 MHz, Acetone-d_6, 298 K) spectrum of compound **S16**.

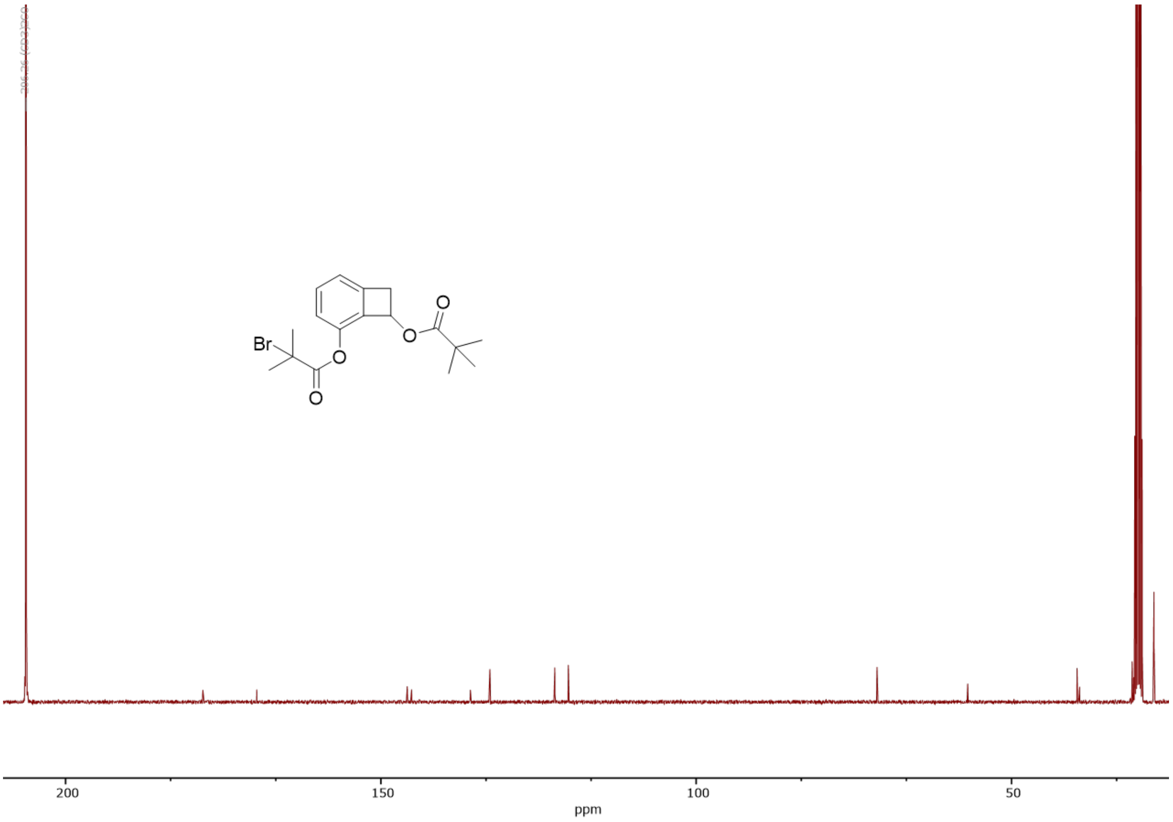

Spectrum S35. ^{13}C NMR (101 MHz, Acetone-d_6, 298 K) spectrum of compound **S16**.

6.2 Polymers NMR Spectra

6.2.1 Spectrum of 1

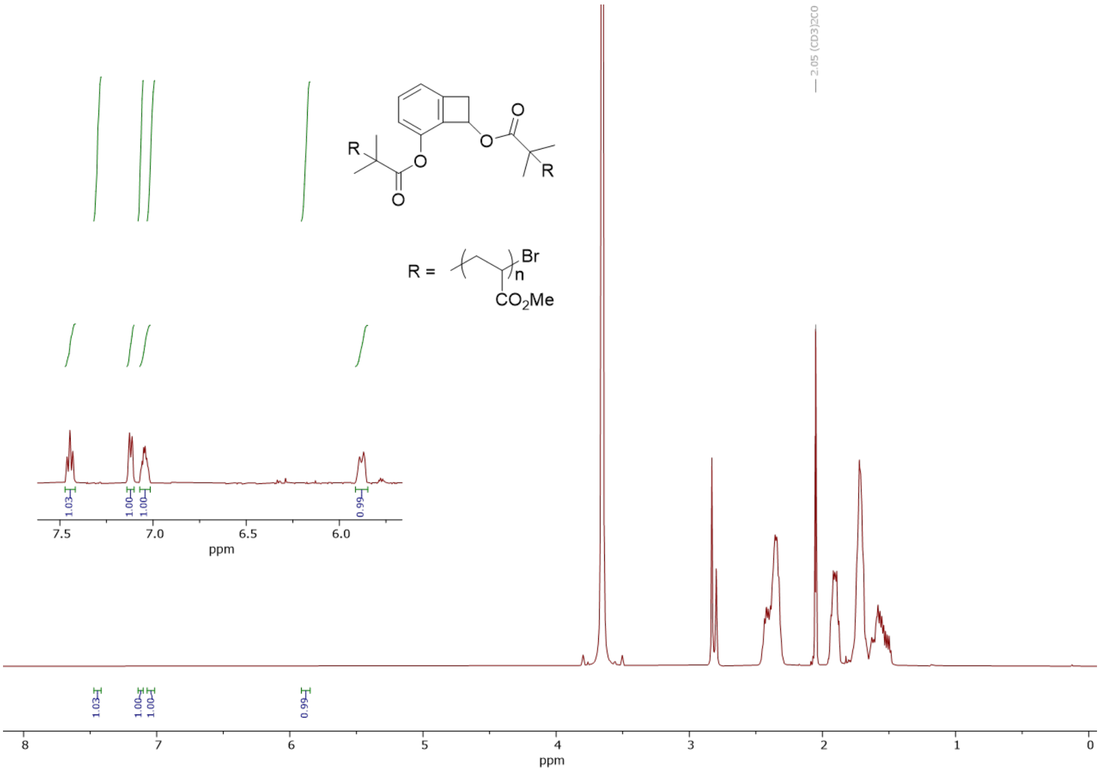

Spectrum S36. ^{1}H NMR (500 MHz, Acetone-d_6, 298 K) spectrum of polymer **1**.

6.2.2 Spectrum of 8_{ref}

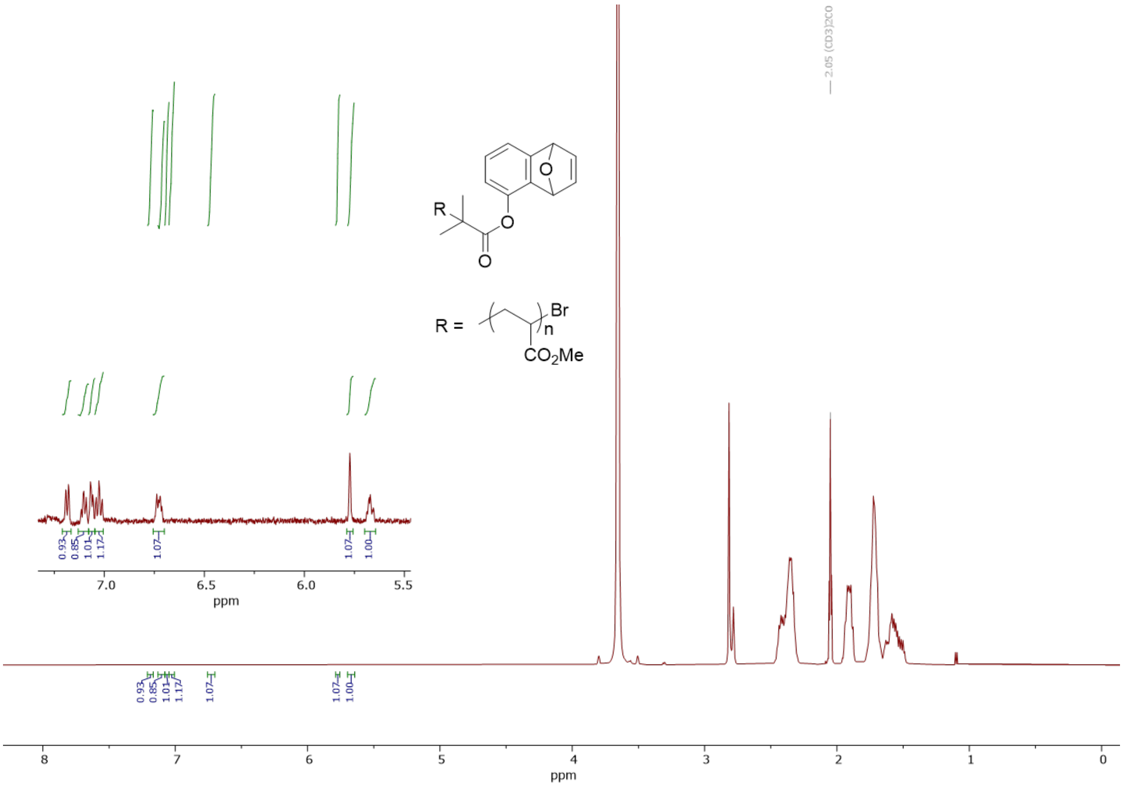

Spectrum S37. ^{1}H NMR (500 MHz, Acetone-d_6, 298 K) spectrum of polymer $\mathbf{8_{ref}}$.

6.2.3 Spectrum of S15

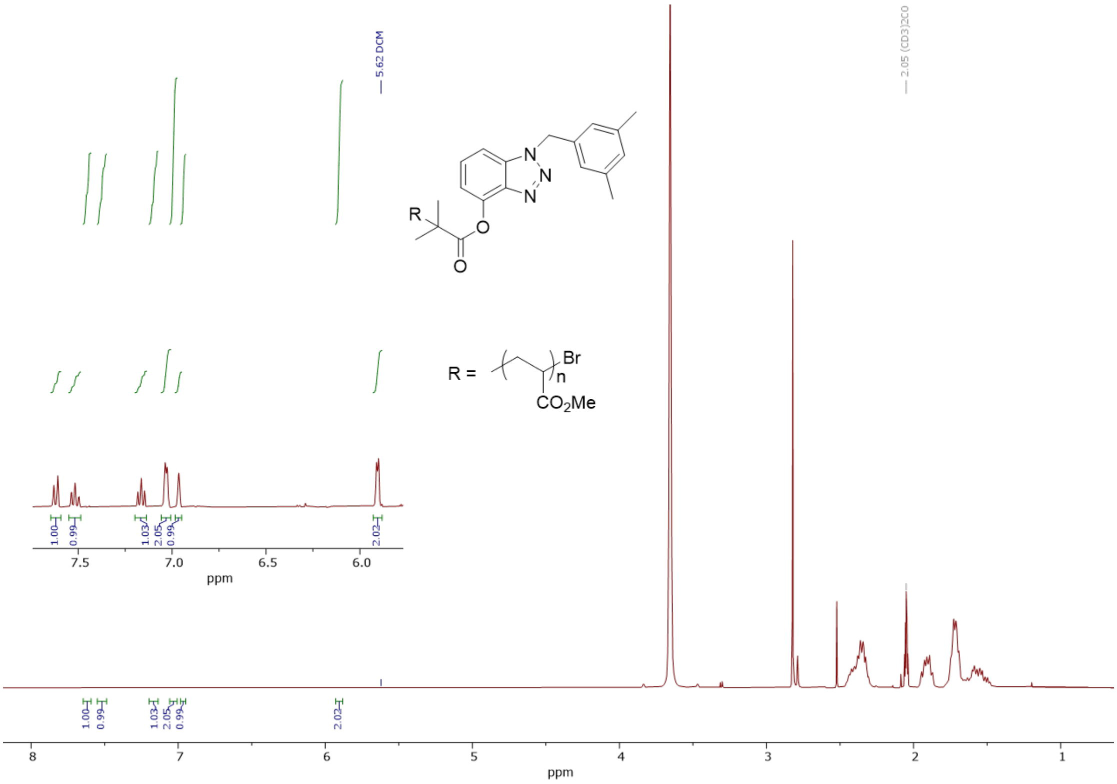

Spectrum S38. ^{1}H NMR (400 MHz, Acetone-d_6, 298 K) spectrum of polymer **S15**.

6.2.4 Spectrum of S16

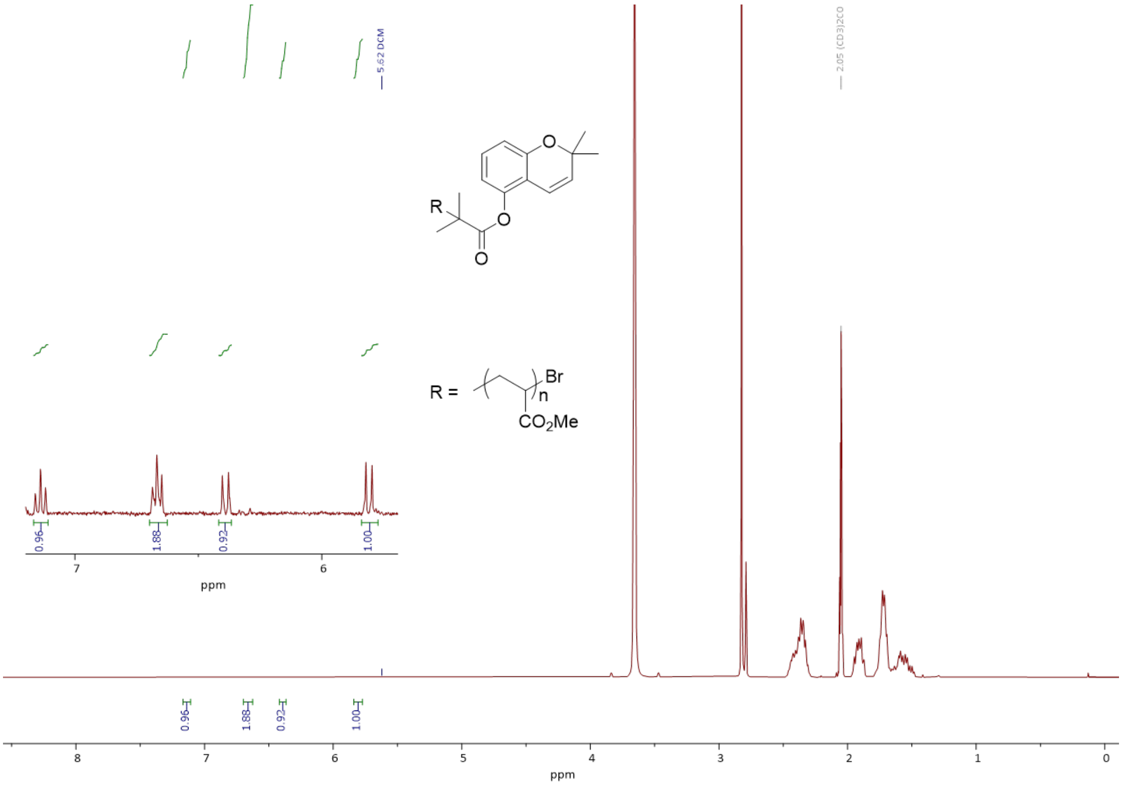

Spectrum S39. ^{1}H NMR (400 MHz, Acetone-d_6, 298 K) spectrum of polymer **S16**.

6.2.5 *Spectrum of S17*

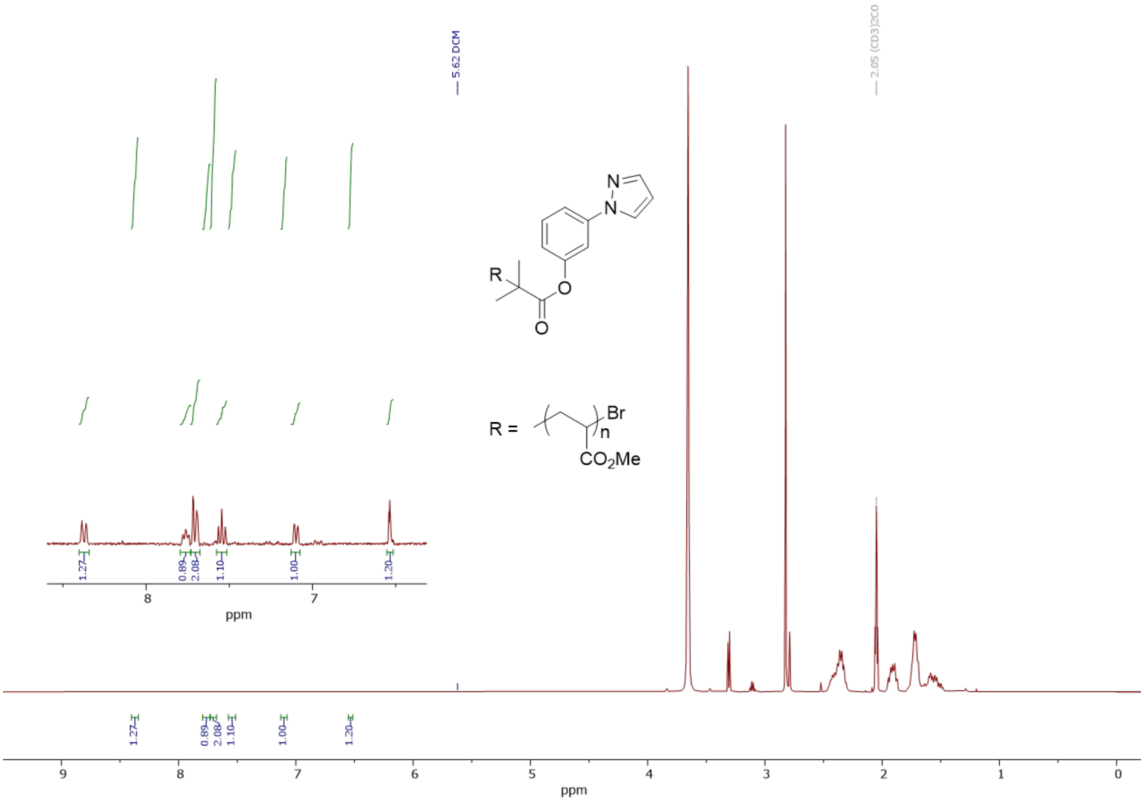

Spectrum S40. ^{1}H NMR (400 MHz, Acetone-d_6, 298 K) spectrum of polymer **S17**.

6.2.6 *Spectrum of S20*

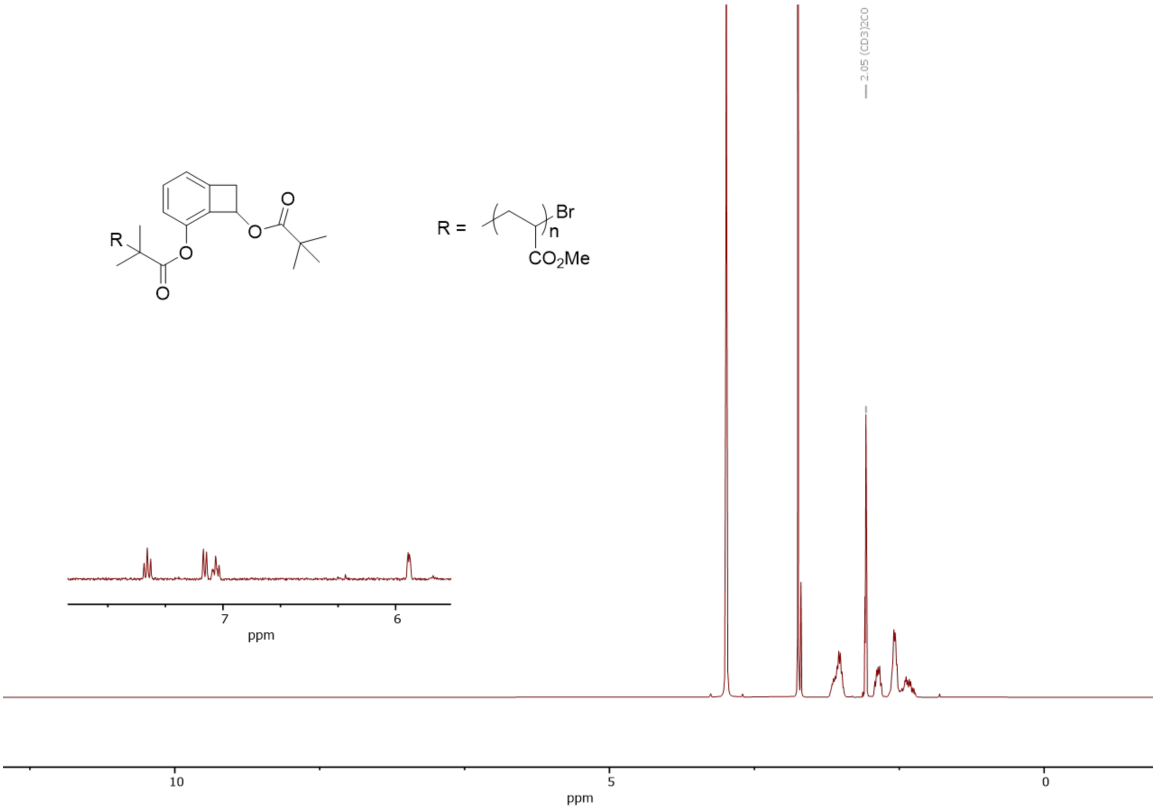

Spectrum S41. ^{1}H NMR (400 MHz, Acetone-d_6, 298 K) spectrum of polymer **S20**.

6.3 Post-Sonication NMR Spectra

6.3.1 Post-sonication ^{1}H NMR Spectra of Polymer 1 with Furan

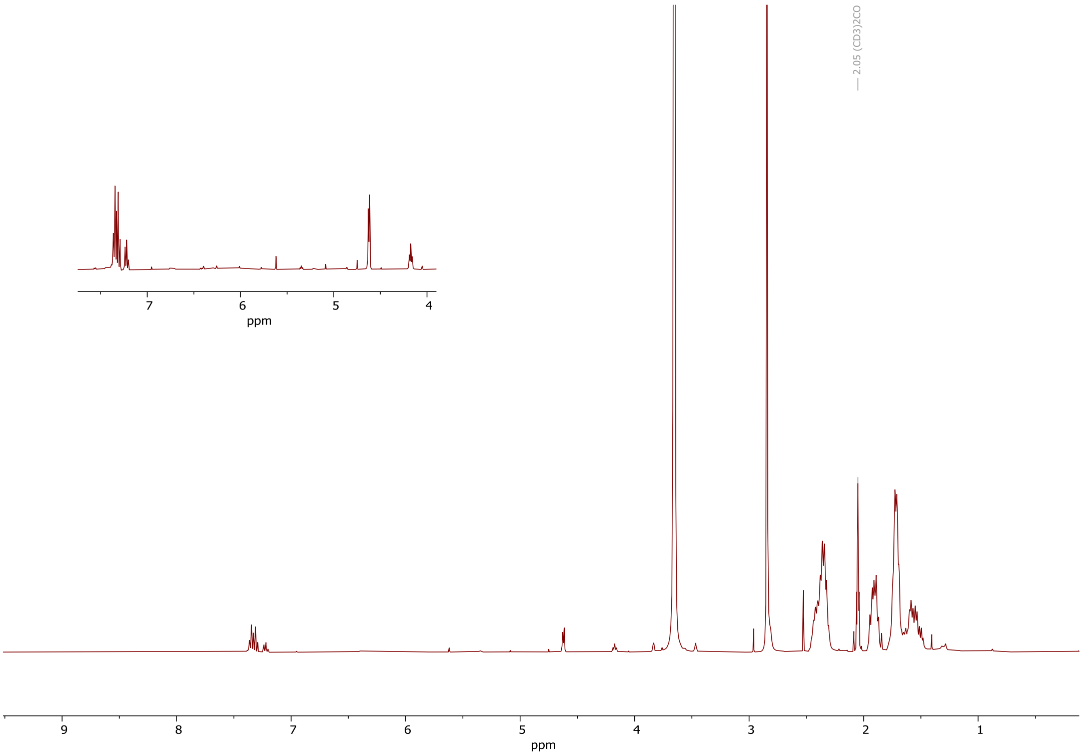

Spectrum S42. ^{1}H NMR (500 MHz, Acetone-d_6, 298 K) spectrum of post-sonication polymer **1** with furan before being washed with methanol.

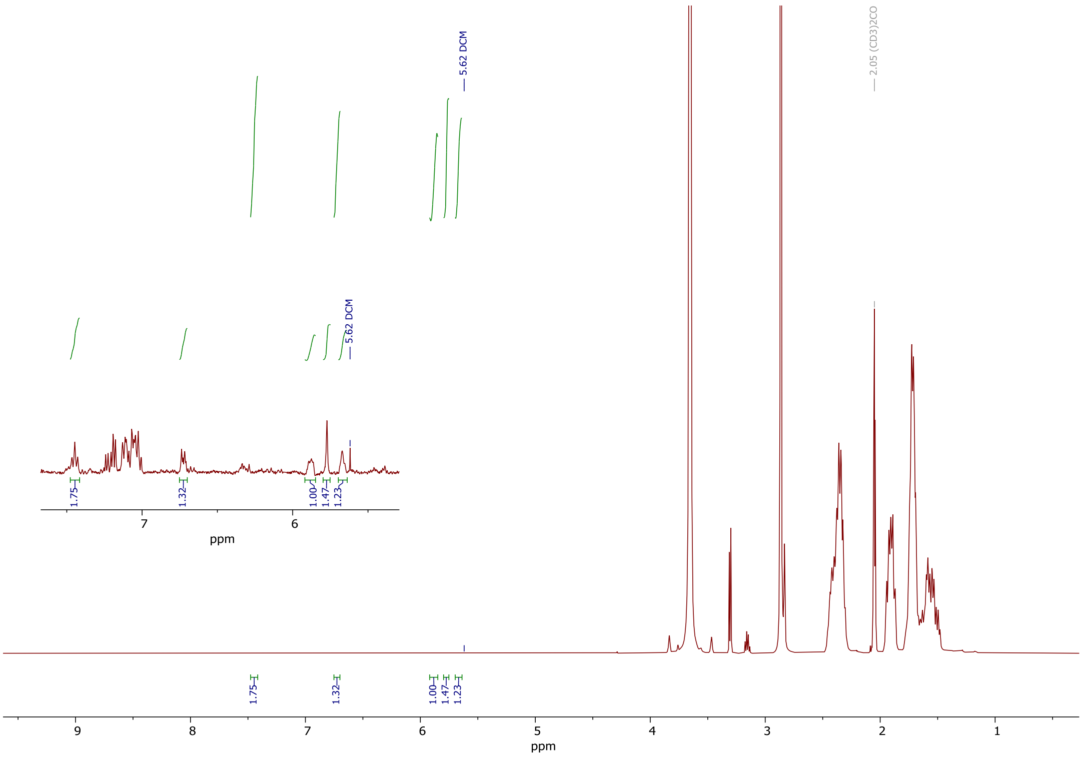

Spectrum S43. ^{1}H NMR (500 MHz, Acetone-d_6, 298 K) spectrum of post-sonication polymer **1** with furan after being washed with methanol.

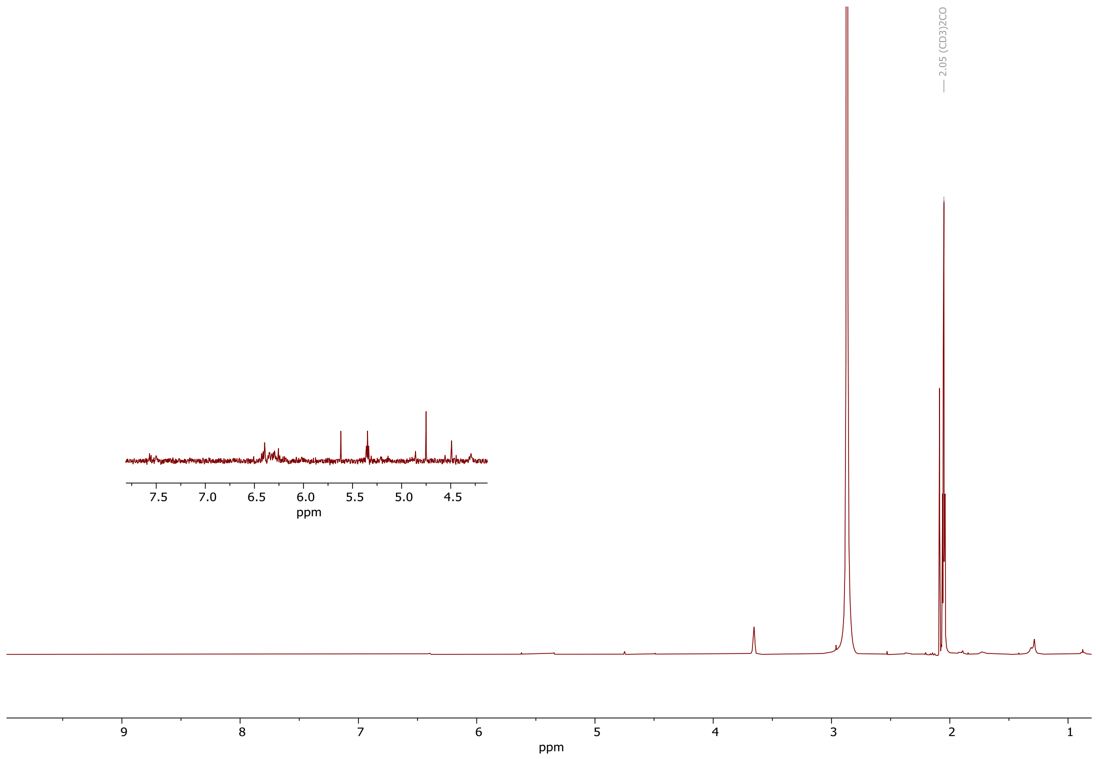

Spectrum S44. ^{1}H NMR (500 MHz, Acetone-d_6, 298 K) spectrum of concentrated methanol washings from post-sonication polymer **1** with furan.

6.3.2 Post-sonication ^{1}H NMR Spectra of Polymer 1 with S7

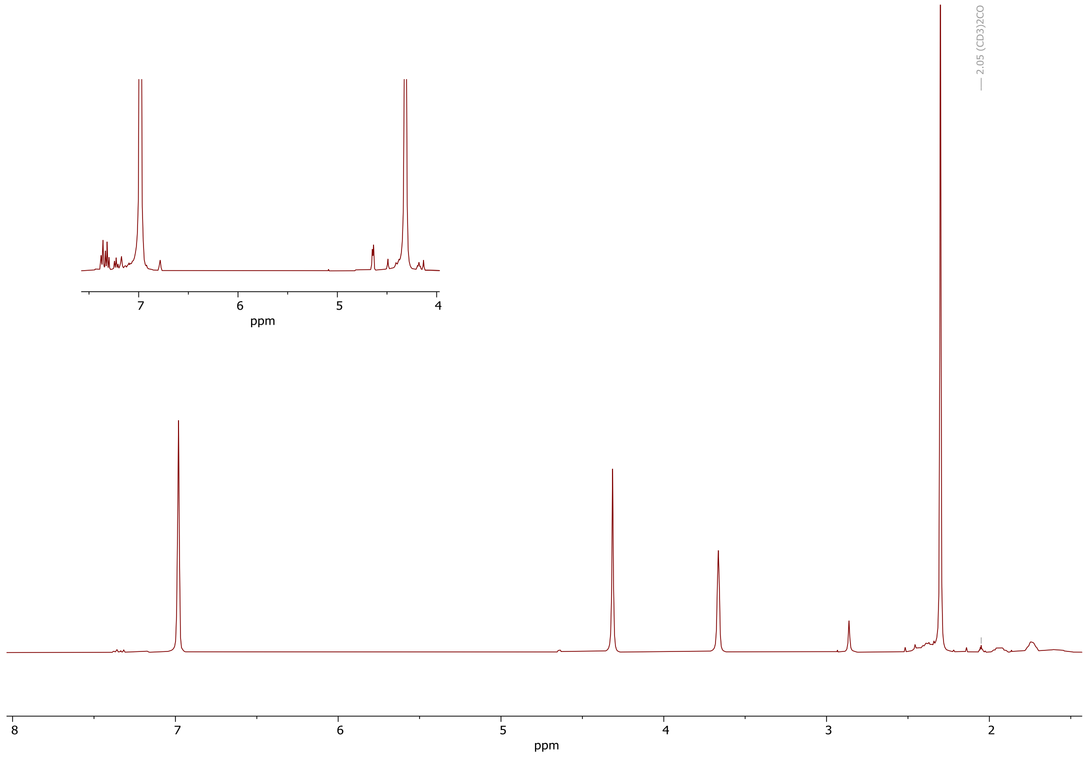

Spectrum S45. ^{1}H NMR (400 MHz, Acetone-d_6, 298 K) spectrum of post-sonication polymer **1** with **S7** before being washed with methanol.

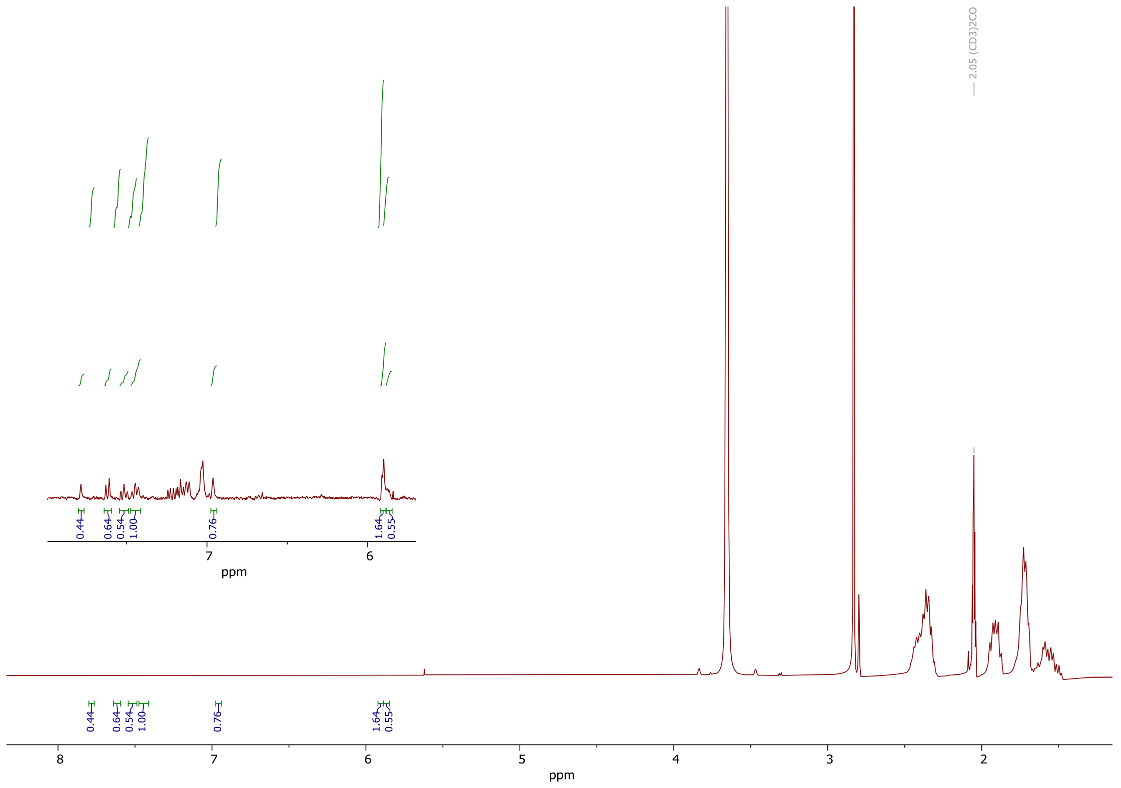

Spectrum S46. ^{1}H NMR (400 MHz, Acetone-d_6, 298 K) spectrum of post-sonication polymer **1** with **S7** after being washed with methanol.

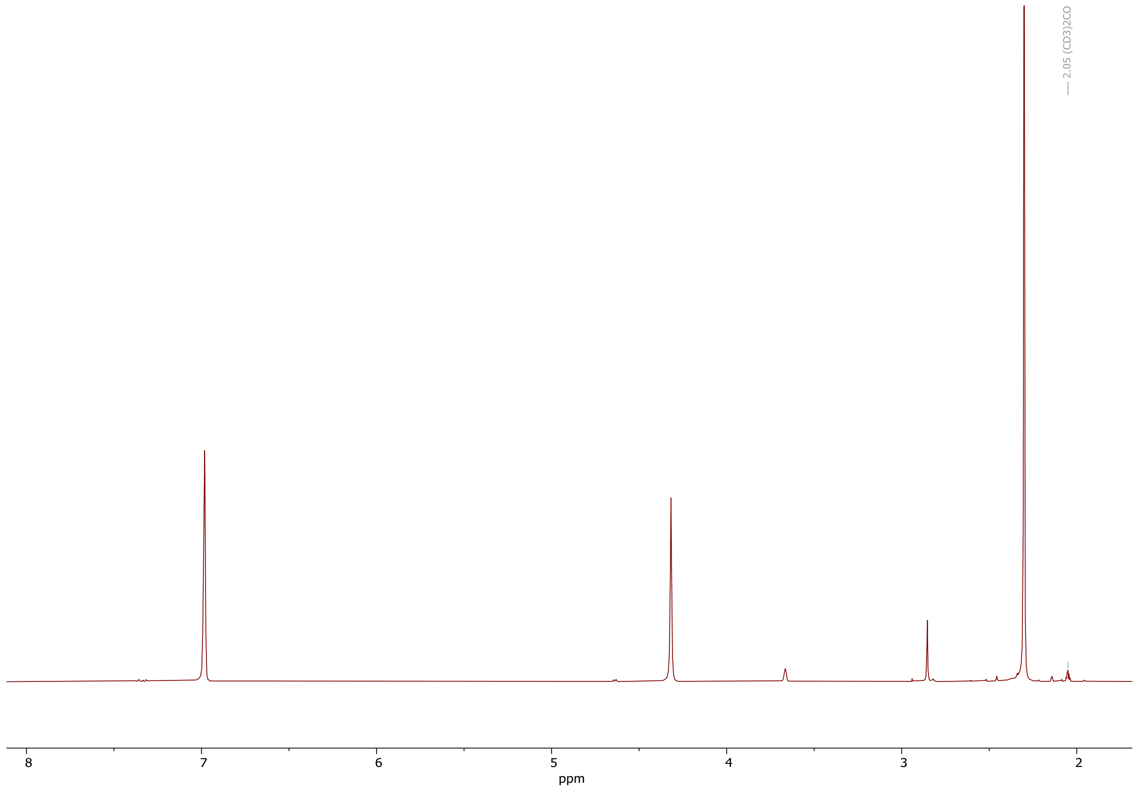

Spectrum S47. ^{1}H NMR (400 MHz, Acetone-d_6, 298 K) spectrum of concentrated methanol washings from post-sonication polymer **1** with **S7**.

6.3.3 Post-sonication ^{1}H NMR Spectra of Polymer 1 with 3-Methyl-2-butenal

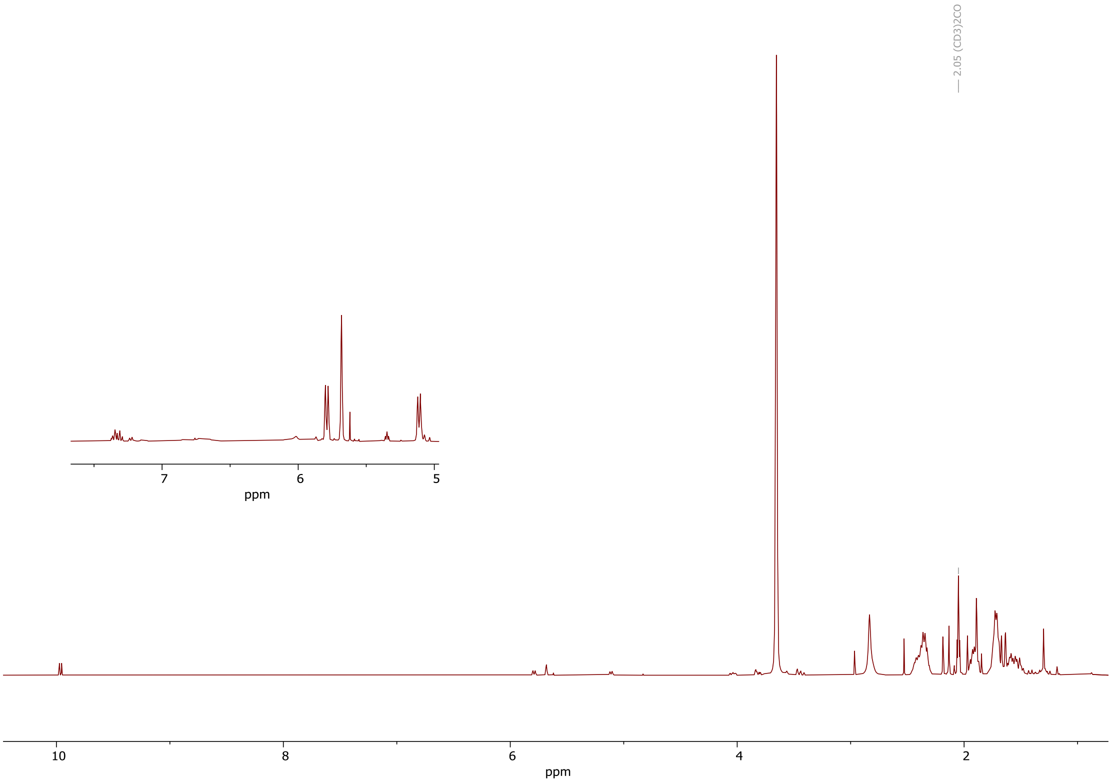

Spectrum S48. ^{1}H NMR (400 MHz, Acetone-d_6, 298 K) spectrum of post-sonication polymer **1** with **3-Methyl-2-butenal** before being washed with methanol.

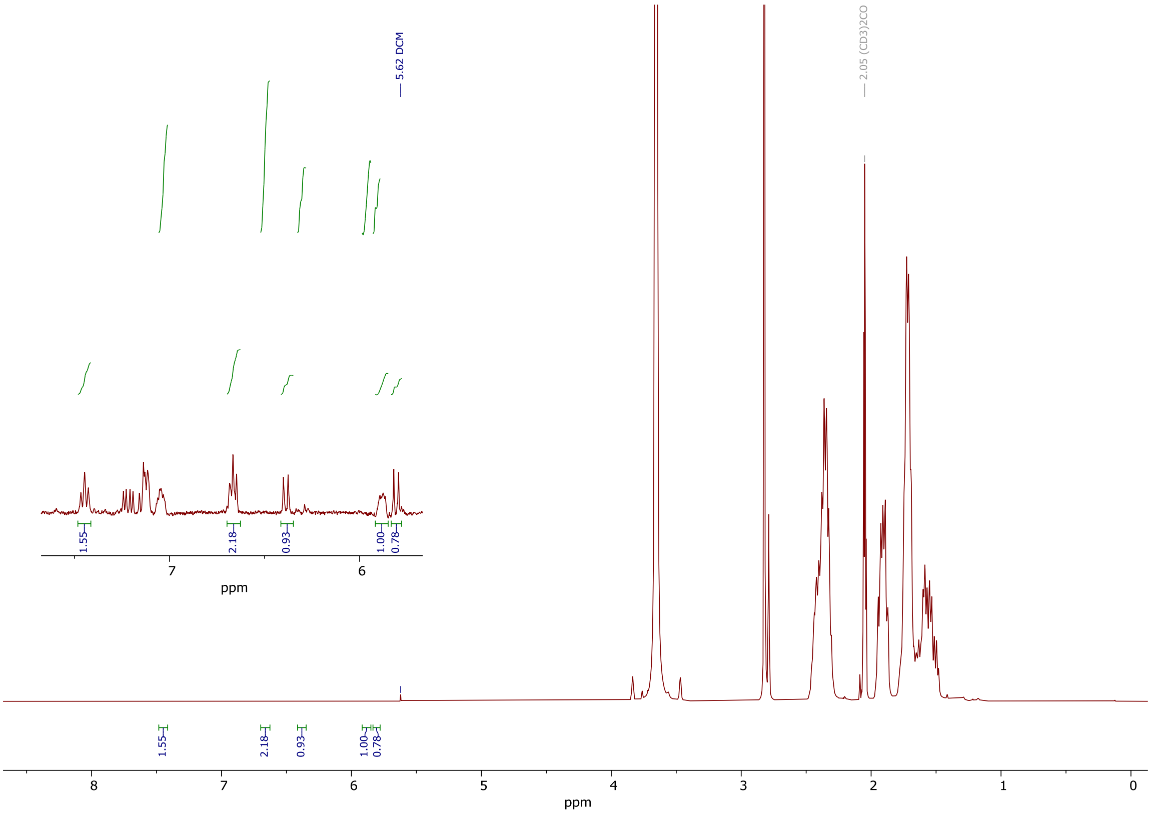

Spectrum S49. ^{1}H NMR (400 MHz, Acetone-d_6, 298 K) spectrum of post-sonication polymer **1** with **3-Methyl-2-butenal** after being washed with methanol.

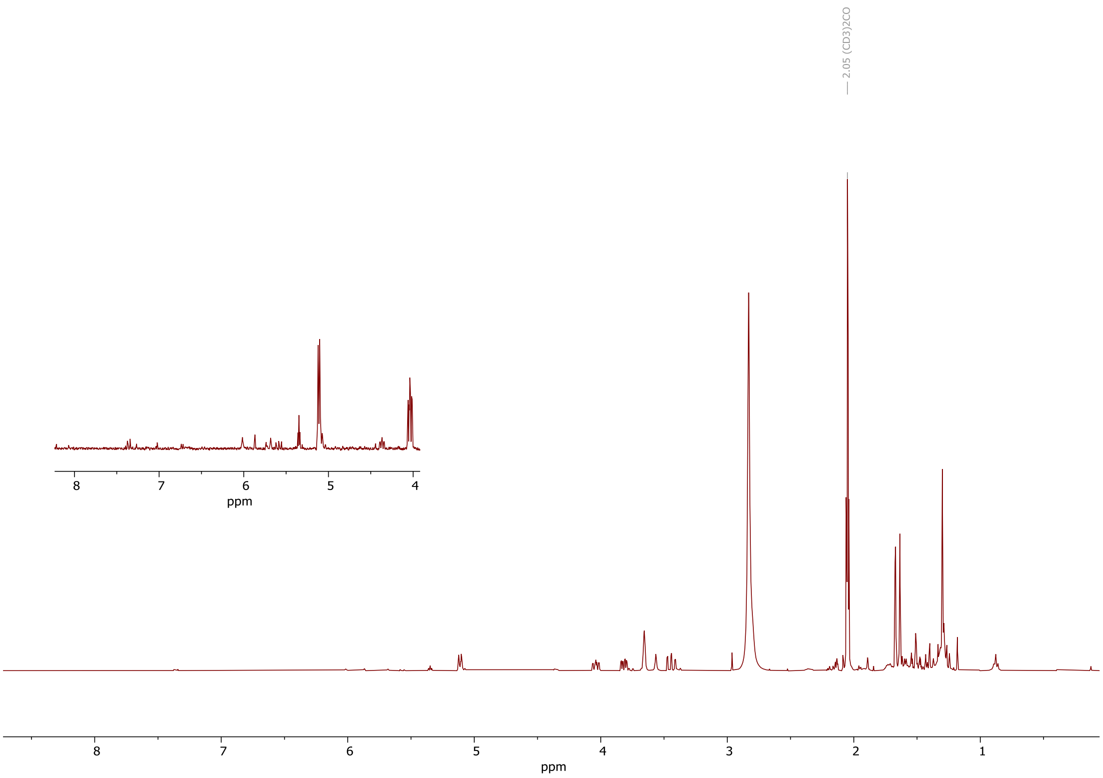

Spectrum S50. ^{1}H NMR (400 MHz, Acetone-d_6, 298 K) spectrum of concentrated methanol washings from post-sonication polymer **1** with **3-Methyl-2-butenal**.

6.3.4 Post-sonication ^{1}H NMR Spectra of Polymer 1 with 1H-Pyrazole

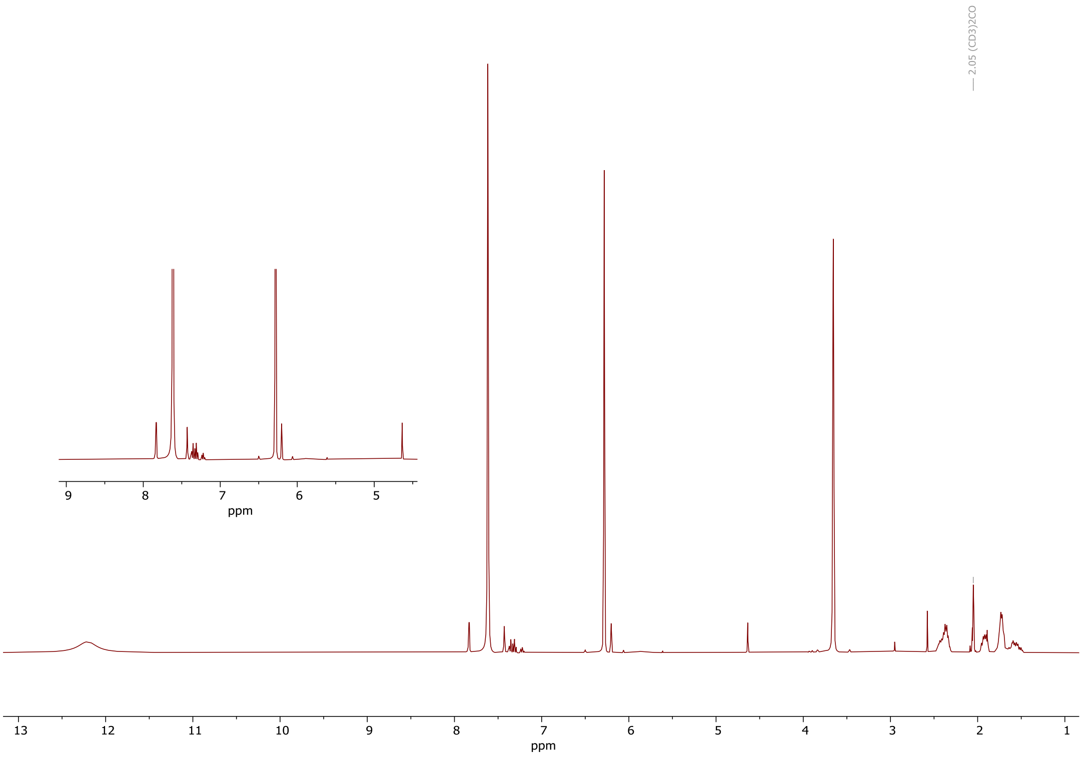

Spectrum S51. ^{1}H NMR (400 MHz, Acetone-d_6, 298 K) spectrum of post-sonication polymer **1** with **1H-Pyrazole** before being washed with methanol.

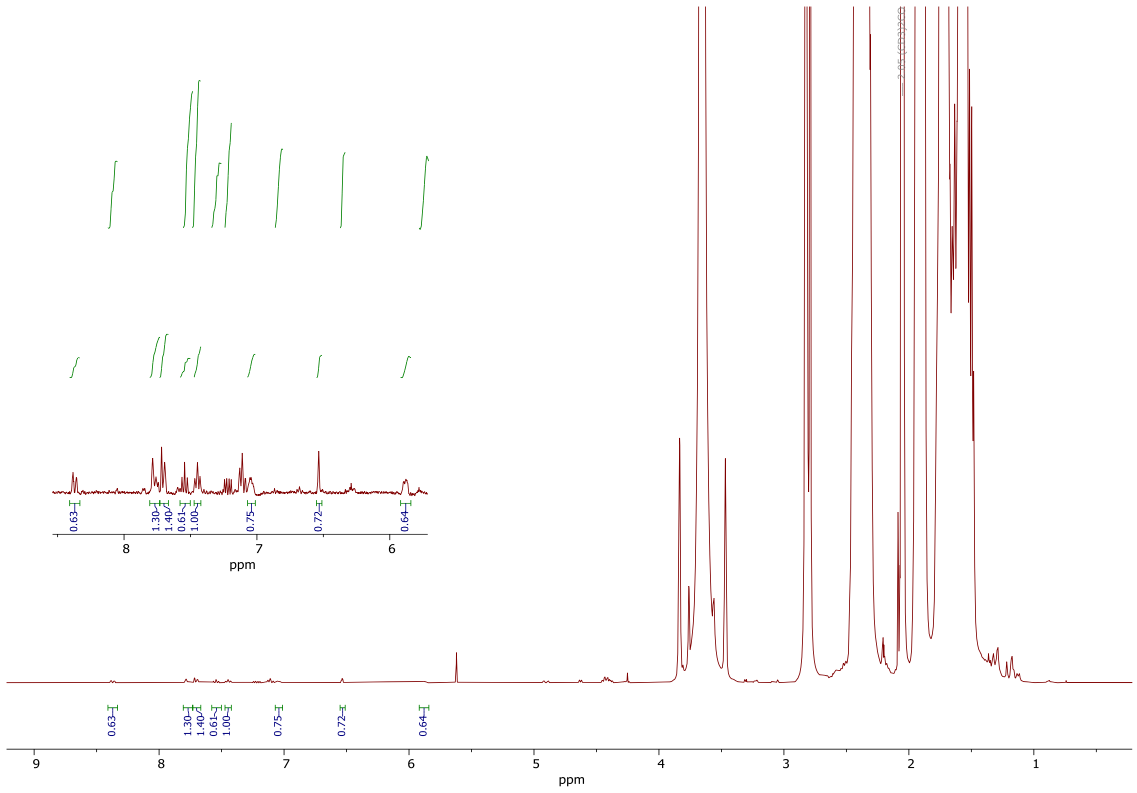

Spectrum S52. ^{1}H NMR (400 MHz, Acetone-d_6, 298 K) spectrum of post-sonication polymer **1** with **1H-Pyrazole** after being washed with methanol.

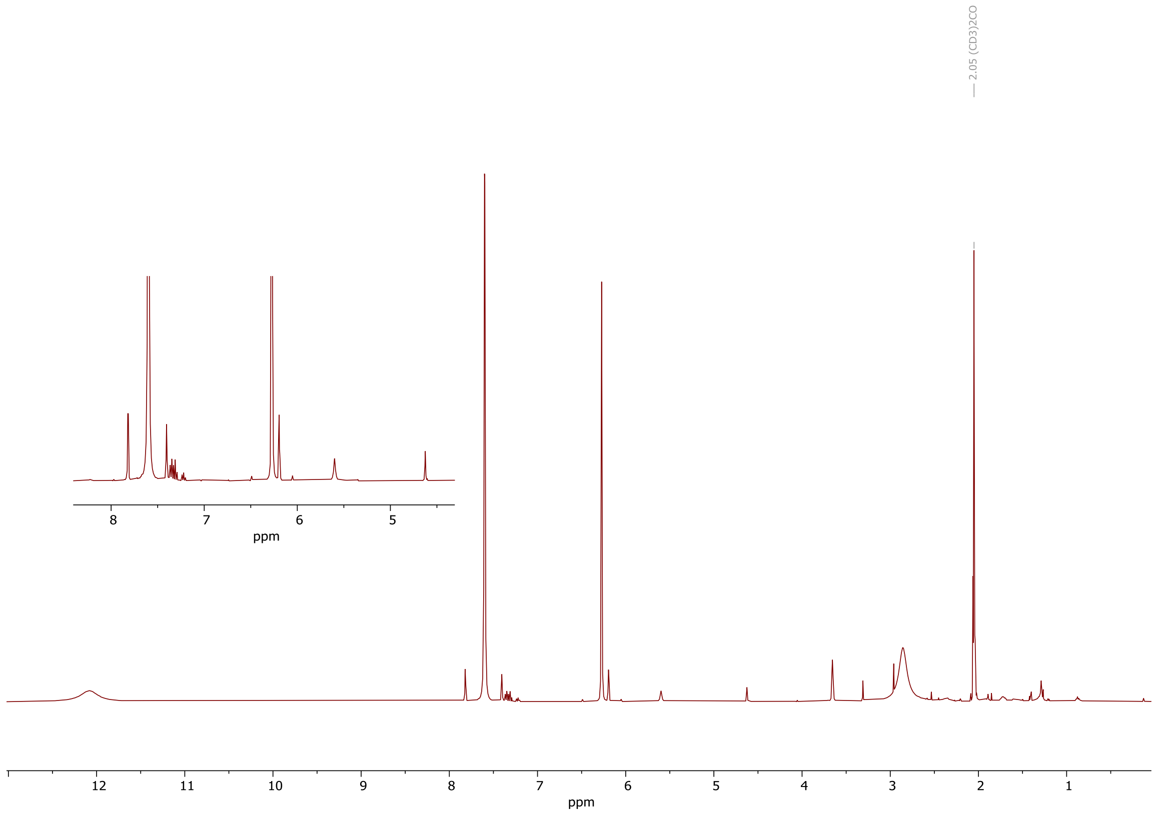

Spectrum S53. ^{1}H NMR (400 MHz, Acetone-d_6, 298 K) spectrum of concentrated methanol washings from post-sonication polymer **1** with **1H-Pyrazole**.

6.3.5 *Post-sonication ¹H NMR Spectra of Polymer S20*

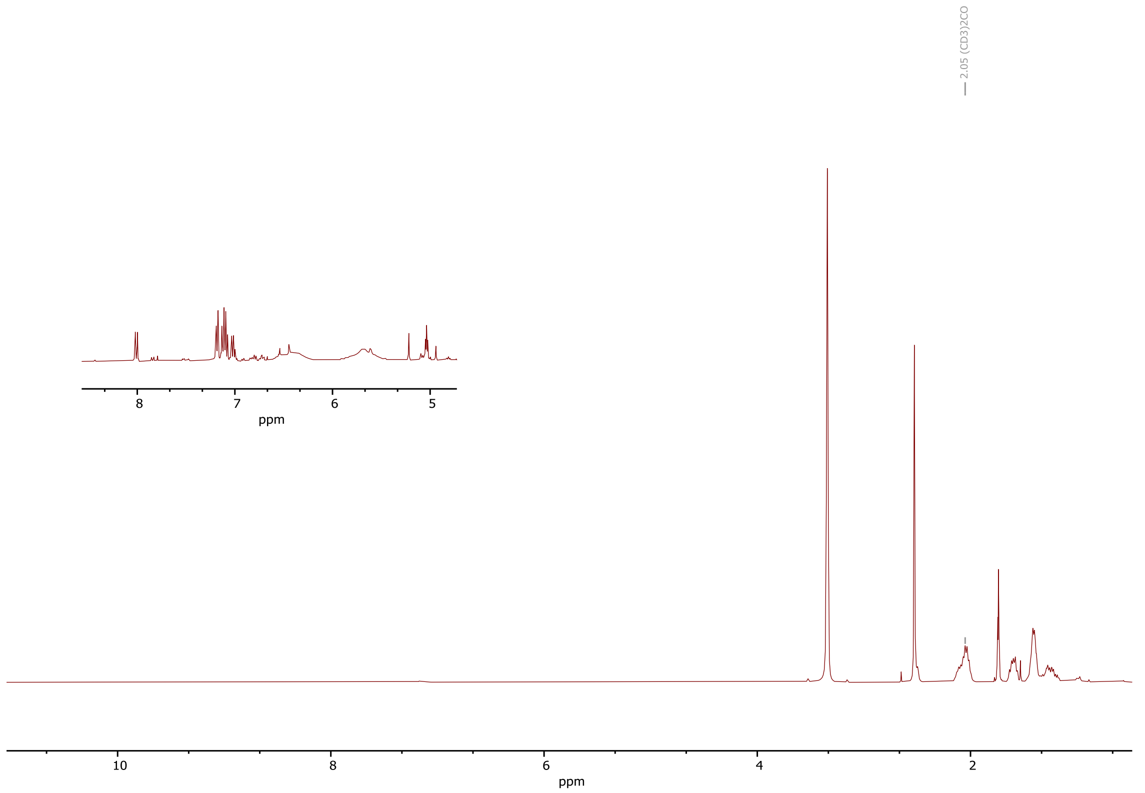

Spectrum S54. ^{1}H NMR (500 MHz, Acetone-d_6, 298 K) spectrum of post-sonication polymer **S18** before being washed with methanol.

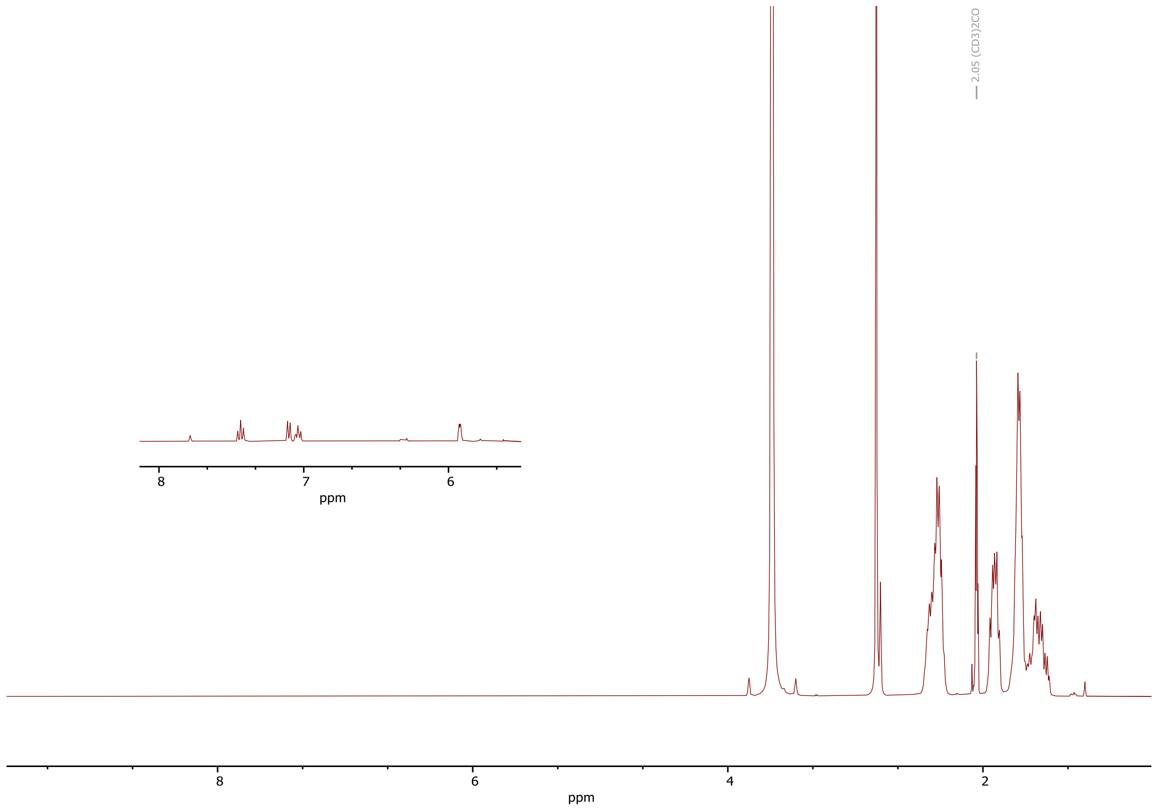

Spectrum S55. ^{1}H NMR (500 MHz, Acetone-d_6, 298 K) spectrum of post-sonication polymer **S18** after being washed with methanol.

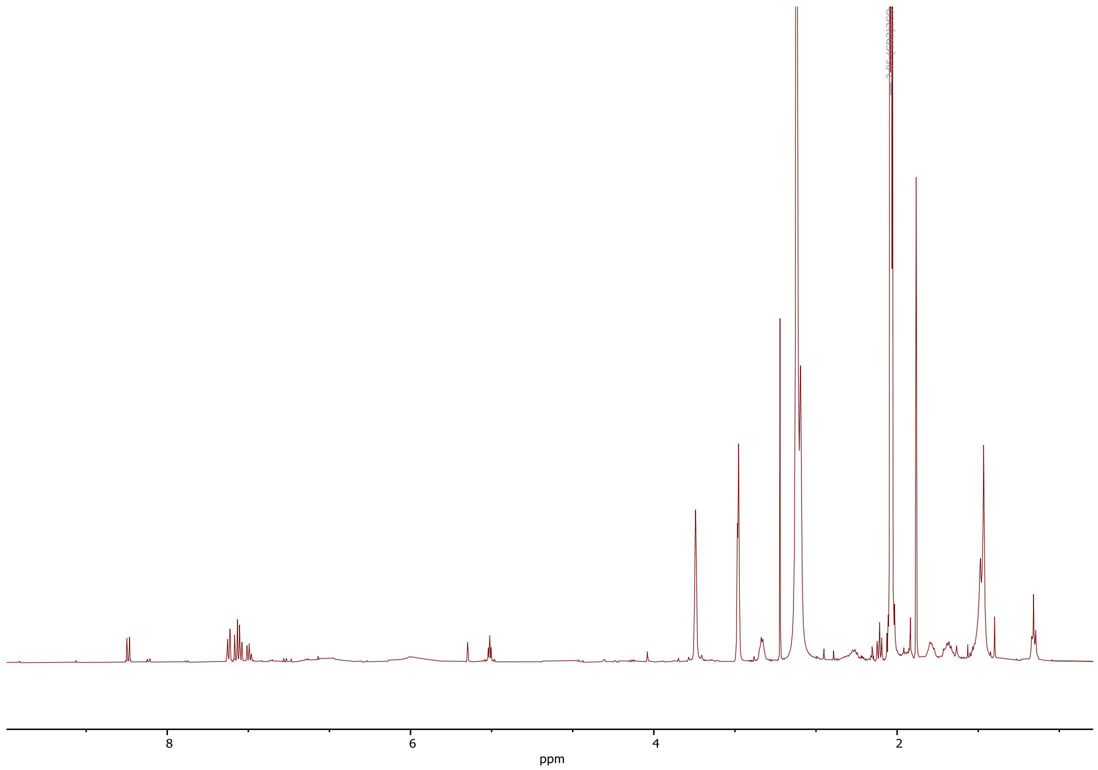

Spectrum S56. ^{1}H NMR (400 MHz, Acetone-d_6, 298 K) spectrum of concentrated methanol washings from post-sonication polymer **S18**.

7. Crystal Structures

Single crystals of compounds **5** was obtained by recrystallization (PE:EtOAc 30:1). **S1** were obtained by diffusion of hexanes into a concentrated solution of **S1** in ethyl acetate over 14 days.

7.1 Crystal Structure of 5

Table S2. Summary of crystal data for compound **5**.

CCDC deposition number	2295694
Identification code	**5**
Empirical formula	$C_{15}H_4O_2$
Formula weight	226.26
Crystal color	Clear colorless
Temperature/K	100
Crystal system	monoclinic
Space group	P 1 21/n 1
a/Å	21.0807(4)
b/Å	4.92885(12)
c/Å	23.4456(5)
α/°	90
β/°	102.218(2)
γ/°	90
Volume/Å^3	2380.91(9)
Z	8
ρ_{calc} g/cm^3	1.262
Radiation	CuKα (λ = 1.54184)
Goodness-of-fit	1.047
R (reflections)	0.0578(4198)

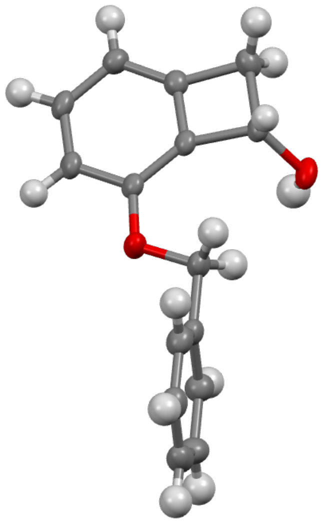

Crystal structure of **S2** showing 50% thermal ellipsoid probability.

7.2 Crystal Structure of Dimer S1

Table S3. Summary of crystal data for compound **S1**.

CCDC deposition number	2295695
Identification code	**S1**
Empirical formula	$C_{30}H_{28}O_4$
Formula weight	452.52
Crystal color	Clear light colorless
Temperature/K	100
Crystal system	monoclinic
Space group	P 1 21/n 1
a/Å	23.5231 (19)
b/Å	8.2852 (7)
c/Å	24.9724 (19)
α/°	90
β/°	105.323 (8)
γ/°	90
Volume/Å^3	4694.0 (7)
Z	8
ρ_{calc} g/cm^3	1.281
Radiation	CuKα (λ = 1.54184)
Goodness-of-fit	1.029
R (reflections)	0.1398 (9419)

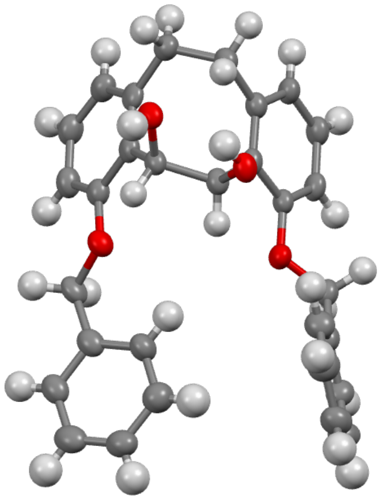

Crystal structure of **S1** showing 50% thermal ellipsoid probability.

8. Calculations

CoGEF calculations were performed on Spartan'20 following Beyer's method.[6] The structure of the mechanophore was built in Spartan'20 and minimized using molecular mechanics (MMFF). The distance between the terminal methylene groups was constrained and increased by increments of 0.1 Å, and the energy was minimized by DFT (B3LYP/6-31G*, gas) at each step using the *Energy Profile* function implemented in Spartan'20. The relative energy of each intermediate was determined by setting the energy of the initial state at 0 kJ/mol. F_{max} values were determined from the slope of the final 40% of the energy/elongation curve (i.e. from 0.6 E_{max} to E_{max}).

8.1 CoGEF of BCB model 1'$_O$

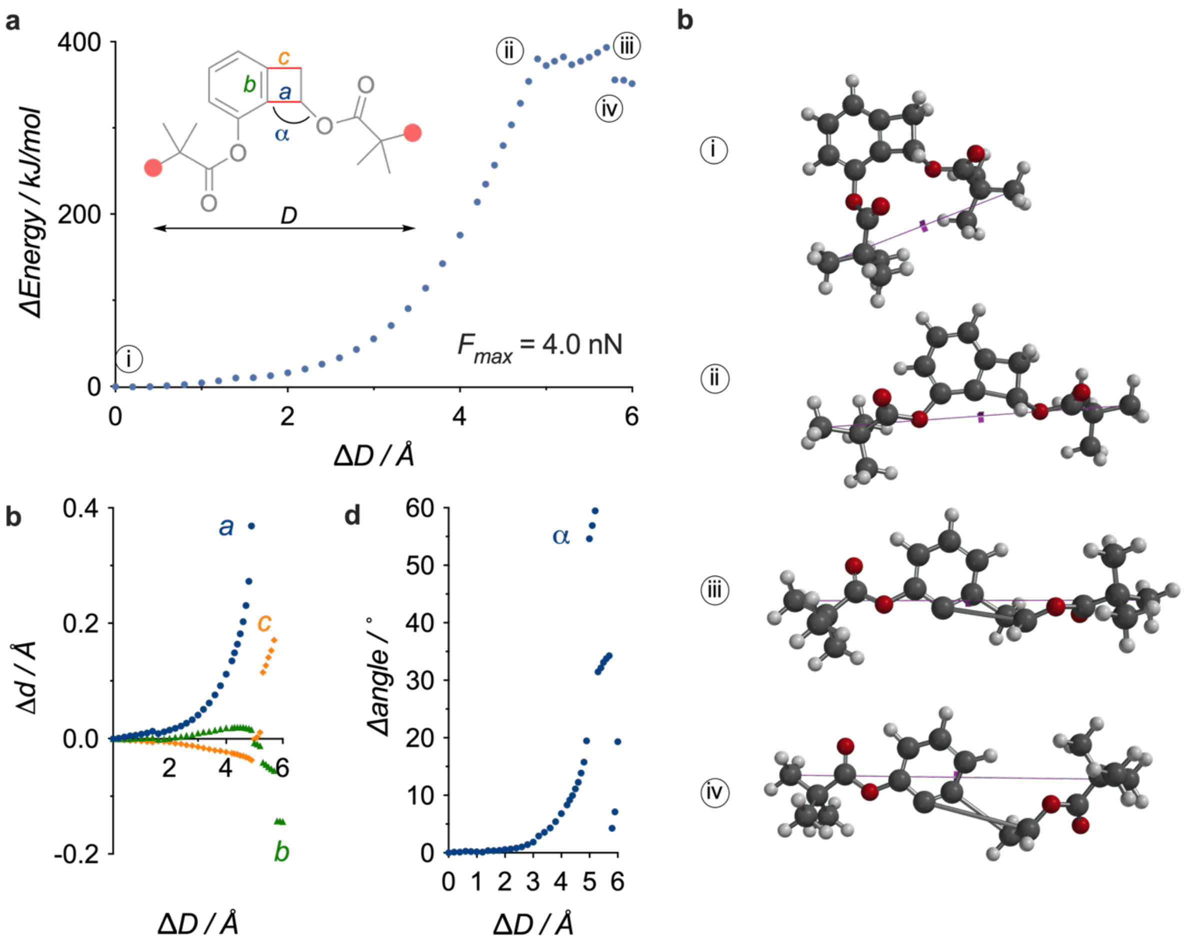

Figure S14. Computational investigation of BCB model **1'$_O$** with *ortho* substitution pattern. (a) Evolution of energy upon simulated elongation (CoGEF, DFT B3LYP/6-31G*, vac.) of a model of the BCB mechanophore. Scissile bonds are shown in red. (b) Equilibrium geometries at E_0 (i), E_{max1} (ii), E_{max2} (iii), and after dissociation (iv). (c) Elongation of bonds *a, b, c*, and (d) opening of angle upon simulated elongation of the same model.

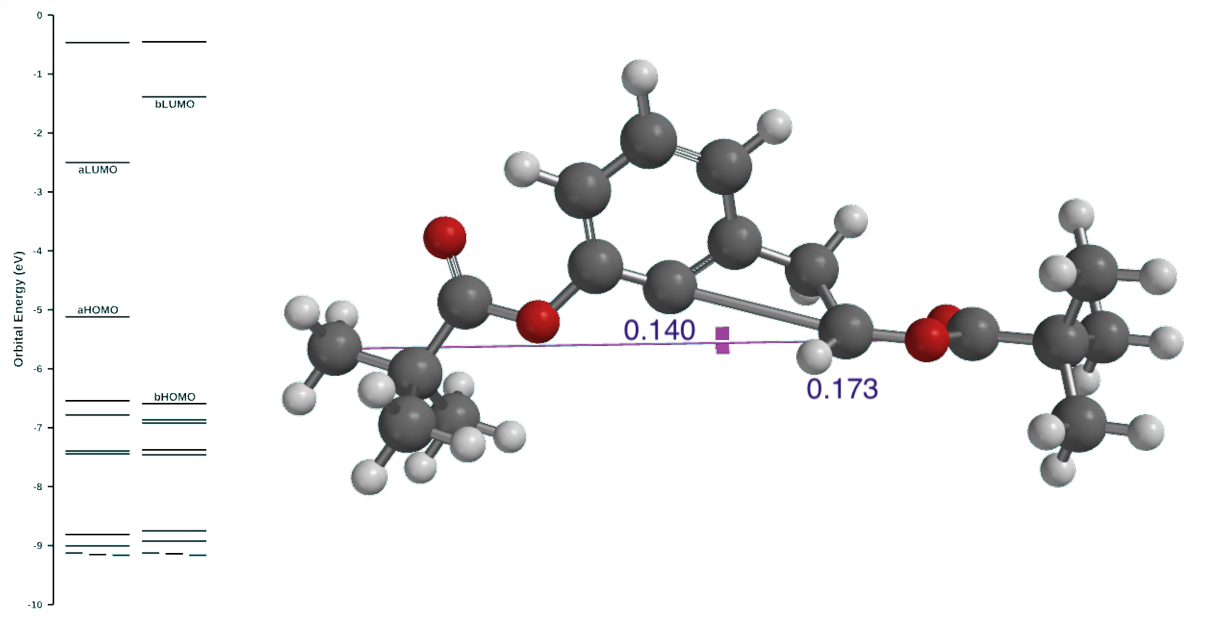

Figure S15. Orbitals diagram and natural charges at C on either side of the broken bond suggest a homolytic scission of the first C-C bond. Unrestricted DFT (UB3LYP/6-31G*, vac.) model of the first broken intermediate from the CoGEF of **1’**$_{o}$**.**

8.2 CoGEF of BCB model 1'$_m$

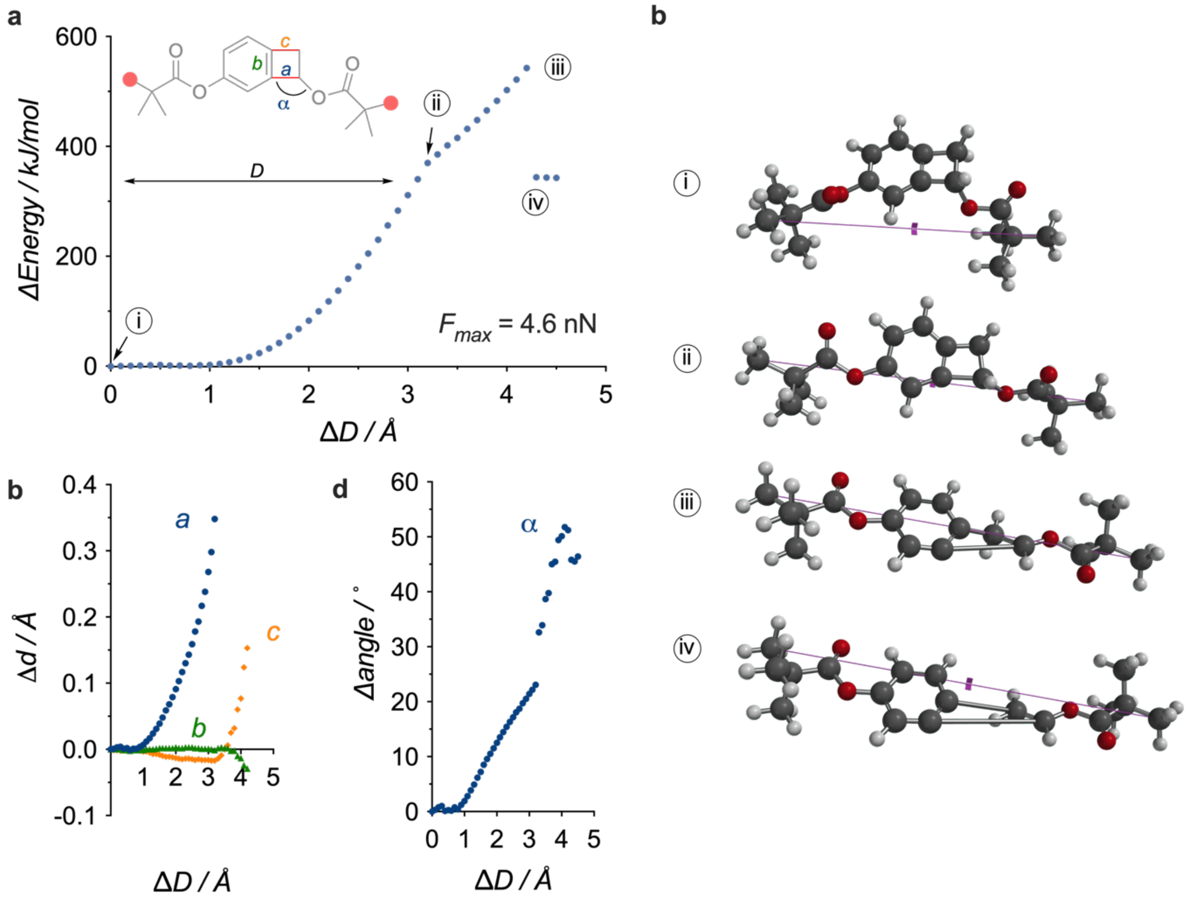

Figure S16. Computational investigation of BCB model **1'$_m$** with *meta* substitution pattern. (a) Evolution of energy upon simulated elongation (CoGEF, DFT B3LYP/6-31G*, vac.) of a model of the BCB mechanophore. Scissile bonds are shown in red. (b) Equilibrium geometries at E_0 (i), E_{max1} (ii), E_{max2} (iii), and after dissociation (iv). (c) Elongation of bonds *a, b, c*, and (d) opening of angle upon simulated elongation of the same model.

8.3 CoGEF of BCB model 1’$_p$

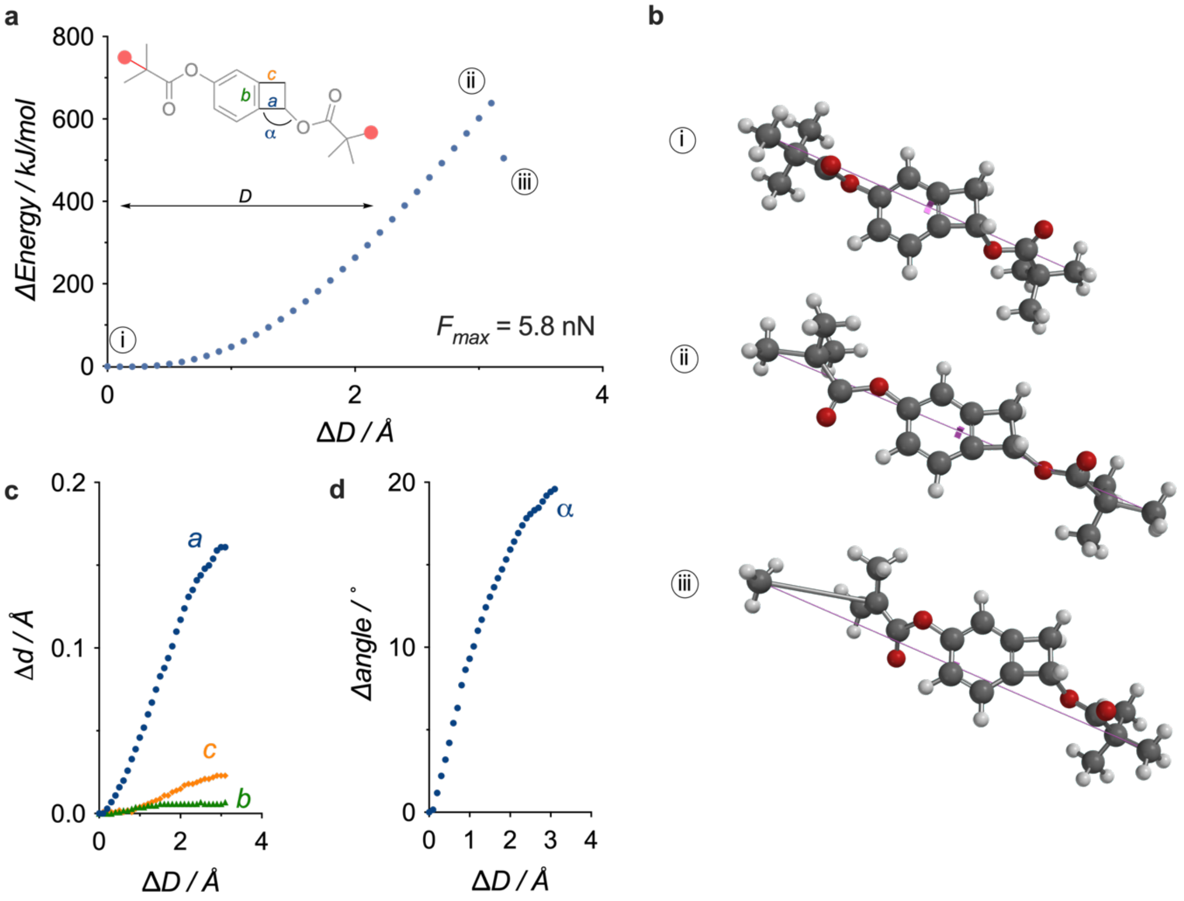

Figure S17. Computational investigation of BCB model **1’$_p$** with *para* substitution pattern. (a) Evolution of energy upon simulated elongation (CoGEF, DFT B3LYP/6-31G*, vac.) of a model of the BCB mechanophore. Scissile bonds are shown in red. (b) Equilibrium geometries at E_0 (i), E_{max1} (ii), E_{max2} (iii), and after dissociation (iv). (c) Elongation of bonds *a, b, c*, and (d) opening of angle upon simulated elongation of the same model.

8.4 CoGEF of BCB model **1'$_{m'}$**

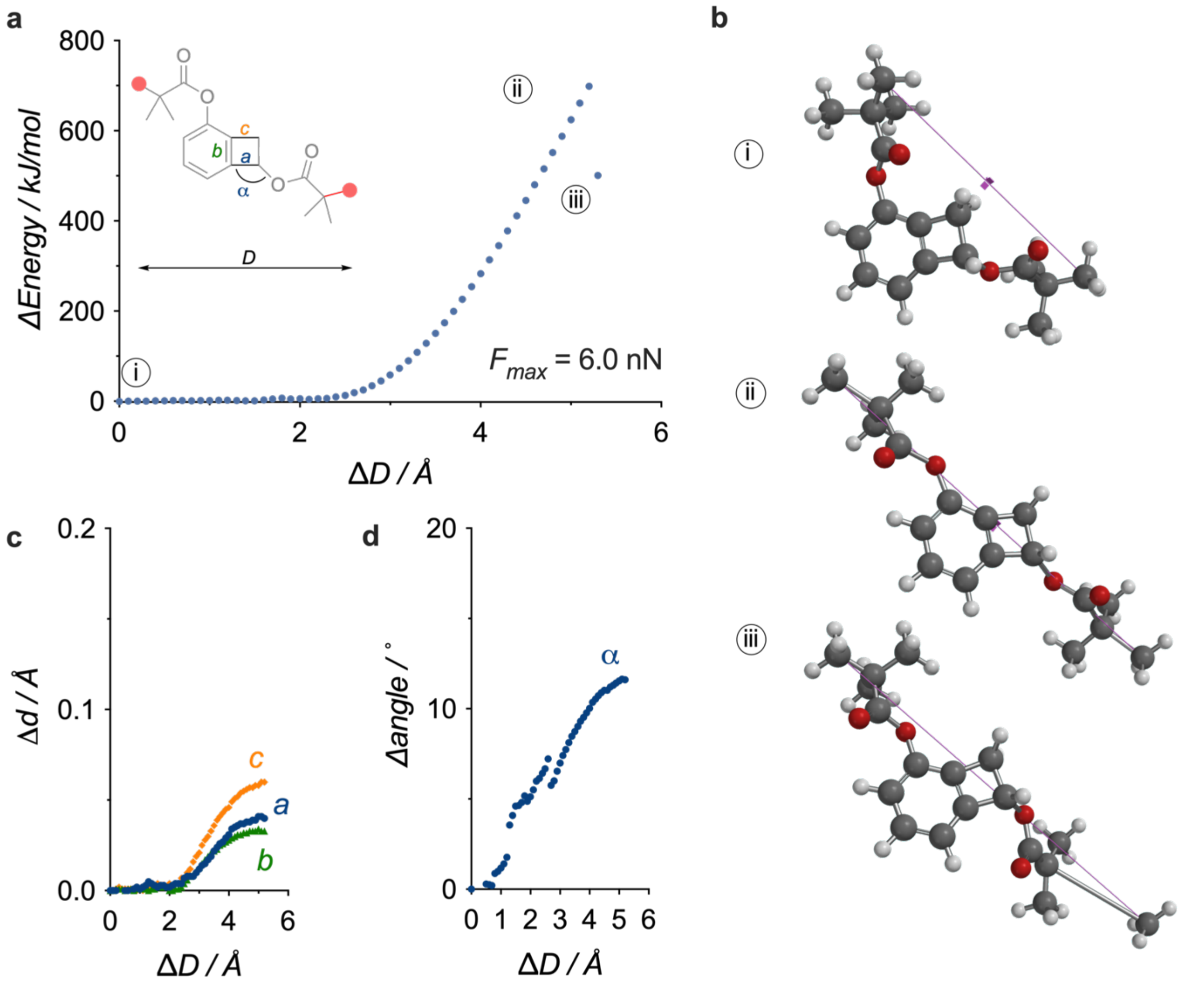

Figure S18. Computational investigation of BCB model **1'$_{m'}$** with *meta'* substitution pattern. (a) Evolution of energy upon simulated elongation (CoGEF, DFT B3LYP/6-31G*, vac.) of a model of the BCB mechanophore. Scissile bonds are shown in red. (b) Equilibrium geometries at E_0 (i), E_{max1} (ii), E_{max2} (iii), and after dissociation (iv). (c) Elongation of bonds *a, b, c*, and (d) opening of angle upon simulated elongation of the same model.

9. References

1. Kern, N.; Dombray, T.; Blanc, A.; Weibel, J.; Pal, P. Silver(I)-catalyzed deprotection of p-methoxybenzyl ethers: a mild and chemoselective method. *J. Org. Chem.,* **2012***, 77,* 9227−9235.
2. Xu. H.; He. J.; Shi. J.; Tan. L.; Qiu. D.; Luo. X.; Li. Y. Domino Aryne Annulation via a Nucleophilic−Ene Process. *J. Am. Chem. Soc.,* **2018**, *140*, 3555−3559.
3. Giovani. S.; Singh. R.; Fasan. R. Efficient conversion of primary azides to aldehydes catalyzed by active site variants of myoglobin. *Chem. Sci*., **2016**, *7*, 234.
4. Hickenboth, C. R.; Moore, J. S.; White, S. R.; Sottos, N. R.; Baudry, J.; Wilson, S. R. Biasing reaction pathways with mechanical force. *Nature.,* **2007**, *446*, 423−427.
5. Chen, P. H.; Savage, N. A.; Dong G. Concise synthesis of functionalized benzocyclobutenones. *Tetrahedron.,* **2014**, *70*, 4135−4146.
6. Beyer, M. J. The mechanical strength of a covalent bond calculated by density functional theory. *Chem. Phys*., **2000**, *112*, 7307–7312.
